# Utility–Privacy Trade-Offs with Limited Leakage for Encoder

**DOI:** 10.3390/e25060921

**Published:** 2023-06-11

**Authors:** Naruki Shinohara, Hideki Yagi

**Affiliations:** Department of Computer and Network Engineering, The University of Electro-Communications, 1-5-1 Chofugaoka, Chofu 182-8585, Tokyo, Japan; s1710291@mail.uec.jp

**Keywords:** utility–privacy trade-offs, source coding, Shannon theory, strong converse theorem

## Abstract

The utilization of databases such as IoT has progressed, and understanding how to protect the privacy of data is an important issue. As pioneering work, in 1983, Yamamoto assumed the source (database), which consists of public information and private information, and found theoretical limits (first-order rate analysis) among the coding rate, utility and privacy for the decoder in two special cases. In this paper, we consider a more general case based on the work by Shinohara and Yagi in 2022. Introducing a measure of privacy for the encoder, we investigate the following two problems: The first problem is the first-order rate analysis among the coding rate, utility, privacy for the decoder, and privacy for the encoder, in which utility is measured by the expected distortion or the excess-distortion probability. The second task is establishing the strong converse theorem for utility–privacy trade-offs, in which utility is measured by the excess-distortion probability. These results may lead to a more refined analysis such as the second-order rate analysis.

## 1. Introduction

### 1.1. Background

**Figure 1:**
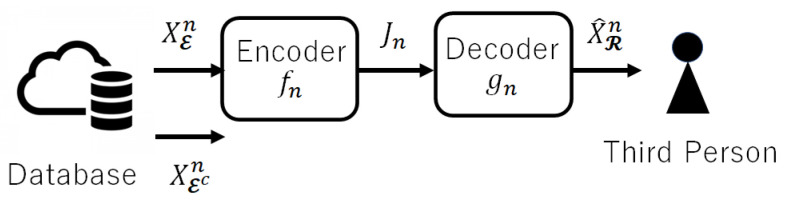
Privacy-constrained coding system.

The utilization of database has progressed in our society and includes autonomous cars and the congestion data service over the Internet. At the same time, the risk of accidental or intentional leakage of private information has also increased rapidly. To protect private information, coding with a privacy constraint has been analyzed via an information-theoretic approach. In 1983, Yamamoto [1] introduced a framework to quantify the utility of databases and the privacy of personal information and analyzed the trade-offs between them. Decades later, in 2013, Sankar et al. [2] claimed the necessity of converting databases to protect privacy while maintaining the utility of data. Then, Yamamoto’s framework [1] was re-recognized by Sankar et al. and other researchers. Using the **rate-distortion theory** in information theory, he revealed the optimal relationships (theoretical limits) among coding rate, utility, and privacy in two cases; (i) public information that can be open to the public and private information that should be protected from a third party are encoded, and (ii) only public information is encoded. However, since a more general case, i.e., where (iii) public information and a part of private information is encoded, had not been clarified, Shinohara and Yagi [3] derived the theoretical limits in such a case (see Figure 1). As a result, our characterization of the achievable region gives a “unified expression” because it includes the characteristics given in [1] in cases (i) and (ii) as special cases.

### 1.2. Motivation and Contributions

By investigating case (iii), one can compare the theoretical limits corresponding to a variety of patterns of the encoded information. One can see that the achievable region in case (i) is the largest among all patterns. However, this may not be the case if **privacy leakage for the encoder** is constrained. Motivated by this observation, in this paper, we characterize the optimal trade-offs among coding rate, utility, privacy for the decoder, and **privacy for the encoder** in Section 3. The addressed problem corresponds to the case where there are some aggregators between the source and the encoder and the aggregator controls the data (source sequence) passing to the encoder. The obtained results indeed suggest that the best-encoded information can be in case (iii) if some restriction is imposed on the privacy leakage for the encoder.

One of the most important tasks in information-theoretic analysis for utility–privacy trade-offs is **second-order rate analysis** (e.g., [4,5,6]). In general, in second-order rate analysis, the **excess-distortion probability** is used as a measure of utility [4,5,6]. However, in the **first-order rate analysis** shown in [3], utility is measured by the **expected distortion**, so for second-order rate analysis, we need first to conduct first-order rate analysis, which replaces the expected distortion with the excess-distortion probability as the measure of utility. In Section 4, the theoretical limits coincide with the one in which utility is measured by expected distortion.

There is one more problem to solve before tackling second-order rate analysis: we need to clarify whether the boundary of the achievable region may vary or not, depending on the value of the excess-distortion probability. In Section 5, we establish the **strong converse** theorem, provided that utility is measured by the probability of excess distortion. For the sake of simplicity, we focus on the achievable region of utility and privacy for the decoder or a third party, which reveals an aspect of utility–privacy trade-offs. In the proof, we adopt a change in measure argument developed by Tyagi and Watanabe [7]. Contrary to the standard rate-distortion problem, the alphabets of the encoder’s input and the decoder’s output are different, so we extend the argument to incorporate this discrepancy. Although the strong converse theorem is shown for the rate region of utility and privacy, we can also derive the same result when the privacy of the encoder is involved.

**Figure 2:**
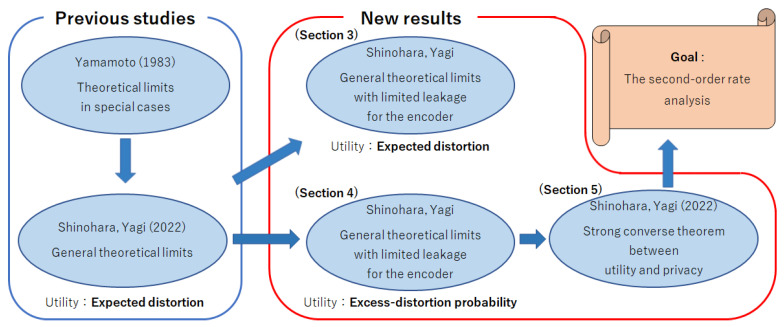
Road map for second-order rate analysis [1,3,8].

For readers’ convenience, Figure 2 shows the road map to the most important task: the second-order rate analysis. In summary, three contributions of this paper are as follows:

1. The rate analysis among the coding rate, utility, privacy for the decoder, and privacy for the encoder in which utility is measured using the expected distortion (Section 3).
2. The rate analysis among the coding rate, utility, privacy for the decoder, and privacy for the encoder in which utility is measured using the excess-distortion probability (Section 4).
3. The strong converse theorem for utility–privacy trade-offs in which utility is measured using the excess-distortion probability (Section 5).

### 1.3. Related Work

The analysis of the utility–privacy trade-offs using an information-theoretic approach was initiated by [2], which translates the rate-distortion problem with an equivocation constraint in [1] into the privacy and utility trade-off problem. In information-theoretic studies on coding with privacy and utility constraints, several measures for privacy and utility are adopted. One of the strong measures for privacy is differential privacy [8,9], and an extension and relaxation of differential privacy have been proposed in [10,11]. A weaker but useful privacy measure is the mutual information between the codeword and private information [1,2,12,13,14], which guarantees the average amount of leaked private information. Other examples of well-known privacy measures are maximal leakage [15], maximal α-leakage [16,17,18], and total variation [19]. Relationships among several measures for privacy have been revealed in [20]. On the other hand, well-known utility measures are average distortion [1,2,3,4,5,6,7,8,9,10,11,12,13,14,15,16,17,18,19,20,21,22,23,24,25], hard distortion [16,17], and log-loss distortion [26].

Coding systems in the utility–privacy problem are extended to the ones with the encoder’s side information [2] and with the decoder’s side information [25]. In [14], a related coding problem has been investigated, where both the encoder and the decoder can access a uniform secret key and the decoder can also access side information. Utility–privacy trade-off schemes are applied, for example, to the Internet of Energy [23] and to a system with informational self-determination [24].

A closely related study to this paper was given by Basciftci et al. [13], in which several release mechanisms of encoded information from the database were discussed. In particular, utility–privacy trade-offs (without the coding rate) were compared when the encoded information was (i) both private and public information, (ii) only public information, and (iv) only private information (see also the three cases described in Section 1.1). A sufficient condition under which the utility–privacy trade-offs coincide for cases (i) and (ii) was given.

### 1.4. Organization

This paper is organized as follows: In Section 2, we begin by introducing the notation and system model that are used in this paper. In Section 3, we give the first-order rate analysis among the coding rate, utility, privacy for the decoder, and privacy for the encoder in which utility is measured by the expected distortion. In Section 4, we tackle the first-order rate analysis among the coding rate, utility, privacy for the decoder, and privacy for the encoder in which utility is measured by the excess-distortion probability. Section 5 focuses on the strong converse theorem for utility–privacy trade-offs in which utility is measured by the excess-distortion probability. In Section 6, we discuss the significance of the encoded information with limited leakage for the encoder. Finally, in Section 7, the conclusion and future work are stated.

## 2. Notation and System Model

### 2.1. Information Source

Database *d* is described by a K×n matrix whose rows represent *K* attributes and columns represent *n* entries of data. Let K={1,2,…,K} be the set of indexes of *K* attributes. The random variable for the *l*th attribute is denoted by $X_{l}$, which takes a value in a finite alphabet $X_{l}$. For any subset B⊆K, the tuple of random variables ${\left(X_{l}\right)}_{l\in B}$ is abbreviated as $X_{B}$. Similarly, the Cartesian product of alphabets $\prod_{l\in B}X_{l}$ is abbreviated as $X_{B}$.

The *K* attributes can be divided into two groups; one may be open to the public and the other should be kept secret from a third party. Then, the set K is divided into disjoint sets R and H. That is,

(1) $$\begin{array}{c} K=R\cup H,\;\;\;\;R\cap H=\emptyset ,\;\;\;\;X_{K}=X_{R}\times X_{H}, \end{array}$$

where $X_{R}$ is the set of values that public (revealed) source symbols $X_{R}$ take and $X_{H}$ is the set of values that private (hidden) source symbols $X_{H}$ take.

We assume that the source sequence $X_{K}^{n}=\left(X_{K,1},X_{K,2},\ldots ,X_{K,n}\right)$ is generated from a stationary and memoryless source $p_{X_{K}}$. That is,

(2) $$\begin{array}{c} P_{X_{K}^{n}}\left(x_{K}^{n}\right)=\mathrm{Pr}\left\{X_{K}^{n}=x_{K}^{n}\right\}=\prod_{i=1}^{n}P_{X_{K}}\left(x_{K,i}\right), \end{array}$$

where $x_{K}^{n}=\left(x_{K,1},\ldots ,x_{K,n}\right)\in X_{K}^{n}$. Taking the partition of attributes in (1) into account, the source sequence $X_{K}^{n}$ is described as

(3) $$\begin{array}{c} X_{K}^{n}=\left(X_{R}^{n},X_{H}^{n}\right), \end{array}$$

where

(4) $$\begin{array}{cc} \; & X_{R}^{n}=\left(X_{R,1},X_{R,2},\ldots ,X_{R,n}\right)\in X_{R}^{n}, \end{array}$$

(5) $$\begin{array}{cc} \; & X_{H}^{n}=\left(X_{H,1},X_{H,2},\ldots ,X_{H,n}\right)\in X_{H}^{n} \end{array}$$

are referred to as the revealed source sequence and the hidden source sequence, respectively. In the addressed coding system introduced in [22], the revealed symbols and a part of the hidden symbols are input to the encoder, and thus the encoded alphabet E satisfies R⊆E⊆K. Similar to (3), $X_{K}^{n}$ is sometimes described as

(6) $$\begin{array}{c} X_{K}^{n}=\left(X_{E}^{n},X_{E^{c}}^{n}\right), \end{array}$$

where $X_{E}^{n}$ is the source sequence observed by the encoder and $E^{c}=K\setminus E$.

### 2.2. Encoder and Decoder

The coding system consists of encoder $f_{n}$ and decoder $g_{n}$ as in Figure 1. When the source sequence $X_{K}^{n}=\left(X_{E}^{n},X_{E^{c}}^{n}\right)$ is generated from the stationary and memoryless source $p_{X_{K}}$, the codeword $J_{n}=f_{n}\left(X_{E}^{n}\right)$ is generated by the encoder

(7) $$\begin{array}{c} f_{n}\;:\;X_{E}^{n}\to \left\{1,2,\ldots ,M_{n}\right\} \end{array}$$

and the reproduced sequence ${\hat{X}}_{R}^{n}=g_{n}\left(J_{n}\right)$ is produced by decoder

(8) $$\begin{array}{c} g_{n}\;:\;\left\{1,2,\ldots ,M_{n}\right\}\to {\hat{X}}_{R}^{n}, \end{array}$$

where $M_{n}$ denotes the number of codewords.

## 3. First-Order Rate Analysis with Expected Distortion

### 3.1. Performance Measures

In this section, we mention the measure of the coding rate, utility, privacy for the decoder, and privacy for the encoder. Hereafter, let a pair of the encoder and decoder $\left(f_{n},g_{n}\right)$ be fixed.

For a given $M_{n}$, the coding rate is defined as

(9) $$\begin{array}{c} r_{n}≔\frac{1}{n}\log M_{n}. \end{array}$$

Let $d:\;X_{R}\times {\hat{X}}_{R}\to \left[0,\infty \right)$ be a distortion function between $x_{R}\in X_{R}$ and ${\hat{x}}_{R}\in {\hat{X}}_{R}$. The distortion between sequences $x_{R}^{n}\in X_{R}^{n}$ and ${\hat{x}}_{R}^{n}\in {\hat{X}}_{R}^{n}$ is defined as

(10) $$\begin{array}{cc} d(x_{R}^{n},{\hat{x}}_{R}^{n})≔ & \sum_{i=1}^{n}d\left(x_{R,i},{\hat{x}}_{R,i}\right). \end{array}$$

Then, the measure of utility is defined as

(11) $$\begin{array}{cc} u_{n}≔ & E\left[\frac{1}{n}d\left(X_{R}^{n},{\hat{X}}_{R}^{n}\right)\right], \end{array}$$

where E represents the expectation by the joint distribution of $\left(X_{R}^{n},{\hat{X}}_{R}^{n}\right)$.

In this system, the privacy of the hidden source sequence $X_{H}^{n}$ should be protected when the codeword $J_{n}$ is observed by decoder $g_{n}$. The measure of privacy for the decoder is defined as

(12) $$\begin{array}{c} l_{n}≕\frac{1}{n}I\left(X_{H}^{n};J_{n}\right), \end{array}$$

where $I(X_{H}^{n};J_{n})$ is the mutual information between $X_{H}^{n}$ and $J_{n}$.

The privacy of the hidden source sequence $X_{H}^{n}$ should be protected when the encoded information $X_{E}$ is observed by encoder $f_{n}$. The measurement of privacy for the encoder is defined as

(13) $$\begin{array}{c} e_{n}≔\frac{1}{n}I\left(X_{H}^{n};X_{E}^{n}\right), \end{array}$$

where $I(X_{H}^{n};X_{E}^{n})$ is the mutual information between $X_{H}^{n}$ and $X_{E}^{n}$.

### 3.2. Achievable Region and Theorem

We define the achievable region for the first-order rate analysis with the expected distortion and state the obtained results.

**Definition** **1.** *A tuple (R,D,L,E) is said to be* ϵ***-achievable*** *(with respect to the expected distortion measure) if, for any given ϵ>0, there exists a sequence of codes $\left(f_{n},g_{n}\right)$ satisfying*

(14) $$\begin{array}{cc} r_{n} & \leq R+\epsilon , \end{array}$$

(15) $$\begin{array}{cc} u_{n} & \leq D+\epsilon , \end{array}$$

(16) $$\begin{array}{cc} l_{n} & \leq L+\epsilon , \end{array}$$

(17) $$\begin{array}{cc} e_{n} & \leq E+\epsilon \end{array}$$

*for all sufficiently large n.*

The technical meanings of each constraint in Definition 1 can be interpreted as follows: Equation (14) evaluates how much the source sequence is compressed, so this rate should be decreased. Equation (15) is the constraint corresponding to distortion being less than D+ϵ. The smaller the distortion is, the better the utility is, so this condition should also be decreased. Equation (16) constrains the amount of leaked private information to the decoder. Since private information should be kept secret for the receiver, this quantity should be decreased as well. Equation (17) constrains the amount of private information leaked to the encoder. For the same reason as (16), this quantity should also be decreased.

**Remark** **1.** *The minimum coding rate R for a fixed D corresponds to the rate-distortion function (Section 10 in [27]). Thus, in the proof of achievability, we evaluate the coding rate and the distortion with the argument in rate-distortion theory. This view is also important to correctly understand the numerical results in Section 6.1.*

**Definition** **2.** *The closure of the set of ϵ-achievable tuples (R,D,L,E) is referred to as the* ϵ***-achievable region*** *and is denoted by $C_{E}\left(\epsilon |P_{X_{K}}\right)$ and defines*

(18) $$\begin{array}{c} C_{E}\left(P_{X_{K}}\right)≔\underset{0<\epsilon <1}{⋂}C_{E}\left(\epsilon |P_{X_{K}}\right). \end{array}$$

To characterize the achievable region, we define the following informational region.

**Definition** **3.** *For any E such that R⊆E⊆K, $S_{E}\left(P_{X_{K}}\right)$ is defined as*

(19) $$\begin{array}{cc} S_{E}\left(P_{X_{K}}\right)=\{\left(R,D,L,E\right):\;\;\; & R\geq I(X_{E};{\hat{X}}_{R}),\\ \; & D\geq E[d\left(X_{R},{\hat{X}}_{R}\right)],\\ \; & L\geq I(X_{H};{\hat{X}}_{R}),\\ \; & E\geq I(X_{H};X_{E})\\ \; & \mathrm{for}\;\mathrm{some}\;P_{X_{E},X_{E^{c}}}\cdot P_{{\hat{X}}_{R}|X_{E}}\}. \end{array}$$

We establish the next theorem. For the proof of this theorem, please refer to Section 3.3, Section 3.4 and Section 3.5.

**Theorem** **1.** *For any E such that R⊆E⊆K, the achievable region of the coding system is given by*

(20) $$\begin{array}{c} C_{E}\left(P_{X_{K}}\right)=S_{E}\left(P_{X_{K}}\right). \end{array}$$

To clarify the relationship with the conventional result of Shinohara and Yagi [3], we mention the achievable region among the coding rate, utility, and privacy, which is derived by projecting the result of Theorem 1 onto the R-D-L hyperplane.

**Definition** **4.** *For any E such that R⊆E⊆K, we define*

(21) $$\begin{array}{c} C_{E}^{RDL}\left(\epsilon |P_{X_{K}}\right)≔\left\{\left(R,D,L\right):\;\left(R,D,L,E\right)\in C_{E}\left(\epsilon |P_{X_{K}}\right)\right\} \end{array}$$

*and*

(22) $$\begin{array}{c} C_{E}^{RDL}\left(P_{X_{K}}\right)≔\underset{0<\epsilon <1}{⋂}C_{E}^{RDL}\left(\epsilon |P_{X_{K}}\right). \end{array}$$

**Definition** **5.** *For any E such that R⊆E⊆K, we define*

(23) $$\begin{array}{cc} S_{E}^{RDL}\left(P_{X_{K}}\right)=\{\left(R,D,L\right):\;\;\; & R\geq I(X_{E};{\hat{X}}_{R}),\\ \; & D\geq E[d\left(X_{R},{\hat{X}}_{R}\right)],\\ \; & L\geq I(X_{H};{\hat{X}}_{R})\\ \; & \mathrm{for}\;\mathrm{some}\;P_{X_{E},X_{E^{c}}}\cdot P_{{\hat{X}}_{R}|X_{E}}\}. \end{array}$$

**Corollary** **1.** *For any E such that R⊆E⊆K, the region $C_{E}^{RDL}\left(P_{X_{K}}\right)$ is given by*

(24) $$\begin{array}{c} C_{E}^{RDL}\left(P_{X_{K}}\right)=S_{E}^{RDL}\left(P_{X_{K}}\right). \end{array}$$

**Remark** **2.** *Corollary 1 suggests that the conventional result [3] can be obtained from $C_{E}\left(P_{X_{K}}\right)$.*

**Remark** **3.** *The derived characterization in (24) reduces to the characterization given in [1] when the encoded attribute E is either K or R. Thus, (24) gives its generalization for R⊆E⊆K.*

Examples to illustrate this result are shown in Section 6.1.

### 3.3. Proof Preliminaries for First-Order Rate Analysis

For preliminaries for coding theorems by the first-order rate analysis, we define strongly typical sequences that are necessary for the proof and show some properties. These proof preliminaries are also used in Section 4.

**Definition** **6** (Definition 2.1, [28])**.** *The type of a sequence $x^{n}\in X^{n}$ of length n is the distribution $P_{x^{n}}$ on X defined by*

(25) $$\begin{array}{c} P_{x^{n}}\left(a\right)≔\frac{1}{n}N\left(a|x^{n}\right), \end{array}$$

*where $N(a|x^{n})$ represents the number of occurrences of symbol a∈X in $x^{n}$. Likewise, the joint type of $x^{n}\in X^{n}$ and $y^{n}\in Y^{n}$ is the distribution $P_{x^{n}y^{n}}$ on X×Y defined by*

(26) $$\begin{array}{c} P_{x^{n}y^{n}}≔\frac{1}{n}N\left(a,b|x^{n},y^{n}\right), \end{array}$$

*where $N(a,b|x^{n},y^{n})$ represents the number of the occurrences of (a,b)∈X×Y in the pair of sequences $\left(x^{n},y^{n}\right)$.*

**Definition** **7** ((Conditional Type), [28], Definition 2.2)**.** *We define the conditional type of $y^{n}$ given $x^{n}$ as a stochastic matrix V:X→Y satisfying*

(27) $$\begin{array}{c} N\left(a,b|x^{n},y^{n}\right)=N\left(a|x^{n}\right)V\left(b|a\right). \end{array}$$

*In particular, the conditional type of $y^{n}$ given $x^{n}$ is uniquely determined and given by*

(28) $$\begin{array}{c} V\left(b|a\right)=\frac{N(a,b|x^{n},y^{n})}{N(a|x^{n})} \end{array}$$

*if $N(a|x^{n})>0$ for any a∈X.*

**Definition** **8** ((Strongly Typical Sequences), [29], Definition 1.2.8)**.** *For any distribution P on X, a sequence $x^{n}\in X^{n}$ is said to be P-typical with constant δ>0 if*

(29) $$\begin{array}{c} \left|\frac{1}{n}N\left(a|x^{n}\right)-P\left(a\right)\right|\leq \delta \;\;\;\;\mathrm{for}\;\mathrm{every}\;a\in X \end{array}$$

*and, in addition, no a∈X with P(a)=0 occurs in $x^{n}$. The set of such sequences is denoted by $T_{\delta }^{n}\left(P\right)$. If X is a random variable with values in X, we also refer to P-typical sequences as X-typical sequences and write $T_{\delta }^{n}\left(X\right)$.*

**Definition** **9** ((Conditional Strongly Typical Sequences), [29], Definition 1.2.9)**.** *For a stochastic matrix W:X→Y, a sequence $y^{n}\in Y^{n}$ is said to be W-typical given $x^{n}\in X^{n}$ with constant δ>0 if*

(30) $$\begin{array}{cc} \left|\frac{1}{n}N\left(a,b|x^{n},y^{n}\right)-\frac{1}{n}N\left(a|x^{n}\right)W\left(b|a\right)\right|\leq \delta & \\ \mathrm{for}\;\mathrm{every}\;a\in X,b\in Y, & \end{array}$$

*and, in addition, $N(a,b|x^{n},y^{n})=0$ whenever W(b|a)=0. The set of such sequences $y^{n}$ is denoted by $T_{\delta }^{n}\left(W|x^{n}\right)$. Further, if X and Y are random variables with values in X and Y, respectively, and $P_{Y|X}=W$, then they are also said to be Y|X-typical and written as $T_{\delta }^{n}\left(Y|X\right|x^{n})$.*

Hereafter, the set of conditional strongly typical sequences $T_{\delta }^{n}\left(Y|X\right|x^{n})$ is abbreviated as $T_{\delta }^{n}\left(Y|x^{n}\right)$.

We state some lemmas that are used in this proof.

**Lemma** **1** ([29], Lemma 1.2.13)**.** *For any positive sequences ${\left\{\delta_{n}\right\}}_{n=1}^{\infty }$ and ${\left\{\delta_{n}^{\prime }\right\}}_{n=1}^{\infty }$ such that $\delta_{n}\to 0$ and $\delta^{\prime }\to 0$ as n→0, there exists a sequence $\epsilon_{n}=\epsilon_{n}(|X,Y|,\delta_{n},\delta_{n}^{\prime })\to 0$(n→∞) such that for every distribution P on X and stochastic matrix W:X→Y,*

(31) $$\begin{array}{cc} \left|\frac{1}{n}\log|T_{\delta_{n}}^{n}\left(P\right)|-H\left(P\right)\right| & \leq \epsilon_{n}, \end{array}$$

(32) $$\begin{array}{cc} \left|\frac{1}{n}\log|T_{\delta_{n}^{\prime }}^{n}\left(W|x^{n}\right)|-H\left(W|P\right)\right| & \leq \epsilon_{n}. \end{array}$$

**Lemma** **2** ([29], Lemma 1.2.7)**.** *Let the variational distance between two distributions P and Q on X be defined as*

(33) $$\begin{array}{c} d_{v}\left(P,Q\right)≔\sum_{x\in X}\left|P\left(x\right)-Q\left(x\right)\right|. \end{array}$$

*If $d_{v}\left(P,Q\right)<\frac{1}{2}$, then*

(34) $$\begin{array}{c} \left|H\left(P\right)-H\left(Q\right)\right|\leq -d_{v}\left(P,Q\right)\cdot \log\frac{d_{v}\left(P,Q\right)}{\left|X\right|}. \end{array}$$

**Lemma** **3** ([29], Lemma 1.2.10)**.** *If $x^{n}\in T_{\delta }^{n}\left(X\right)$ and $y^{n}\in T_{\delta^{\prime }}^{n}\left(Y|x^{n}\right)$, then $\left(x^{n},y^{n}\right)\in T_{\delta +\delta^{\prime }}^{n}\left(X,Y\right)$ and, consequently, $y^{n}\in T_{\delta^{\prime \prime }}\left(Y\right)$ for $\delta^{\prime \prime }≔\left(\delta +\delta^{\prime }\right)\cdot \left|X\right|$.*

**Lemma** **4.** *If $\left(x^{n},y^{n}\right)\in T_{\delta }^{n}\left(X,Y\right)$, then $x^{n}\in T_{\delta_{1}}^{n}\left(X\right)$ and, consequently, $y^{n}\in T_{\delta_{2}}^{n}\left(Y|x^{n}\right)$ for $\delta_{1}≔\left|Y\right|\cdot \delta$ and $\delta_{2}≔\left(|Y|+1\right)\cdot \delta$.*

**Lemma** **5.** *If $y^{n}\in T_{\delta }^{n}\left(Y\right)$ and $\left(x^{n},y^{n}\right)\notin T_{2\delta }^{n}\left(X,Y\right)$, then $x^{n}\notin T_{\delta }^{n}\left(X|y^{n}\right)$.*

**Lemma** **6** ([29], Lemma 1.2.12 and Remark)**.** *For arbitrarily fixed δ>0 and every distribution P on X and stochastic matrix W:X→Y*

(35) $$\begin{array}{cc} \mathrm{Pr}\{X^{n}\in T_{\delta }^{n}\left(P\right)\} & \geq 1-2|X|e^{-2\delta^{2}n}, \end{array}$$

(36) $$\begin{array}{cc} \mathrm{Pr}\{Y^{n}\in T_{\delta }^{n}\left(W|x^{n}\right)|X^{n}=x^{n}\} & \geq 1-2|X|\cdot \left|Y\right|e^{-2\delta^{2}n}\\ \; & \;\mathrm{for}\;\mathrm{every}\;x^{n}\in X^{n}. \end{array}$$

### 3.4. Proof of Converse Part

In this part, we shall prove $C_{E}\left(P_{X_{K}}\right)\subseteq S_{E}\left(P_{X_{K}}\right)$.

Let a tuple $\left(R,D,L,E\right)\in C_{E}\left(P_{X_{K}}\right)$ be arbitrarily fixed. Then, there exists an $\left(n,2^{n(R+\epsilon )},D+\epsilon ,L+\epsilon ,E+\epsilon \right)$ code that satisfies (14)–(17). Let *Q* be a uniform random variable over {1,2,…,n} and let $p_{i}\left(x_{E,i},x_{E^{c},i},{\hat{x}}_{R,i}\right)$ be the conditional distribution given Q=i. Evaluating the inequalities for *R*, we obtain

(37) $$\begin{array}{cc} R+\epsilon & \overset{\left(a\right)}{\geq }\frac{1}{n}\log M_{n}\\ \; & \overset{\left(b\right)}{\geq }\frac{1}{n}H\left(J_{n}\right)\\ \; & \geq \frac{1}{n}I\left(J_{n};X_{E}^{n}\right)\\ \; & \overset{\left(c\right)}{=}\frac{1}{n}\left\{H\left(X_{E}^{n}\right)-H\left(X_{E}^{n}|J_{n},{\hat{X}}_{R}^{n}\right)\right\}\\ \; & \overset{\left(d\right)}{=}\frac{1}{n}\sum_{i=1}^{n}H\left(X_{E,i}\right)-\frac{1}{n}\sum_{i=1}^{n}H\left(X_{E,i}|X_{E}^{i-1},J_{n},{\hat{X}}_{R}^{n}\right)\\ \; & \overset{\left(e\right)}{\geq }\frac{1}{n}\sum_{i=1}^{n}H\left(X_{E,i}\right)-\frac{1}{n}\sum_{i=1}^{n}H\left(X_{E,i}|{\hat{X}}_{R,i}\right)\\ \; & \overset{\left(f\right)}{=}\sum_{i=1}^{n}\mathrm{Pr}\left\{Q=i\right\}H\left(X_{E,i}|Q=i\right)\\ \; & \;-\sum_{i=1}^{n}\mathrm{Pr}\left\{Q=i\right\}H\left(X_{E,i}|{\hat{X}}_{R,i},Q=i\right)\\ \; & =H\left(X_{E,Q}|Q\right)-H\left(X_{E,Q}|{\hat{X}}_{R,Q},Q\right)\\ \; & \overset{\left(g\right)}{=}H\left(X_{E}\right)-H\left(X_{E,Q}|{\hat{X}}_{R,Q},Q\right)\\ \; & \overset{\left(h\right)}{\geq }H\left(X_{E}\right)-H\left(X_{E}|{\hat{X}}_{R}\right)\\ \; & =I(X_{E};{\hat{X}}_{R}), \end{array}$$

(a) follows from (14),
(b) follows because $H\left(J_{n}\right)\leq \log|J_{n}|=\log M_{n}$,
(c) is due to the fact that ${\hat{X}}_{R}^{n}=g\left(J_{n}\right)$,
(d) follows because each $X_{K,i}$ is independent and ${\hat{X}}_{R}^{n}$ is a function of $J_{n}$,
(e) follows because conditioning reduces entropy,
(f) is due to the definition of *Q*,
(g) follows because $X_{E}⊥Q$, and
(h) follows because conditioning reduces entropy, where $\left(X_{E},{\hat{X}}_{R}\right)∼\sum_{i=1}^{n}\mathrm{Pr}\left\{Q=i\right\}p_{i}\left(x_{E,i},{\hat{x}}_{R,i}\right)=p\left(x_{E},{\hat{x}}_{R}\right)$.

Similarly, evaluating *D*, *L*, and *E*, respectively, we obtain

(38) $$\begin{array}{cc} D+\epsilon \; & \overset{\left(i\right)}{\geq }E\left[\frac{1}{n}\sum_{i=1}^{n}d\left(X_{R,i},{\hat{X}}_{R,i}\right)\right]\\ \; & =\frac{1}{n}\sum_{i=1}^{n}E\left[d\left(X_{R,i},{\hat{X}}_{R,i}\right)\right]\\ \; & \overset{\left(j\right)}{=}E_{Q}\left[E\left[d\left(X_{R,i},{\hat{X}}_{R,i}\right)|Q\right]\right]\\ \; & \overset{\left(k\right)}{=}E\left[d\left(X_{R},{\hat{X}}_{R}\right)\right], \end{array}$$

(39) $$\begin{array}{cc} L+\epsilon \; & \overset{\left(l\right)}{\geq }\frac{1}{n}I\left(X_{H}^{n};J_{n}\right)\\ \; & =\frac{1}{n}H\left(X_{H}^{n}\right)-\frac{1}{n}H\left(X_{H}^{n}|J_{n}\right)\\ \; & \overset{\left(m\right)}{=}H\left(X_{H}\right)-\frac{1}{n}\sum_{i=1}^{n}H\left(X_{H,i}|X_{H}^{i-1},J_{n}\right)\\ \; & \overset{\left(n\right)}{=}H\left(X_{H}\right)-\frac{1}{n}\sum_{i=1}^{n}H\left(X_{H,i}|X_{H}^{i-1},J_{n},{\hat{X}}_{R,i}\right)\\ \; & \overset{\left(o\right)}{\geq }H\left(X_{H}\right)-\frac{1}{n}\sum_{i=1}^{n}H\left(X_{H,i}|{\hat{X}}_{R,i}\right)\\ \; & \overset{\left(p\right)}{=}H\left(X_{H}\right)-\sum_{i=1}^{n}\mathrm{Pr}\left\{Q=i\right\}H\left(X_{H,i}|{\hat{X}}_{R,i},Q=i\right)\\ \; & =H\left(X_{H}\right)-H\left(X_{H,Q}|{\hat{X}}_{R,Q},Q\right)\\ \; & \overset{\left(q\right)}{\geq }H\left(X_{H}\right)-H\left(X_{H}|{\hat{X}}_{R}\right)\\ \; & =I(X_{H};{\hat{X}}_{R}), \end{array}$$

(40) $$\begin{array}{cc} E+\epsilon \; & \geq \frac{1}{n}I\left(X_{H}^{n};X_{E}^{n}\right)\\ \; & \overset{\left(r\right)}{=}\frac{1}{n}\sum_{i=1}^{n}I\left(X_{H,i};X_{E}^{n}|X_{H}^{i-1}\right)\\ \; & \overset{\left(s\right)}{=}\frac{1}{n}\sum_{i=1}^{n}I\left(X_{H,i};X_{E,i}\right)\\ \; & \overset{\left(t\right)}{=}I\left(X_{H};X_{E}\right), \end{array}$$

where

(i) is due to (15),
(j) is derived from the definition of *Q*,
(k) follows because $\left(X_{R},{\hat{X}}_{R}\right)∼\sum_{i=1}^{n}\mathrm{Pr}\left\{Q=i\right\}p_{i}\left(x_{R,i},{\hat{x}}_{R,i}\right)=p\left(x_{R},{\hat{x}}_{R}\right)$,
(l) is due to (16),
(m) follows because $i.i.d.\;P_{X_{K}^{n}}$,
(n) follows because ${\hat{X}}_{R}^{n}=g\left(J_{n}\right)$,
(o) follows from the fact that conditioning reduces entropy,
(p) is derived from the definition of *Q*, and
(q) follows because conditioning reduces entropy, where $\left(X_{H},{\hat{X}}_{R}\right)∼\sum_{i=1}^{n}\mathrm{Pr}\left\{Q=i\right\}p_{i}\left(x_{H,i},{\hat{x}}_{R,i}\right)=p\left(x_{H},{\hat{x}}_{R}\right)$,
(r) is due to chain rule for mutual information,
(s), (t) follow because $i.i.d.\;P_{X_{K}^{n}}$.

It is readily shown that the Markov chain $X_{E^{c}}$–$X_{E}$–${\hat{X}}_{R}$ holds (cf. Appendix A). We complete the proof of the converse part.

### 3.5. Proof of Direct Part

In this part, we provide a sketch of the proof of $S_{E}\left(P_{X_{K}}\right)\subseteq C_{E}\left(P_{X_{K}}\right)$.

Under an arbitrarily fixed distribution $P_{X_{E},X_{E^{c}}}\cdot P_{{\hat{X}}_{R}|X_{E}}$, any tuple $\left(R,D,L,E\right)\in S_{E}\left(P_{X_{K}}\right)$ is chosen such that

(41) $$\begin{array}{cc} R & >I(X_{E};{\hat{X}}_{R}), \end{array}$$

(42) $$\begin{array}{cc} D & >E[d\left(X_{R},{\hat{X}}_{R}\right)], \end{array}$$

(43) $$\begin{array}{cc} L & >I(X_{H};{\hat{X}}_{R}), \end{array}$$

(44) $$\begin{array}{cc} E & >I(X_{H};X_{E}). \end{array}$$

From (42) and (43), we can choose a sufficiently small ϵ>0 such that

(45) $$\begin{array}{cc} D & >E[d\left(X_{R},{\hat{X}}_{R}\right)]+\epsilon , \end{array}$$

(46) $$\begin{array}{cc} L & >I(X_{H};{\hat{X}}_{R})+\epsilon . \end{array}$$

In addition, with this ϵ, some constant $0<\tau <\frac{1}{2}$ is fixed such that

(47) $$\begin{array}{cc} \; & \tau \left(\log|X_{H}|+5\right)+4\tau \log\frac{|X_{H}|\cdot 2^{R}}{2\tau }<\epsilon . \end{array}$$

We can also choose positive numbers δ(≔δ(n)) such that

(48) $$\begin{array}{cc} \; & \left(\delta \left(n\right)+\delta_{1}\left(n\right)\right)|X_{R}\left|\cdot \right|{\hat{X}}_{R}|D_{\max}+\tau <\epsilon , \end{array}$$

(49) $$\begin{array}{cc} \; & 2\delta^{2}\left(n\right)\leq R-I\left(X_{E};{\hat{X}}_{R}\right)-\frac{1}{n}-\tau , \end{array}$$

(50) $$\begin{array}{cc} \; & \delta (n)\to 0, \end{array}$$

(51) $$\begin{array}{cc} \; & \sqrt{n}\cdot \delta \left(n\right)\to \infty \end{array}$$

as n→∞, where $\delta_{1}≔\left(\right|X_{E}\left|-\right|X_{R}\left|\right)\cdot \delta$ and $D_{\max}≔\max_{a\in X_{R},b\in {\hat{X}}_{R}}d\left(a,b\right)$. Let $\delta \left(n\right)=\frac{c}{\sqrt{n}}\log n$ where *c* is a constant, and obviously (50) and (51) are satisfied.

**Generation of codebook**: Randomly generate ${\hat{x}}_{R}^{n}\left(j\right)$ from the strongly typical sequences $T_{\delta }^{n}\left({\hat{X}}_{R}\right)$ for $j=1,2,\ldots ,M_{n}≔2^{nR}$. Reveal the codebook $C=\{{\hat{x}}_{R}^{n}\left(1\right),\ldots ,{\hat{x}}_{R}^{n}\left(M_{n}\right)\}$ to the encoder and decoder.

**Encoding**: If a sequence $x_{E}^{n}\in X_{E}^{n}$ satisfies $x_{K}^{n}=\left(x_{E}^{n},x_{E^{c}}^{n}\right)$ with some $x_{E^{c}}^{n}\in X_{E^{c}}^{n}$, we write $x_{E}^{n}≺x_{K}^{n}$. Given $x_{K}^{n}$, the encoder finds *j* such that $x_{E}^{n}\in T_{\delta }^{n}\left(X_{E}|{\hat{x}}_{R}\left(j\right)\right)$ and sets $f_{n}\left(x_{E}^{n}\right)=j$ where $T_{\delta }^{n}\left(X_{E}|{\hat{x}}_{R}\left(j\right)\right)$ is the conditional strongly typical sequences. If there exist multiple such *j*, $f_{n}\left(x_{E}^{n}\right)$ is set as the minimum one. If there are no such *j*, then $f_{n}\left(x_{E}^{n}\right)=M_{n}$.

**Decoding**: When *j* is observed, the decoder sets the reproduced sequence as ${\hat{X}}_{R}^{n}={\hat{x}}_{R}^{n}\left(j\right)$.

**Evaluation**: We define A(j), B(j), and $\tilde{A}\left(j\right)$ as

(52) $$\begin{array}{cc} A(j)≔ & \{x_{E}^{n}\;:\;f_{n}\left(x_{E}^{n}\right)=j\}, \end{array}$$

(53) $$\begin{array}{cc} B(j)≔ & \{x_{K}^{n}\;:\;x_{E}^{n}≺x_{K}^{n},f_{n}\left(x_{E}^{n}\right)=j\}, \end{array}$$

(54) $$\begin{array}{cc} \tilde{A}\left(j\right)≔ & \left\{\begin{array}{c} \left\{x_{K}^{n}\;:\;x_{E}^{n}≺x_{K}^{n},f_{n}\left(x_{E}^{n}\right)=j,x_{K}^{n}\in T_{2\delta }^{n}\left(X_{K}|{\hat{x}}_{R}^{n}\left(j\right)\right)\right\}\\ \;(j=1,2,\ldots ,M_{n}-1)\\ \left\{x_{K}^{n}\;:\;x_{K}^{n}\in X_{K}^{n}\setminus \overset{M_{n}-1}{\underset{j=1}{⋃}}\tilde{A}\left(j\right)\right\}\;\;\;\;\left(j=M_{n}\right). \end{array}\right. \end{array}$$

It is easily verified that A(j) for $j=1,2,\ldots ,M_{n}$ (also, B(j) and $\tilde{A}\left(j\right)$) is disjoint. From the definitions of $J_{n}$, A(j), and B(j),

(55) $$\begin{array}{cc} \mathrm{Pr}\left\{J_{n}=j\right\}=\mathrm{Pr}\left\{X_{E}^{n}\in A\left(j\right)\right\}=\mathrm{Pr}\left\{X_{K}^{n}\in B\left(j\right)\right\} & \\ \mathrm{for}\;j=1,2,\ldots ,M_{n}. & \end{array}$$

For sufficiently large *n*, we can prove (cf. Appendix B)

(56) $$\begin{array}{cc} |\mathrm{Pr}\left\{X_{K}^{n}\in B\left(j\right)\right\}-\mathrm{Pr}\left\{X_{K}^{n}\in \tilde{A}\left(j\right)\right\}\left|\leq 2\right|X_{K}\left|\cdot \right|{\hat{X}}_{R}|e^{-2\delta^{2}n} & \\ \mathrm{for}\;j=1,2,\ldots ,M_{n}-1. & \end{array}$$

For sufficiently large *n*, we can show that there exists a code $\left(f_{n},g_{n}\right)$ such that (cf. Appendix C)

(57) $$\begin{array}{cc} \; & r_{n}\leq R, \end{array}$$

(58) $$\begin{array}{cc} \; & u_{n}\leq E\left[d\left(X_{R},{\hat{X}}_{R}\right)\right]+\left(\delta +\delta_{1}\right)|X_{R}\left|\cdot \right|{\hat{X}}_{R}|D_{\max}+\tau , \end{array}$$

(59) $$\begin{array}{cc} \; & e_{n}\leq I\left(X_{H};X_{E}\right), \end{array}$$

(60) $$\begin{array}{cc} \; & \mathrm{Pr}\left\{X_{E}^{n}\notin \overset{M_{n}-1}{\underset{j=1}{⋃}}A\left(j\right)\right\}\leq \left(2\right|X_{E}\left|+1\right)e^{-2\delta^{2}n}, \end{array}$$

(61) $$\begin{array}{cc} \; & \mathrm{Pr}\left\{X_{K}^{n}\notin \overset{M_{n}-1}{\underset{j=1}{⋃}}\tilde{A}\left(j\right)\right\}\leq \tau , \end{array}$$

(62) $$\begin{array}{cc} \; & |\tilde{A}\left(j\right)|\geq 2^{n\{H\left(X_{K}|{\hat{X}}_{R}\right)-\tau \}}. \end{array}$$

For this code $\left(f_{n},g_{n}\right)$, we evaluate the privacy leakage against the decoder as

(63) $$\begin{array}{cc} l_{n}\; & ≔\frac{1}{n}I\left(X_{H}^{n};J_{n}\right)\\ \; & =\frac{1}{n}H\left(X_{H}^{n}\right)-\frac{1}{n}H\left(X_{H}^{n}|J_{n}\right)\\ \; & \overset{\left(a\right)}{=}H\left(X_{H}\right)-\frac{1}{n}\sum_{j=1}^{M_{n}}H\left(X_{H}^{n}|X_{K}^{n}\in B\left(j\right)\right)\mathrm{Pr}\left\{X_{K}^{n}\in B\left(j\right)\right\}\\ \; & \overset{\left(b\right)}{\leq }H\left(X_{H}\right)-\frac{1}{n}\sum_{j=1}^{M_{n}}H\left(X_{H}^{n}|X_{K}^{n}\in \tilde{A}\left(j\right)\right)\mathrm{Pr}\left\{X_{K}^{n}\in \tilde{A}\left(j\right)\right\}\\ \; & \;+4\tau \log\frac{|X_{H}|\cdot 2^{R}}{2\tau } \end{array}$$

(64) $$\begin{array}{cc} \; & \overset{\left(c\right)}{\leq }H\left(X_{H}\right)-\frac{1}{n}\sum_{j=1}^{M_{n}-1}H\left(X_{H}^{n}|X_{K}^{n}\in \tilde{A}\left(j\right)\right)\mathrm{Pr}\left\{X_{K}^{n}\in \tilde{A}\left(j\right)\right\}\\ \; & \;+4\tau \log\frac{|X_{H}|\cdot 2^{R}}{2\tau }\\ \; & =H\left(X_{H}\right)-\frac{1}{n}\sum_{j=1}^{M_{n}-1}[-\sum_{\begin{array}{c} x_{H}^{n} \end{array}}\mathrm{Pr}\left\{X_{H}^{n}=x_{H}^{n}|X_{K}^{n}\in \tilde{A}\left(j\right)\right\}\cdot \\ \; & \;\log\mathrm{Pr}\{X_{H}^{n}=x_{H}^{n}|X_{K}^{n}\in \tilde{A}\left(j\right)\}]\cdot \mathrm{Pr}\left\{X_{K}^{n}\in \tilde{A}\left(j\right)\right\}+4\tau \log\frac{|X_{H}|\cdot 2^{R}}{2\tau }, \end{array}$$

where

(a) follows because of $i.i.d.\;P_{X_{K}^{n}}$,
(b) is due to the inequality proved in Appendix D,
(c) follows by removing the term for $j=M_{n}$.

Here, for any $x_{H}^{n}$ satisfying $x_{K}^{n}=\left(x_{R}^{n},x_{H}^{n}\right)\in \tilde{A}\left(j\right)$ with some $x_{R}^{n}$, we can show that

(65) $$\begin{array}{cc} \; & \mathrm{Pr}\{X_{H}^{n}=x_{H}^{n}|X_{K}^{n}\in \tilde{A}\left(j\right)\}\\ \; & =\frac{\mathrm{Pr}\left\{X_{K}^{n}\in \tilde{A}\left(j\right)|X_{H}^{n}=x_{H}^{n}\right\}\mathrm{Pr}\left\{X_{H}^{n}=x_{H}^{n}\right\}}{\mathrm{Pr}\{X_{K}^{n}\in \tilde{A}\left(j\right)\}}\\ \; & =\frac{\sum_{x_{R}^{n}\;:\;\left(x_{R}^{n},x_{H}^{n}\right)\in \tilde{A}\left(j\right)}\mathrm{Pr}\left\{X_{R}^{n}=x_{R}^{n},X_{H}^{n}=x_{H}^{n}|X_{H}^{n}=x_{H}^{n}\right\}}{\sum_{\left({\tilde{x}}_{R}^{n},{\tilde{x}}_{H}^{n}\right)\in \tilde{A}\left(j\right)}\mathrm{Pr}\left\{X_{R}^{n}={\tilde{x}}_{R}^{n},X_{H}^{n}={\tilde{x}}_{H}^{n}\right\}}\cdot \mathrm{Pr}\left\{X_{H}^{n}=x_{H}^{n}\right\}\\ \; & \overset{\left(d\right)}{=}\frac{\sum_{x_{R}^{n}\;:\;\left(x_{R}^{n},x_{H}^{n}\right)\in \tilde{A}\left(j\right)}\mathrm{Pr}\left\{X_{R}^{n}=x_{R}^{n}|X_{H}^{n}=x_{H}^{n}\right\}}{\sum_{\left({\tilde{x}}_{R}^{n},{\tilde{x}}_{H}^{n}\right)\in \tilde{A}\left(j\right)}\mathrm{Pr}\left\{X_{R}^{n}={\tilde{x}}_{R}^{n},X_{H}^{n}={\tilde{x}}_{H}^{n}\right\}}\cdot \mathrm{Pr}\left\{X_{H}^{n}=x_{H}^{n}\right\}\\ \; & \overset{\left(e\right)}{\leq }\frac{\sum_{x_{R}^{n}\in T_{\delta_{3}}^{n}\left(X_{R}|x_{H}^{n},{\hat{x}}_{R}^{n}\left(j\right)\right)}\mathrm{Pr}\left\{X_{R}^{n}=x_{R}^{n}|X_{H}^{n}=x_{H}^{n}\right\}}{\sum_{\left({\tilde{x}}_{R}^{n},{\tilde{x}}_{H}^{n}\right)\in \tilde{A}\left(j\right)}\mathrm{Pr}\left\{X_{R}^{n}={\tilde{x}}_{R}^{n},X_{H}^{n}={\tilde{x}}_{H}^{n}\right\}}\cdot \mathrm{Pr}\left\{X_{H}^{n}=x_{H}^{n}\right\} \end{array}$$

(66) $$\begin{array}{cc} \; & \overset{\left(f\right)}{\leq }\frac{2^{n\{H\left(X_{R}|X_{H},{\hat{X}}_{R}\right)+\tau \}}\cdot 2^{-n\{H\left(X_{R}|X_{H}\right)-\tau \}}}{2^{n\{H\left(X_{K}|{\hat{X}}_{R}\right)-\tau \}}\cdot 2^{-n\{H\left(X_{K}\right)+\tau \}}}\cdot 2^{-n\{H\left(X_{H}\right)-\tau \}}\\ \; & =2^{-n\{H\left(X_{H}|{\hat{X}}_{R}\right)-5\tau \}}, \end{array}$$

where

(d) follows from the fact that
  $$\begin{array}{c} \mathrm{Pr}\left\{X_{R}^{n}=x_{R}^{n},X_{H}^{n}=x_{H}^{n}|X_{H}^{n}=x_{H}^{n}\right\}=\mathrm{Pr}\left\{X_{R}^{n}=x_{R}^{n}|X_{H}^{n}=x_{H}^{n}\right\}, \end{array}$$
(e) is due to the inequality proved in Appendix E, and
(f) follows because of the number of strongly typical sequences.

Therefore, from Equations (61), (64) and (66) we can obtain

(67) $$\begin{array}{cc} l_{n}\; & \leq H\left(X_{H}\right)-\frac{1}{n}\sum_{j=1}^{M_{n}-1}[n\sum_{\begin{array}{c} x_{H}^{n} \end{array}}\mathrm{Pr}\left\{X_{H}^{n}=x_{H}^{n}|X_{K}^{n}\in \tilde{A}\left(j\right)\right\}\cdot \\ \; & \;\left\{H\left(X_{H}|{\hat{X}}_{R}\right)-5\tau \right\}]\cdot \mathrm{Pr}\left\{X_{K}^{n}\in \tilde{A}\left(j\right)\right\}+4\tau \log\frac{|X_{H}|\cdot 2^{R}}{2\tau }\\ \; & =H\left(X_{H}\right)-\mathrm{Pr}\left\{X_{K}^{n}\in \left(\overset{M_{n}-1}{\underset{j=1}{⋃}}\tilde{A}\left(j\right)\right)\right\}\cdot \left\{H\left(X_{H}|{\hat{X}}_{R}\right)-5\tau \right\}+4\tau \log\frac{|X_{H}|\cdot 2^{R}}{2\tau }\\ \; & \leq H\left(X_{H}\right)-\left(1-\tau \right)\left\{H\left(X_{H}|{\hat{X}}_{R}\right)-5\tau \right\}+4\tau \log\frac{|X_{H}|\cdot 2^{R}}{2\tau }\\ \; & \leq I\left(X_{H};{\hat{X}}_{R}\right)+\tau (\log|X_{H}\left|+5\right)+4\tau \log\frac{|X_{H}|\cdot 2^{R}}{2\tau }. \end{array}$$

Since constants ϵ, τ, and δ are fixed to satisfy (45)–(48), from (44), (57)–(59) and (67), we obtain

(68) $$\begin{array}{cc} r_{n} & \leq R, \end{array}$$

(69) $$\begin{array}{cc} u_{n} & \leq E[d\left(X_{R},{\hat{X}}_{R}\right)]+\epsilon <D, \end{array}$$

(70) $$\begin{array}{cc} l_{n} & <I(X_{H};{\hat{X}}_{R})+\epsilon <L, \end{array}$$

(71) $$\begin{array}{cc} e_{n} & \leq I(X_{H};X_{E})<E. \end{array}$$

Therefore, for the fixed distribution $P_{X_{E},X_{E^{c}}}\cdot P_{{\hat{X}}_{R}|X_{E}}$ any tuple

(72) $$\begin{array}{cc} (R,D,L,E)\in \{(R,D,L,E):\;\;R & >I(X_{E};{\hat{X}}_{R}),\\ D & >E[d\left(X_{R},{\hat{X}}_{R}\right)],\\ L & >I(X_{H};{\hat{X}}_{R}),\\ E & >I\left(X_{H};X_{E}\right)\}≕S_{E}^{*}\left(P_{X_{K}}\right) \end{array}$$

is achievable. Consequently, $S_{E}^{*}\left(P_{X_{K}}\right)\subseteq C_{E}\left(P_{X_{K}}\right)$. Taking the closure for the left-hand side (l.h.s.), we obtain $Cl\left(S_{E}^{*}\left(P_{X_{K}}\right)\right)\subseteq C_{E}\left(P_{X_{K}}\right)$ because $C_{E}\left(P_{X_{K}}\right)$ is a closed set. We conclude that $S_{E}\left(P_{X_{K}}\right)={⋃}_{p}Cl\left(S_{E}^{*}\left(P_{X_{K}}\right)\right)\subseteq C_{E}\left(P_{X_{K}}\right)$ because the distribution $P_{X_{K}}=P_{X_{E},X_{E^{c}}}\cdot P_{{\hat{X}}_{R}|X_{E}}$ is fixed arbitrarily. We complete the proof of the direct part.

## 4. First-Order Rate Analysis with Excess-Distortion Probability

### 4.1. Performance Measures

Hereafter, let the pair of the encoder and decoder $\left(f_{n},g_{n}\right)$ be fixed.

For a given $M_{n}$, the coding rate is defined as

(73) $$\begin{array}{c} r_{n}≔\frac{1}{n}\log M_{n}. \end{array}$$

Let $d:\;X_{R}\times {\hat{X}}_{R}\to \left[0,\infty \right)$ be a distortion function between $x_{R}\in X_{R}$ and ${\hat{x}}_{R}\in {\hat{X}}_{R}$. The distortion between sequences $x_{R}^{n}\in X_{R}^{n}$ and ${\hat{x}}_{R}^{n}\in {\hat{X}}_{R}^{n}$ is defined as

(74) $$\begin{array}{cc} d(x_{R}^{n},{\hat{x}}_{R}^{n})≔ & \sum_{i=1}^{n}d\left(x_{R,i},{\hat{x}}_{R,i}\right). \end{array}$$

Then, the measure of utility is defined as

(75) $$\begin{array}{cc} u_{n}≔ & \mathrm{Pr}\left\{\frac{1}{n}d\left(X_{R}^{n},{\hat{X}}_{R}^{n}\right)>D\right\}. \end{array}$$

This measurement is called **excess-distortion probability** for D≥0.

In this system, the privacy of the hidden source sequence $X_{H}^{n}$ should be protected when the codeword $J_{n}$ is observed by decoder $g_{n}$. The measure of privacy for the decoder is defined as

(76) $$\begin{array}{c} l_{n}≔\frac{1}{n}I\left(X_{H}^{n};J_{n}\right), \end{array}$$

where $I(X_{H}^{n};J_{n})$ is the mutual information between $X_{H}^{n}$ and $J_{n}$.

The privacy of the hidden source sequence $X_{H}^{n}$ should be protected when the encoded information $X_{E}$ is observed by encoder $f_{n}$. The measurement of privacy for the encoder is defined as

(77) $$\begin{array}{c} e_{n}≔\frac{1}{n}I\left(X_{H}^{n};X_{E}^{n}\right), \end{array}$$

where $I(X_{H}^{n};X_{E}^{n})$ is the mutual information between $X_{H}^{n}$ and $X_{E}^{n}$.

### 4.2. Achievable Region and Theorem

We define the achievable region for the first-order rate analysis with the excess-distortion probability and state the obtained results.

**Definition** **10.** *A tuple (R,D,L,E) is said to be* ϵ***-achievable*** *(with respect to the excess-distortion probability) if, for any given ϵ>0, there exists a sequence of codes $\left(f_{n},g_{n}\right)$ satisfying*

(78) $$\begin{array}{cc} r_{n} & \leq R+\epsilon , \end{array}$$

(79) $$\begin{array}{cc} u_{n} & \leq \epsilon , \end{array}$$

(80) $$\begin{array}{cc} l_{n} & \leq L+\epsilon , \end{array}$$

(81) $$\begin{array}{cc} e_{n} & \leq E+\epsilon \end{array}$$

*for all sufficiently large n.*

The technical meanings of each constraint in Definition 10 can be interpreted as follows: Equation (78) evaluates how much the source sequence is compressed, so this rate should be decreased. Equation (79) is the constraint corresponding to the excess-distortion probability being less than ϵ, so this condition should also be decreased. Equation (80) constrains the amount of leaked private information to the decoder. Since private information should be kept secret for the receiver, this quantity should be decreased as well. Equation (81) constrains the amount of leaked private information to the encoder. For the same reason as (80), this quantity should also be decreased.

**Definition** **11.** *The closure of the set of ϵ-achievable tuples (R,D,L,E) is referred to as the* ϵ***-achievable region*** *and is denoted by $L_{E}\left(\epsilon |P_{X_{K}}\right)$ and define*

(82) $$\begin{array}{c} L_{E}\left(P_{X_{K}}\right)≔\underset{0<\epsilon <1}{⋂}L_{E}\left(\epsilon |P_{X_{K}}\right). \end{array}$$

We establish the following theorem. For the proof of this theorem, please refer to Section 4.3 and Section 4.4.

**Theorem** **2.** *For any E such that R⊆E⊆K, the achievable region of the coding system is given by*

(83) $$\begin{array}{c} L_{E}\left(P_{X_{K}}\right)=S_{E}\left(P_{X_{K}}\right). \end{array}$$

**Remark** **4.** *From Theorems 1 and 2, we find that the achievable region in which utility is measured by the expected distortion is equal to the one in which utility is measured by the excess-distortion probability.*

Because in Section 6 we discuss the achievable region among coding rate, utility, and privacy, a characterization of the achievable region is derived by projecting the characterization in Theorem 2 onto the R-D-L hyperplane.

**Definition** **12.** *For any E such that R⊆E⊆K, we define*

(84) $$\begin{array}{c} L_{E}^{RDL}\left(\epsilon |P_{X_{K}}\right)≔\left\{\left(R,D,L\right):\;\left(R,D,L,E\right)\in L_{E}\left(\epsilon |P_{X_{K}}\right)\right\} \end{array}$$

*and*

(85) $$\begin{array}{c} L_{E}^{RDL}\left(P_{X_{K}}\right)≔\underset{0<\epsilon <1}{⋂}L_{E}^{RDL}\left(\epsilon |P_{X_{K}}\right). \end{array}$$

**Definition** **13.** *For any E such that R⊆E⊆K, we define*

(86) $$\begin{array}{cc} S_{E}^{RDL}\left(P_{X_{K}}\right)=\{\left(R,D,L\right):\;\;\; & R\geq I(X_{E};{\hat{X}}_{R}),\\ \; & D\geq E[d\left(X_{R},{\hat{X}}_{R}\right)],\\ \; & L\geq I(X_{H};{\hat{X}}_{R})\\ \; & \mathrm{for}\;\mathrm{some}\;P_{X_{E},X_{E^{c}}}\cdot P_{{\hat{X}}_{R}|X_{E}}\}. \end{array}$$

**Corollary** **2.** *For any E such that R⊆E⊆K, the region $L_{E}^{RDL}\left(P_{X_{K}}\right)$ is given by*

(87) $$\begin{array}{c} L_{E}^{RDL}\left(P_{X_{K}}\right)=S_{E}^{RDL}\left(P_{X_{K}}\right). \end{array}$$

Examples of numerical calculation of this result are shown in Section 6.1.

Since we focus on the achievable region between utility and privacy in the next section, a characterization of the achievable region is derived by further projecting the result of Theorem 2 onto the D-L plane.

**Definition** **14.** *For any E such that R⊆E⊆K, we define*

(88) $$\begin{array}{c} L_{E}^{DL}\left(\epsilon |P_{X_{K}}\right)≔\left\{\left(D,L\right):\;\left(R,D,L,E\right)\in L_{E}\left(\epsilon |P_{X_{K}}\right)\right\} \end{array}$$

*and*

(89) $$\begin{array}{c} L_{E}^{DL}\left(P_{X_{K}}\right)≔\underset{0<\epsilon <1}{⋂}L_{E}^{DL}\left(\epsilon |P_{X_{K}}\right). \end{array}$$

**Definition** **15.** *For any E such that R⊆E⊆K, we define*

(90) $$\begin{array}{cc} S_{E}^{DL}\left(P_{X_{K}}\right)=\{\left(D,L\right):\;\;\; & D\geq E[d\left(X_{R},{\hat{X}}_{R}\right)],\\ \; & L\geq I(X_{H};{\hat{X}}_{R})\\ \; & \mathrm{for}\;\mathrm{some}\;P_{X_{E},X_{E^{c}}}\cdot P_{{\hat{X}}_{R}|X_{E}}\}. \end{array}$$

**Corollary** **3.** *For any E such that R⊆E⊆K, the region $L_{E}^{DL}\left(P_{X_{K}}\right)$ is given by*

(91) $$\begin{array}{c} L_{E}^{DL}\left(P_{X_{K}}\right)=S_{E}^{DL}\left(P_{X_{K}}\right). \end{array}$$

### 4.3. Proof of Converse Part

From Section 3.4 (proof of the converse part), we have

(92) $$\begin{array}{c} C_{E}\left(P_{X_{K}}\right)\subseteq S_{E}\left(P_{X_{K}}\right). \end{array}$$

Let a tuple $\left(R,D,L,E\right)\in L_{E}\left(P_{X_{K}}\right)$ be arbitrarily fixed and ϵ>0 and $\epsilon^{\prime }>0$ be given. From the argument of the method of types, the sequences $x_{R}^{n}$ are divided into two categories: distortion-typical or non-distortion-typical with some ${\hat{x}}_{R}^{n}$. The sequences of the former categories satisfy $\frac{1}{n}d\left(x_{R}^{n},{\hat{x}}_{R}^{n}\right)<D+\epsilon$ and the sequences of the latter one satisfy $\frac{1}{n}d\left(x_{R}^{n},{\hat{x}}_{R}^{n}\right)<d_{\max}$ where $d_{\max}≔\max_{x_{R}\in X_{R},\;{\hat{x}}_{R}\in {\hat{X}}_{R}}d\left(x_{R},{\hat{x}}_{R}\right)$. Then, the expected distortion is bounded from above as

(93) $$\begin{array}{cc} E\left[\frac{1}{n}d\left(X_{R}^{n},{\hat{X}}_{R}^{n}\right)\right]\; & \leq D+\epsilon +\mathrm{Pr}\left\{\frac{1}{n}d\left(x_{R}^{n},{\hat{x}}_{R}^{n}\right)>D+\epsilon \right\}\cdot d_{\max}\\ \; & \leq D+\epsilon +\mathrm{Pr}\left\{\frac{1}{n}d\left(x_{R}^{n},{\hat{x}}_{R}^{n}\right)>D\right\}\cdot d_{\max}\\ \; & \overset{\left(a\right)}{\leq }D+\epsilon +\epsilon^{\prime }d_{\max}, \end{array}$$

where (a) follows from (79) of ϵ-achievable in which utility is measured by the excess-distortion probability. Since $\epsilon +\epsilon^{\prime }d_{\max}$ can be arbitrarily small with proper choices of ϵ and $\epsilon^{\prime }$, (15) can be derived. This means

(94) $$\begin{array}{c} L_{E}\left(P_{X_{K}}\right)\subseteq C_{E}\left(P_{X_{K}}\right). \end{array}$$

From both inclusion relations,

(95) $$\begin{array}{c} L_{E}\left(P_{X_{K}}\right)\subseteq C_{E}\left(P_{X_{K}}\right)\subseteq S_{E}\left(P_{X_{K}}\right) \end{array}$$

is evidently satisfied.

### 4.4. Proof of the Direct Part

In this part, we provide a sketch of the proof of $S_{E}\left(P_{X_{K}}\right)\subseteq L_{E}\left(\epsilon |P_{X_{K}}\right)$.

Under an arbitrarily fixed distribution $P_{X_{E},X_{E^{c}}}\cdot P_{{\hat{X}}_{R}|X_{E}}$, any tuple $\left(R,D,L,E\right)\in S_{E}\left(P_{X_{K}}\right)$ is chosen such that

(96) $$\begin{array}{cc} R & >I(X_{E};{\hat{X}}_{R}), \end{array}$$

(97) $$\begin{array}{cc} D & >E[d\left(X_{R},{\hat{X}}_{R}\right)], \end{array}$$

(98) $$\begin{array}{cc} L & >I(X_{H};{\hat{X}}_{R}), \end{array}$$

(99) $$\begin{array}{cc} E & >I(X_{H};X_{E}). \end{array}$$

From (97) and (98) , we can choose a sufficiently small ϵ>0 such that

(100) $$\begin{array}{cc} D & >E[d\left(X_{R},{\hat{X}}_{R}\right)]+\epsilon , \end{array}$$

(101) $$\begin{array}{cc} L & >I(X_{H};{\hat{X}}_{R})+\epsilon . \end{array}$$

In addition, with this ϵ, some constant $0<\tau <\frac{1}{2}$ is fixed such that

(102) $$\begin{array}{cc} \; & \tau \left(\log|X_{H}|+5\right)+4\tau \log\frac{|X_{H}|\cdot 2^{R}}{2\tau }<\epsilon . \end{array}$$

We can also choose positive numbers δ(≔δ(n)) such that

(103) $$\begin{array}{cc} \; & 2\delta^{2}\left(n\right)\leq R-I\left(X_{E};{\hat{X}}_{R}\right)-\frac{1}{n}-\tau , \end{array}$$

(104) $$\begin{array}{cc} \; & \delta (n)\to 0, \end{array}$$

(105) $$\begin{array}{cc} \; & \sqrt{n}\cdot \delta \left(n\right)\to \infty \end{array}$$

as n→∞. Let $\delta \left(n\right)=\frac{c}{\sqrt{n}}\log n$ where *c* is a constant, and obviously (104) and (105) are satisfied.

**Generation of codebook**: Randomly generate ${\hat{x}}_{R}^{n}\left(j\right)$ from the strongly typical sequences $T_{\delta }^{n}\left({\hat{X}}_{R}\right)$ for $j=1,2,\ldots ,M_{n}≔2^{nR}$. Reveal the codebook $C=\{{\hat{x}}_{R}^{n}\left(1\right),\ldots ,{\hat{x}}_{R}^{n}\left(M_{n}\right)\}$ to the encoder and decoder.

**Encoding**: If a sequence $x_{E}^{n}\in X_{E}^{n}$ satisfies $x_{K}^{n}=\left(x_{E}^{n},x_{E^{c}}^{n}\right)$ with some $x_{E^{c}}^{n}\in X_{E^{c}}^{n}$, we write $x_{E}^{n}≺x_{K}^{n}$. Given $x_{K}^{n}$, the encoder finds *j* such that $x_{E}^{n}\in T_{\delta }^{n}\left(X_{E}|{\hat{x}}_{R}^{n}\left(j\right)\right)$ and sets $f_{n}\left(x_{E}^{n}\right)=j$ where $T_{\delta }^{n}\left(X_{E}|{\hat{x}}_{R}^{n}\left(j\right)\right)$ is the conditional strongly typical sequences. If there exist multiple such *j*, $f_{n}\left(x_{E}^{n}\right)$ is set as the minimum one. If there are no such *j*, then $f_{n}\left(x_{E}^{n}\right)=M_{n}$.

**Decoding**: When *j* is observed, the decoder sets the reproduced sequence as ${\hat{X}}_{R}^{n}={\hat{x}}_{R}^{n}\left(j\right)$.

**Evaluation**: We define A(j), B(j), and $\tilde{A}\left(j\right)$ as

(106) $$\begin{array}{cc} A(j)≔ & \{x_{E}^{n}\;:\;f_{n}\left(x_{E}^{n}\right)=j\}, \end{array}$$

(107) $$\begin{array}{cc} B(j)≔ & \{x_{K}^{n}\;:\;x_{E}^{n}≺x_{K}^{n},f_{n}\left(x_{E}^{n}\right)=j\}, \end{array}$$

(108) $$\begin{array}{cc} \tilde{A}\left(j\right)≔ & \left\{\begin{array}{c} \left\{x_{K}^{n}\;:\;x_{E}^{n}≺x_{K}^{n},f_{n}\left(x_{E}^{n}\right)=j,x_{K}^{n}\in T_{2\delta }^{n}\left(X_{K}|{\hat{x}}_{R}^{n}\left(j\right)\right)\right\}\\ \;(j=1,2,\ldots ,M_{n}-1)\\ \left\{x_{K}^{n}\;:\;x_{K}^{n}\in X_{K}^{n}\setminus \overset{M_{n}-1}{\underset{j=1}{⋃}}\tilde{A}\left(j\right)\right\}\;\;\;\;\left(j=M_{n}\right). \end{array}\right. \end{array}$$

It is easily verified that A(j) for $j=1,2,\ldots ,M_{n}$ (and also B(j) and $\tilde{A}\left(j\right)$) is disjoint. From the definitions of $J_{n}$, A(j), and B(j),

(109) $$\begin{array}{cc} \mathrm{Pr}\left\{J_{n}=j\right\}=\mathrm{Pr}\left\{X_{E}^{n}\in A\left(j\right)\right\}=\mathrm{Pr}\left\{X_{K}^{n}\in B\left(j\right)\right\} & \\ \mathrm{for}\;j=1,2,\ldots ,M_{n}. & \end{array}$$

For sufficiently large *n*, we can prove (cf. Appendix B)

(110) $$\begin{array}{cc} |\mathrm{Pr}\left\{X_{K}^{n}\in B\left(j\right)\right\}-\mathrm{Pr}\left\{X_{K}^{n}\in \tilde{A}\left(j\right)\right\}\left|\leq 2\right|X_{K}\left|\cdot \right|{\hat{X}}_{R}|e^{-2\delta^{2}n} & \\ \mathrm{for}\;j=1,2,\ldots ,M_{n}-1. & \end{array}$$

For sufficiently large *n*, we can show that there exists a code $\left(f_{n},g_{n}\right)$ such that (cf. Appendix F)

(111) $$\begin{array}{cc} \; & r_{n}\leq R, \end{array}$$

(112) $$\begin{array}{cc} \; & \mathrm{Pr}\left\{X_{E}^{n}\notin \overset{M_{n}-1}{\underset{j=1}{⋃}}A\left(j\right)\right\}\leq \left(2\right|X_{E}\left|+1\right)e^{-2\delta^{2}n}, \end{array}$$

(113) $$\begin{array}{cc} \; & u_{n}\leq \left(2\right|X_{E}\left|+1\right)e^{-2\delta^{2}n}, \end{array}$$

(114) $$\begin{array}{cc} \; & e_{n}\leq I\left(X_{H};X_{E}\right), \end{array}$$

(115) $$\begin{array}{cc} \; & \mathrm{Pr}\left\{X_{K}^{n}\notin \overset{M_{n}-1}{\underset{j=1}{⋃}}\tilde{A}\left(j\right)\right\}\leq \tau , \end{array}$$

(116) $$\begin{array}{cc} \; & |\tilde{A}\left(j\right)|\geq 2^{n\{H\left(X_{K}|{\hat{X}}_{R}\right)-\tau \}}. \end{array}$$

For this code $\left(f_{n},g_{n}\right)$, we evaluate the privacy leakage against the decoder as

(117) $$\begin{array}{cc} l_{n} & ≔\frac{1}{n}I\left(X_{H}^{n};J_{n}\right)\\ \; & =\frac{1}{n}H\left(X_{H}^{n}\right)-\frac{1}{n}H\left(X_{H}^{n}|J_{n}\right)\\ \; & \overset{\left(a\right)}{=}H\left(X_{H}\right)-\frac{1}{n}H\left(X_{H}^{n}|J_{n}\right)\\ \; & =H\left(X_{H}\right)-\frac{1}{n}\sum_{j=1}^{M_{n}}H\left(X_{H}^{n}|X_{K}^{n}\in B\left(j\right)\right)\mathrm{Pr}\left\{X_{K}^{n}\in B\left(j\right)\right\}\\ \; & \overset{\left(b\right)}{\leq }H\left(X_{H}\right)-\frac{1}{n}\sum_{j=1}^{M_{n}}H\left(X_{H}^{n}|X_{K}^{n}\in \tilde{A}\left(j\right)\right)\mathrm{Pr}\left\{X_{K}^{n}\in \tilde{A}\left(j\right)\right\} \end{array}$$

(118) $$\begin{array}{cc} \; & \;+4\tau \log\frac{|X_{H}|\cdot 2^{R}}{2\tau }\\ \; & \overset{\left(c\right)}{\leq }H\left(X_{H}\right)-\frac{1}{n}\sum_{j=1}^{M_{n}-1}H\left(X_{H}^{n}|X_{K}^{n}\in \tilde{A}\left(j\right)\right)\mathrm{Pr}\left\{X_{K}^{n}\in \tilde{A}\left(j\right)\right\}\\ \; & \;+4\tau \log\frac{|X_{H}|\cdot 2^{R}}{2\tau }\\ \; & =H\left(X_{H}\right)-\frac{1}{n}\sum_{j=1}^{M_{n}-1}[-\sum_{\begin{array}{c} x_{H}^{n} \end{array}}\mathrm{Pr}\left\{X_{H}^{n}=x_{H}^{n}|X_{K}^{n}\in \tilde{A}\left(j\right)\right\}\cdot \log\mathrm{Pr}\{X_{H}^{n}=x_{H}^{n}|X_{K}^{n}\in \tilde{A}\left(j\right)\}]\cdot \end{array}$$

(119) $$\begin{array}{cc} \; & \;\mathrm{Pr}\left\{X_{K}^{n}\in \tilde{A}\left(j\right)\right\}+4\tau \log\frac{|X_{H}|\cdot 2^{R}}{2\tau }, \end{array}$$

where

(a) follows because of $i.i.d.\;P_{X_{K}^{n}}$,
(b) is due to the inequality proved in Appendix D, and
(c) follows by removing the term for $j=M_{n}$.

Here, for any $x_{H}^{n}$ satisfying $x_{K}^{n}=\left(x_{R}^{n},x_{H}^{n}\right)\in \tilde{A}\left(j\right)$ with some $x_{R}^{n}$, we can show that

(120) $$\begin{array}{cc} \; & \mathrm{Pr}\{X_{H}^{n}=x_{H}^{n}|X_{K}^{n}\in \tilde{A}\left(j\right)\}\\ \; & =\frac{\mathrm{Pr}\left\{X_{K}^{n}\in \tilde{A}\left(j\right)|X_{H}^{n}=x_{H}^{n}\right\}\mathrm{Pr}\left\{X_{H}^{n}=x_{H}^{n}\right\}}{\mathrm{Pr}\{X_{K}^{n}\in \tilde{A}\left(j\right)\}}\\ \; & =\frac{\sum_{x_{R}^{n}\;:\;\left(x_{R}^{n},x_{H}^{n}\right)\in \tilde{A}\left(j\right)}\mathrm{Pr}\left\{X_{R}^{n}=x_{R}^{n},X_{H}^{n}=x_{H}^{n}|X_{H}^{n}=x_{H}^{n}\right\}}{\sum_{\left({\tilde{x}}_{R}^{n},{\tilde{x}}_{H}^{n}\right)\in \tilde{A}\left(j\right)}\mathrm{Pr}\left\{X_{R}^{n}={\tilde{x}}_{R}^{n},X_{H}^{n}={\tilde{x}}_{H}^{n}\right\}}\cdot \mathrm{Pr}\left\{X_{H}^{n}=x_{H}^{n}\right\}\\ \; & \overset{\left(d\right)}{=}\frac{\sum_{x_{R}^{n}\;:\;\left(x_{R}^{n},x_{H}^{n}\right)\in \tilde{A}\left(j\right)}\mathrm{Pr}\left\{X_{R}^{n}=x_{R}^{n}|X_{H}^{n}=x_{H}^{n}\right\}}{\sum_{\left({\tilde{x}}_{R}^{n},{\tilde{x}}_{H}^{n}\right)\in \tilde{A}\left(j\right)}\mathrm{Pr}\left\{X_{R}^{n}={\tilde{x}}_{R}^{n},X_{H}^{n}={\tilde{x}}_{H}^{n}\right\}}\cdot \mathrm{Pr}\left\{X_{H}^{n}=x_{H}^{n}\right\}\\ \; & \overset{\left(e\right)}{\leq }\frac{\sum_{x_{R}^{n}\in T_{\delta_{3}}^{n}\left(X_{R}|x_{H}^{n},{\hat{x}}_{R}^{n}\left(j\right)\right)}\mathrm{Pr}\left\{X_{R}^{n}=x_{R}^{n}|X_{H}^{n}=x_{H}^{n}\right\}}{\sum_{\left({\tilde{x}}_{R}^{n},{\tilde{x}}_{H}^{n}\right)\in \tilde{A}\left(j\right)}\mathrm{Pr}\left\{X_{R}^{n}={\tilde{x}}_{R}^{n},X_{H}^{n}={\tilde{x}}_{H}^{n}\right\}}\cdot \mathrm{Pr}\left\{X_{H}^{n}=x_{H}^{n}\right\} \end{array}$$

(121) $$\begin{array}{cc} \; & \overset{\left(f\right)}{\leq }\frac{2^{n\{H\left(X_{R}|X_{H},{\hat{X}}_{R}\right)+\tau \}}\cdot 2^{-n\{H\left(X_{R}|X_{H}\right)-\tau \}}}{2^{n\{H\left(X_{K}|{\hat{X}}_{R}\right)-\tau \}}\cdot 2^{-n\{H\left(X_{K}\right)+\tau \}}}\cdot 2^{-n\{H\left(X_{H}\right)-\tau \}}\\ \; & =2^{-n\{H\left(X_{H}|{\hat{X}}_{R}\right)-5\tau \}}, \end{array}$$

where

(d) follows from the fact that
  $$\begin{array}{c} \mathrm{Pr}\left\{X_{R}^{n}=x_{R}^{n},X_{H}^{n}=x_{H}^{n}|X_{H}^{n}=x_{H}^{n}\right\}=\mathrm{Pr}\left\{X_{R}^{n}=x_{R}^{n}|X_{H}^{n}=x_{H}^{n}\right\}, \end{array}$$
(e) is due to the inequality proved in Appendix E, and
(f) follows because of the number of strongly typical sequences.

Therefore, from Equations (115), (119), and (121), we can obtain

(122) $$\begin{array}{cc} l_{n}\; & \leq H\left(X_{H}\right)-\frac{1}{n}\sum_{j=1}^{M_{n}-1}[n\sum_{\begin{array}{c} x_{H}^{n} \end{array}}\mathrm{Pr}\left\{X_{H}^{n}=x_{H}^{n}|X_{K}^{n}\in \tilde{A}\left(j\right)\right\}\cdot \\ \; & \;\left\{H\left(X_{H}|{\hat{X}}_{R}\right)-5\tau \right\}]\cdot \mathrm{Pr}\left\{X_{K}^{n}\in \tilde{A}\left(j\right)\right\}+4\tau \log\frac{|X_{H}|\cdot 2^{R}}{2\tau }\\ \; & =H\left(X_{H}\right)-\mathrm{Pr}\left\{X_{K}^{n}\in \left(\overset{M_{n}-1}{\underset{j=1}{⋃}}\tilde{A}\left(j\right)\right)\right\}\cdot \left\{H\left(X_{H}|{\hat{X}}_{R}\right)-5\tau \right\}+4\tau \log\frac{|X_{H}|\cdot 2^{R}}{2\tau }\\ \; & \leq H\left(X_{H}\right)-\left(1-\tau \right)\left\{H\left(X_{H}|{\hat{X}}_{R}\right)-5\tau \right\}+4\tau \log\frac{|X_{H}|\cdot 2^{R}}{2\tau }\\ \; & \leq I\left(X_{H};{\hat{X}}_{R}\right)+\tau \left\{H\left(X_{H}|{\hat{X}}_{R}\right)+5\right\}+4\tau \log\frac{|X_{H}|\cdot 2^{R}}{2\tau }. \end{array}$$

Since constants ϵ, τ, and δ are fixed to satisfy (100)–(102), from (111), (113), and (122), we obtain

(123) $$\begin{array}{cc} \; & r_{n}\leq R, \end{array}$$

(124) $$\begin{array}{cc} \; & u_{n}\leq \epsilon , \end{array}$$

(125) $$\begin{array}{cc} \; & l_{n}<I\left(X_{H};{\hat{X}}_{R}\right)+\epsilon <L, \end{array}$$

(126) $$\begin{array}{cc} \; & e_{n}\leq I\left(X_{H};X_{E}\right)<E. \end{array}$$

Therefore, for the fixed distribution $P_{X_{E},X_{E^{c}}}\cdot P_{{\hat{X}}_{R}|X_{E}}$, any tuple

(127) $$\begin{array}{cc} (R,D,L,E)\in \{(R,D,L,E):\;\;R & >I(X_{E};{\hat{X}}_{R}),\\ D & >E[d\left(X_{R},{\hat{X}}_{R}\right)],\\ L & >I(X_{H};{\hat{X}}_{R}),\\ E & >I\left(X_{H};X_{E}\right)\}≕S_{E}^{*}\left(P_{X_{K}}\right) \end{array}$$

is achievable. Consequently, $S_{E}^{*}\left(P_{X_{K}}\right)\subseteq L_{E}\left(\epsilon |P_{X_{K}}\right)$. Taking the closure for the l.h.s., we obtain $Cl\left(S_{E}^{*}\left(P_{X_{K}}\right)\right)\subseteq L_{E}\left(\epsilon |P_{X_{K}}\right)$ because $L_{E}\left(\epsilon |P_{X_{K}}\right)$ is a closed set. We conclude that $S_{E}\left(P_{X_{K}}\right)={⋃}_{p}Cl\left(S_{E}^{*}\left(P_{X_{K}}\right)\right)\subseteq L_{E}\left(\epsilon |P_{X_{K}}\right)$ because the distribution $P_{X_{K},{\hat{X}}_{R}}=P_{X_{E},X_{E^{c}}}\cdot P_{{\hat{X}}_{R}|X_{E}}$ is fixed arbitrarily. We complete the proof of the direct part.

## 5. Strong Converse Theorem for Utility–Privacy Trade-Offs

### 5.1. Another Expression of the Achievable Region

In Section 5.1, we clarify that the achievable region $L_{E}^{DL}\left(P_{X_{K}}\right)$ defined in (89) coincides with the region expressed with a tangent plane.

**Definition** **16.** *For any E such that R⊆E⊆K, the region $T_{E}^{\mu }\left(P_{X_{K}}\right)$ is defined as*

$$\begin{array}{cc} \; & T_{E}^{\mu }\left(P_{X_{K}}\right)≔\min\left\{I\left(X_{H};{\hat{X}}_{R}\right)+\mu E\left[d\left(X_{R},{\hat{X}}_{R}\right)\right]\;\mathrm{for}\;\mathrm{some}\;P_{{\hat{X}}_{R}|X_{E}}\cdot P_{X_{E^{c}}X_{E}}\right\},\\ \; & \;where\\ \; & T_{E}^{DL}\left(P_{X_{K}}\right)≔\underset{\mu \geq 0}{⋂}\left\{\left(L,D\right):\;L+\mu D\geq T_{E}^{\mu }\left(P_{X_{K}}\right)\right\}. \end{array}$$

**Theorem** **3.** *For any E such that R⊆E⊆K, the region $S_{E}^{DL}\left(P_{X_{K}}\right)$ defined in (90) is given by*

(128) $$\begin{array}{c} S_{E}^{DL}\left(P_{X_{K}}\right)=T_{E}^{DL}\left(P_{X_{K}}\right), \end{array}$$

*and the achievable region $L_{E}^{DL}\left(P_{X_{K}}\right)$, which is the projection region of the achievable region $L_{E}\left(P_{X_{K}}\right)$ onto the D-L plane, is given by*

(129) $$\begin{array}{c} L_{E}^{DL}\left(P_{X_{K}}\right)=T_{E}^{DL}\left(P_{X_{K}}\right). \end{array}$$

**Figure 3:**
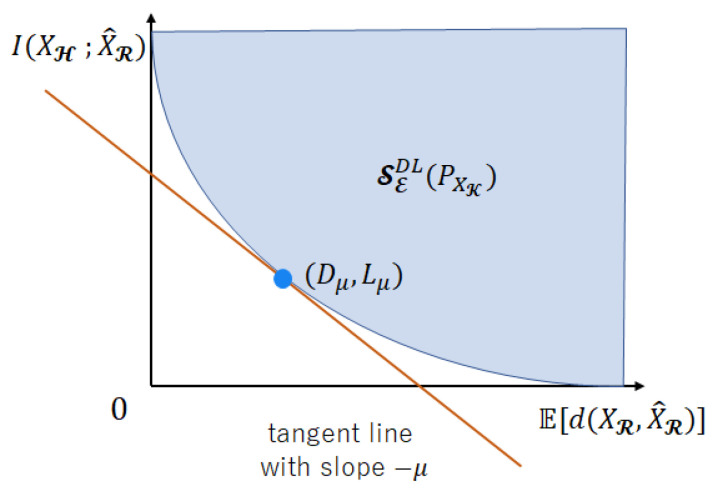
The region expressed with a tangent plane using the Legendre transformation.

**Proof.**  Figure 3 illustrates the proof image using a graph. Let a constance μ≥0 be fixed arbitrarily. Like in Figure 3, there exists a boundary point $\left(D_{\mu },L_{\mu }\right)$ of $S_{E}^{DL}$ tangent to the line with slope −μ. The intercept of this tangent line is $L_{\mu }+\mu D_{\mu }$.

The minimum $I\left(X_{H};{\hat{X}}_{R}\right)+\mu E\left[d\left(X_{R},{\hat{X}}_{R}\right)\right]$ characterized by some distribution $P_{{\hat{X}}_{R}|X_{E}}$ coincides with $L_{\mu }+\mu D_{\mu }$. Therefore,

(130) $$\begin{array}{cc} L_{\mu }+\mu D_{\mu }=\min & \{I\left(X_{H};{\hat{X}}_{R}\right)+\mu E\left[d\left(X_{R},{\hat{X}}_{R}\right)\right]\;\mathrm{for}\;\mathrm{some}\;P_{{\hat{X}}_{R}|X_{E}}\cdot P_{X_{E^{c}}X_{E}}\}. \end{array}$$

From (130), we obtain

(131) $$\begin{array}{cc} \; & \left\{\left(L,D\right):\;L+\mu D\geq L_{\mu }+\mu D_{\mu }\right\}=\{\left(L,D\right):\;L+\mu D\geq \min\{I\left(X_{H};{\hat{X}}_{R}\right)\\ \; & \;+\mu E\left[d\left(X_{R},{\hat{X}}_{R}\right)\right]\;\mathrm{for}\;\mathrm{some}\;P_{{\hat{X}}_{R}|X_{E}}\cdot P_{X_{E^{c}}X_{E}}\}\}. \end{array}$$

Taking the intersection by μ≥0 on the both sides of (131),

(132) $$\begin{array}{cc} \; & \underset{\mu \geq 0}{⋂}\left\{\left(L,D\right):\;L+\mu D\geq L_{\mu }+\mu D_{\mu }\right\}=\underset{\mu \geq 0}{⋂}\left\{\left(L,D\right):\;L+\mu D\geq T_{E}^{\mu }\left(P_{X_{K}}\right)\right\}. \end{array}$$

The l.h.s. of (131) shows the upper-right region in the first quadrant drawn by the tangent line with a slope −μ for $S_{E}^{DL}\left(P_{X_{K}}\right)$. Since the l.h.s. of (132) is the intersection of the l.h.s. of (131), the l.h.s. of (132) represents $S_{E}^{DL}\left(P_{X_{K}}\right)$. From Definition 16, the right-hand side (r.h.s.) of (132) is $T_{E}^{DL}\left(P_{X_{K}}\right)$. As a result, (128) holds. Since $L_{E}^{DL}\left(P_{X_{K}}\right)=S_{E}^{DL}\left(P_{X_{K}}\right)$ from Corollary 3, likewise, (129) holds. □

### 5.2. Proof Preliminaries

In Section 5.2, we derive two fundamental properties of the minimization about two values and the inequalities about entropy and divergence to prove the strong converse theorem. In Proposition 1, we change the objective function $T_{E}^{\mu }\left(P_{X_{K}}\right)$ of the region expressed with the tangent plane introduced in Section 5.1 onto the region expressed with divergence.

**Proposition** **1.** *Let μ≤0 be fixed arbitrarily. For any E such that R⊆E⊆K,*

(133) $$\begin{array}{cc} T_{E}^{\mu }\left(P_{X_{K}}\right) & =\underset{\alpha >0}{\sup}T_{E}^{\mu ,\alpha }\left(P_{X_{K}}\right), \end{array}$$

*where*

(134) $$\begin{array}{cc} T_{E}^{\mu ,\alpha }\left(P_{X_{K}}\right)≔\; & \min_{P_{{\tilde{X}}_{E^{c}}{\tilde{X}}_{E}{\tilde{\hat{X}}}_{R}}}[I\left({\tilde{X}}_{H};{\tilde{\hat{X}}}_{R}\right)+\mu E\left[d\left({\tilde{X}}_{R},{\tilde{\hat{X}}}_{R}\right)\right]\\ \; & \;+\alpha D\left(P_{{\tilde{X}}_{E^{c}}{\tilde{X}}_{E}{\tilde{\hat{X}}}_{R}}\|Q_{X_{E^{c}}X_{E}{\tilde{\hat{X}}}_{R}}\right)+D\left(P_{{\tilde{X}}_{E^{c}}{\tilde{X}}_{E}}\|P_{X_{E^{c}}X_{E}}\right)]\\ \; & \;=\min_{P_{{\tilde{X}}_{E^{c}}{\tilde{X}}_{E}{\tilde{\hat{X}}}_{R}}}\left[I\left({\tilde{X}}_{H};{\tilde{\hat{X}}}_{R}\right)+\mu E\left[d\left({\tilde{X}}_{R},{\tilde{\hat{X}}}_{R}\right)\right]\right]\\ \; & \;+\left(\alpha +1\right)D\left(P_{{\tilde{X}}_{E^{c}}{\tilde{X}}_{E}}\|P_{X_{E^{c}}X_{E}}\right)+\alpha I\left({\tilde{X}}_{E^{c}};{\tilde{\hat{X}}}_{R}|{\tilde{X}}_{E}\right)], \end{array}$$

*and $Q_{X_{E^{c}}X_{E}{\tilde{\hat{X}}}_{R}}$ is the distribution induced from each $P_{{\tilde{X}}_{E^{c}}{\tilde{X}}_{E}{\tilde{\hat{X}}}_{R}}$.*

**Proof.**  First, it is clear that $T_{E}^{\mu }\left(P_{X_{K}}\right)\geq T_{E}^{\mu ,\alpha }\left(P_{X_{K}}\right)$ for all α>0. To prove $T_{E}^{\mu }\left(P_{X_{K}}\right)\leq T_{E}^{\mu ,\alpha }\left(P_{X_{K}}\right)$ for some α>0, for α>0, let $P_{{\tilde{X}}_{E^{c}}{\tilde{X}}_{E}{\tilde{\hat{X}}}_{R}}^{\alpha }$ be the distribution that minimizes the r.h.s. of (134) and $Q_{X_{E^{c}}X_{E}{\tilde{\hat{X}}}_{R}}^{\alpha }=P_{{\tilde{\hat{X}}}_{R}|{\tilde{X}}_{E}}P_{X_{E^{c}}X_{E}}$ be the estimated distribution. Since $G\left(P_{{\tilde{X}}_{E^{c}}{\tilde{X}}_{E}{\tilde{\hat{X}}}_{R}}^{\alpha }\right)I≔\left({\tilde{X}}_{H};{\tilde{\hat{X}}}_{R}\right)+E\left[d\left({\tilde{X}}_{R},{\tilde{\hat{X}}}_{R}\right)\right]$ is non-negative and is bounded above, by setting $a=\log|X_{H}|+D_{\max}$, it must hold that

$$\begin{array}{cc} \; & \alpha D(P_{{\tilde{X}}_{E^{c}}{\tilde{X}}_{E}{\tilde{\hat{X}}}_{R}}^{\alpha }\|Q_{X_{E^{c}}X_{E}{\tilde{\hat{X}}}_{R}}^{\alpha })\leq a \end{array}$$

and thus

$$\begin{array}{cc} \; & D\left(P_{{\tilde{X}}_{E^{c}}{\tilde{X}}_{E}{\tilde{\hat{X}}}_{R}}^{\alpha }\|Q_{X_{E^{c}}X_{E}{\tilde{\hat{X}}}_{R}}^{\alpha }\right)\leq \left(a/\alpha \right). \end{array}$$

Notice that any set of probability distributions on a finite alphabet forms a compact set. Because $G(P_{{\tilde{X}}_{E^{c}}{\tilde{X}}_{E}{\tilde{\hat{X}}}_{R}}^{\alpha })$ is a continuous function over a compact set, it is also uniformly continuous. Then, there exists a function Δ(t) satisfying Δ(t)→0 as t→0 such that

$$\begin{array}{cc} T_{E}^{\mu ,\alpha }\left(P_{X_{K}}\right) & \geq G(P_{{\tilde{X}}_{E^{c}}{\tilde{X}}_{E}{\tilde{\hat{X}}}_{R}}^{\alpha })\\ \; & \geq G\left(Q_{X_{E^{c}}X_{E}{\tilde{\hat{X}}}_{R}}^{\alpha }\right)-\Delta \left(a/\alpha \right)\\ \; & \geq T_{E}^{\mu }\left(P_{X_{K}}\right)-\Delta \left(a/\alpha \right). \end{array}$$

Consequently, we obtain the desired inequality $T_{E}^{\mu }\left(P_{X_{K}}\right)\leq \lim_{\alpha \to \infty }T_{E}^{\mu ,\alpha }\left(P_{X_{K}}\right)$ by taking α→∞. □

In the following proposition, we show the inequalities satisfied between i.i.d. source $P_{X_{E^{c}}^{n}X_{E}^{n}}$ and arbitrary source $P_{{\tilde{X}}_{E^{c}}^{n}{\tilde{X}}_{E}^{n}}$.

**Proposition** **2.** *For i.i.d. source $P_{X_{E^{c}}^{n}X_{E}^{n}}$, which has the common distribution $P_{X_{E^{c}}X_{E}}$ and arbitrary distribution $P_{{\tilde{X}}_{E^{c}}^{n}{\tilde{X}}_{E}^{n}}$, it holds that*

(135) $$\begin{array}{cc} H\left({\tilde{X}}_{E^{c}}^{n}|{\tilde{X}}_{E}^{n}\right)+D\left(P_{{\tilde{X}}_{E^{c}}^{n}{\tilde{X}}_{E}^{n}}\|P_{X_{E^{c}}^{n}X_{E}^{n}}\right)\; & \geq n[H\left({\tilde{X}}_{E^{c},J}|{\tilde{X}}_{E,J}\right)+D\left(P_{{\tilde{X}}_{E^{c},J}{\tilde{X}}_{E,J}}\|P_{X_{E^{c}}X_{E}}\right)], \end{array}$$

(136) $$\begin{array}{cc} H\left({\tilde{X}}_{H}^{n}\right)+D\left(P_{{\tilde{X}}_{H}^{n}{\tilde{X}}_{R}^{n}}\|P_{X_{H}^{n}X_{R}^{n}}\right)\; & \geq n[H\left({\tilde{X}}_{H,J}\right)+D\left(P_{{\tilde{X}}_{H,J}{\tilde{X}}_{R,J}}\|P_{X_{H}X_{R}}\right)], \end{array}$$

*where J∼unif(1,⋯,n) is the uniformly random variable over the set {1,2,⋯,n} for time-sharing and is assumed to be independent of all the other random variables involved.*

**Proof.**  The l.h.s. of (135) can be represented as

$$\begin{array}{cc} \; & H\left({\tilde{X}}_{E^{c}}^{n}|{\tilde{X}}_{E}^{n}\right)+D(P_{{\tilde{X}}_{E^{c}}^{n}|{\tilde{X}}_{E}^{n}}\|P_{X_{E^{c}}^{n}|X_{E}^{n}}\left|P_{{\tilde{X}}_{E}^{n}}\right)+D\left(P_{{\tilde{X}}_{E}^{n}}\|P_{X_{E}^{n}}\right). \end{array}$$

The sum of the first and second terms satisfies the following equation:

(137) $$\begin{array}{cc} \; & H\left({\tilde{X}}_{E^{c}}^{n}|{\tilde{X}}_{E}^{n}\right)+D(P_{{\tilde{X}}_{E^{c}}^{n}|{\tilde{X}}_{E}^{n}}\|P_{X_{E^{c}}^{n}|X_{E}^{n}}\left|P_{{\tilde{X}}_{E}^{n}}\right)\\ \; & =\sum_{x_{E^{c}}^{n},x_{E}^{n}}P_{{\tilde{X}}_{E^{c}}^{n}{\tilde{X}}_{E}^{n}}\left(x_{E^{c}}^{n},x_{E}^{n}\right)\\ \; & \;\cdot \left\{\log\frac{1}{P_{{\tilde{X}}_{E^{c}}^{n}|{\tilde{X}}_{E}^{n}}\left(x_{E^{c}}^{n}|x_{E}^{n}\right)}+\log\frac{P_{{\tilde{X}}_{E^{c}}^{n}|{\tilde{X}}_{E}^{n}}\left(x_{E^{c}}^{n}|x_{E}^{n}\right)}{P_{X_{E^{c}}^{n}|X_{E}^{n}}\left(x_{E^{c}}^{n}|x_{E}^{n}\right)}\right\}\\ \; & =\sum_{x_{E^{c}}^{n},x_{E}^{n}}P_{{\tilde{X}}_{E^{c}}^{n}{\tilde{X}}_{E}^{n}}\left(x_{E^{c}}^{n},x_{E}^{n}\right)\log\frac{1}{P_{X_{E^{c}}^{n}|X_{E}^{n}}\left(x_{E^{c}}^{n}|x_{E}^{n}\right)}\\ \; & \overset{\left(a\right)}{=}\sum_{x_{E^{c}}^{n},x_{E}^{n}}P_{{\tilde{X}}_{E^{c}}^{n}{\tilde{X}}_{E}^{n}}\left(x_{E^{c}}^{n},x_{E}^{n}\right)\cdot \left\{\sum_{j=1}^{n}\log\frac{1}{P_{X_{E^{c}}|X_{E}}\left(x_{E^{c},j}|x_{E,j}\right)}\right\}\\ \; & \overset{\left(b\right)}{=}n\sum_{x_{E^{c}},x_{E}}P_{{\tilde{X}}_{E^{c},J}{\tilde{X}}_{E,J}}\left(x_{E^{c}},x_{E}\right)\log\frac{1}{P_{X_{E^{c}}|X_{E}}\left(x_{E^{c}}|x_{E}\right)}\\ \; & =n\sum_{x_{E^{c}},x_{E}}P_{{\tilde{X}}_{E^{c},J}{\tilde{X}}_{E,J}}\left(x_{E^{c}},x_{E}\right)\\ \; & \;\cdot \left\{\log\frac{1}{P_{{\tilde{X}}_{E^{c},J}|{\tilde{X}}_{E,J}}\left(x_{E^{c}}|x_{E}\right)}+\log\frac{P_{{\tilde{X}}_{E^{c},J}|{\tilde{X}}_{E,J}}\left(x_{E^{c}}|x_{E}\right)}{P_{X_{E^{c}}|X_{E}}\left(x_{E^{c}}|x_{E}\right)}\right\}\\ \; & =n\{H\left({\tilde{X}}_{E^{c},J}|{\tilde{X}}_{E,J}\right)+D(P_{{\tilde{X}}_{E^{c},J}|{\tilde{X}}_{E,J}}\|P_{X_{E^{c}}|X_{E}}|P_{{\tilde{X}}_{E,J}})\}, \end{array}$$

where

(a) follows from the memoryless property of i.i.d. source $P_{X_{E^{c}}^{n}X_{E}^{n}}$;
(b) holds because $\frac{1}{n}\sum_{j=1}^{n}P_{{\tilde{X}}_{E^{c},j}{\tilde{X}}_{E,j}}\left(x_{E^{c}},x_{E}\right)=P_{{\tilde{X}}_{E^{c},J}{\tilde{X}}_{E,J}}\left(x_{E^{c}},x_{E}\right)$.

The third term can be bounded from below as

(138) $$\begin{array}{cc} D(P_{{\tilde{X}}_{E}^{n}}\|P_{X_{E}^{n}})\; & =\sum_{j=1}^{n}D(P_{{\tilde{X}}_{E},j|{\tilde{X}}_{E}^{j-1}}\|P_{X_{E}}\left|P_{{\tilde{X}}_{E}^{j-1}}\right)\\ \; & \overset{\left(c\right)}{\geq }\sum_{j=1}^{n}\;D\left(P_{{\tilde{X}}_{E},j}\|P_{X_{E}}\right)\\ \; & \overset{\left(d\right)}{\geq }nD\left(P_{{\tilde{X}}_{E},J}\|P_{X_{E}}\right), \end{array}$$

where

(c) follows from the data processing inequality and
(d) holds because of Jensen’s inequality.

From (137) and (138), (135) can be derived.

Likewise, the l.h.s. of (136) can be represented as

$$\begin{array}{c} H\left({\tilde{X}}_{H}^{n}\right)+D\left(P_{{\tilde{X}}_{H}^{n}}\|P_{X_{H}^{n}}\right)+D(P_{{\tilde{X}}_{R}^{n}|{\tilde{X}}_{H}^{n}}\|P_{X_{R}^{n}|X_{H}^{n}}\left|P_{{\tilde{X}}_{H}^{n}}\right), \end{array}$$

The sum of the first and second terms satisfies

(139) $$\begin{array}{cc} \; & H\left({\tilde{X}}_{H}^{n}\right)+D\left(P_{{\tilde{X}}_{H}^{n}}\|P_{X_{H}^{n}}\right)\\ \; & =\sum_{x_{H}^{n}}P_{{\tilde{X}}_{H}^{n}}\left(x_{H}^{n}\right)\left\{\log\frac{1}{P_{{\tilde{X}}_{H}^{n}}\left(x_{H}^{n}\right)}+\log\frac{P_{{\tilde{X}}_{H}^{n}}\left(x_{H}^{n}\right)}{P_{X_{H}^{n}}\left(x_{H}^{n}\right)}\right\}\\ \; & =\sum_{x_{H}^{n}}P_{{\tilde{X}}_{H}^{n}}\left(x_{H}^{n}\right)\log\frac{1}{P_{X_{H}^{n}}\left(x_{H}^{n}\right)}\\ \; & =\sum_{x_{H}^{n}}P_{{\tilde{X}}_{H}^{n}}\left(x_{H}^{n}\right)\cdot \left\{\sum_{j=1}^{n}\log\frac{1}{P_{X_{H}}\left(x_{H,j}\right)}\right\}\\ \; & \overset{\left(e\right)}{=}n\sum_{x_{H}}P_{{\tilde{X}}_{H},J}\left(x_{H}\right)\log\frac{1}{P_{X_{H}}\left(x_{H}\right)}\\ \; & =n\sum_{x_{H}}P_{{\tilde{X}}_{H},J}\left(x_{H}\right)\left\{\log\frac{1}{P_{{\tilde{X}}_{H},J}\left(x_{H}\right)}+\log\frac{P_{{\tilde{X}}_{H},J}\left(x_{H}\right)}{P_{X_{H}}\left(x_{H}\right)}\right\}\\ \; & =n\{H\left({\tilde{X}}_{H,J}\right)+D\left(P_{{\tilde{X}}_{H,J}}\|P_{X_{H}}\right)\}, \end{array}$$

where

(e) holds because $\frac{1}{n}\sum_{j=1}^{n}P_{{\tilde{X}}_{H,j}}\left(x_{H}\right)=P_{{\tilde{X}}_{H,J}}\left(x_{H}\right)$.

For the third term, it holds that

(140) $$\begin{array}{cc} D(P_{{\tilde{X}}_{R}^{n}|{\tilde{X}}_{H}^{n}}\|P_{X_{R}^{n}|X_{H}^{n}}|P_{{\tilde{X}}_{H}^{n}})\; & =\sum_{j=1}^{n}D(P_{{\tilde{X}}_{R,j}|{\tilde{X}}_{H}^{n}{\tilde{X}}_{R}^{j-1}}\|P_{X_{R}|X_{H}}\left|P_{{\tilde{X}}_{H}^{n}{\tilde{X}}_{R}^{j-1}}\right)\\ \; & \overset{\left(f\right)}{\geq }\sum_{j=1}^{n}D(P_{{\tilde{X}}_{R,j}|{\tilde{X}}_{H,j}}\|P_{X_{R}|X_{H}}\left|P_{{\tilde{X}}_{H,j}}\right)\\ \; & \geq nD(P_{{\tilde{X}}_{R,J}|{\tilde{X}}_{H,J}}\|P_{X_{R}|X_{H}}|P_{{\tilde{X}}_{H},J}), \end{array}$$

where

(f) follows from the log sum inequality.

From (139) and (140), we obtain (136). □

### 5.3. Strong Converse Theorem

We shall establish the strong converse theorem, which is the main result of this section. Before proving the theorem, we state the lemma of the key tool in the proof about a single-letterized $T_{E}^{\mu ,\alpha }\left(P_{X_{K}}\right)$ and a $T_{E}^{\mu ,\alpha }\left(P_{X_{K}}^{n}\right)$, which are introduced in Proposition 1.

**Lemma** **7.** *For any E such that R⊆E⊆K, all n∈N, μ≥0 and α>0, it holds that*

$$\begin{array}{c} T_{E}^{\mu ,\alpha }\left(P_{X_{K}}^{n}\right)\geq nT_{E}^{\mu ,\alpha }\left(P_{X_{K}}\right). \end{array}$$

As the main theorem of this section, we show the strong converse theorem for the utility–privacy trade-offs.

**Theorem** **4.** *Strong converse theorem: For any E such that R⊆E⊆K and all 0<ϵ<1, it holds that*

$$\begin{array}{c} L_{E}^{DL}\left(\epsilon |P_{X_{K}}\right)=L_{E}^{DL}\left(P_{X_{K}}\right). \end{array}$$

**Remark** **5.** *Theorem 4 suggests that regardless of the value of ϵ, the region $L_{E}^{DL}\left(\epsilon |P_{X_{K}}\right)$ is equal to $L_{E}^{DL}\left(P_{X_{K}}\right)$.*

### 5.4. Proof of Lemma 7

Lemma 7 indicates that the function $T_{E}^{\mu ,\alpha }\left(P_{X_{K}}^{n}\right)$, whose argument $P_{X_{K}}^{n}$ is a probability distribution over $X_{K}^{n}$, can be lower-bounded by the *n*-fold of a single-letterized function $T_{E}^{\mu ,\alpha }\left(P_{X_{K}}\right)$. Before describing the detailed proof, we state the outline of the proof: (i) We first express the function $T_{E}^{\mu ,\alpha }\left(P_{X_{K}}^{n}\right)$ as the maximum of the difference of two functions denoted by $G_{1}$ and $G_{2}$ as in (142). (ii) Then, we show that the first function $G_{1}$ can be lower-bounded by the *n*-fold of its single-letterized function as in (143), while the second function $G_{2}$ can be upper-bounded by the *n*-fold of its single-letterized function as in (147). This outline of the proof is similar to the Proof of Theorem 4, 16 with a slight modification of the function $G_{2}$.

For a given distribution $P_{{\tilde{X}}_{E^{c}}^{n}{\tilde{X}}_{E}^{n}{\tilde{\hat{X}}}_{R}^{n}}$, let functions $G_{1}\left(P_{{\tilde{X}}_{E^{c}}^{n}{\tilde{X}}_{E}^{n}}\right)$ and $G_{2}\left(P_{{\tilde{X}}_{E^{c}}^{n}{\tilde{X}}_{E}^{n}{\tilde{\hat{X}}}_{R}^{n}}\right)$ be defined as

(141) $$\begin{array}{cc} G_{1}\left(P_{{\tilde{X}}_{E^{c}}^{n}{\tilde{X}}_{E}^{n}}\right)≔\; & H\left({\tilde{X}}_{H}^{n}\right)+\alpha H\left({\tilde{X}}_{E^{c}}^{n}|{\tilde{X}}_{E}^{n}\right)+\left(\alpha +1\right)D\left(P_{{\tilde{X}}_{E^{c}}^{n}{\tilde{X}}_{E}^{n}}\|P_{X_{E^{c}}X_{E}}^{n}\right),\\ G_{2}\left(P_{{\tilde{X}}_{E^{c}}^{n}{\tilde{X}}_{E}^{n}{\tilde{\hat{X}}}_{R}^{n}}\right)≔\; & H\left({\tilde{X}}_{H}^{n}|{\tilde{\hat{X}}}_{R}^{n}\right)-\mu E\left[d\left({\tilde{X}}_{R}^{n},{\tilde{\hat{X}}}_{R}^{n}\right)\right]+\alpha H\left({\tilde{X}}_{E}^{n}|{\tilde{X}}_{E}^{n},{\tilde{\hat{X}}}_{R}^{n}\right). \end{array}$$

Using these functions, and in view of (134), $T_{E}^{\mu ,\alpha }\left(P_{X_{E^{c}}X_{E}}^{n}\right)$ can be written as

(142) $$\begin{array}{cc} \; & T_{E}^{\mu ,\alpha }\left(P_{X_{E^{c}}X_{E}}^{n}\right)=\min_{P_{{\tilde{X}}_{E^{c}}^{n}{\tilde{X}}_{E}^{n}{\tilde{\hat{X}}}_{R}^{n}}}\left[G_{1}\left(P_{{\tilde{X}}_{E^{c}}^{n}{\tilde{X}}_{E}^{n}}\right)-G_{2}\left(P_{{\tilde{X}}_{E^{c}}^{n}{\tilde{X}}_{E}^{n}{\tilde{\hat{X}}}_{R}^{n}}\right)\right]. \end{array}$$

For fixed $P_{{\tilde{X}}_{E^{c}}^{n}{\tilde{X}}_{E}^{n}{\tilde{\hat{X}}}_{R}^{n}}$, from Proposition 2, it holds that

(143) $$\begin{array}{c} G_{1}\left(P_{{\tilde{X}}_{E^{c}}^{n}{\tilde{X}}_{E}^{n}}\right)\geq nG_{1}\left(P_{{\tilde{X}}_{E^{c},J}{\tilde{X}}_{E,J}}\right). \end{array}$$

Next, we consider the function $G_{2}\left(P_{{\tilde{X}}_{E^{c}}^{n}{\tilde{X}}_{E}^{n}{\tilde{\hat{X}}}_{R}^{n}}\right)$. For the first term on the r.h.s. of (141), it holds that

(144) $$\begin{array}{cc} H({\tilde{X}}_{H}^{n}|{\tilde{\hat{X}}}_{R}^{n})\; & =\sum_{j=1}^{n}H\left({\tilde{X}}_{H,j}|{\tilde{X}}_{H}^{j-1},{\tilde{\hat{X}}}_{R}^{n}\right)\\ \; & \leq \sum_{j=1}^{n}H\left({\tilde{X}}_{H,j}|{\tilde{\hat{X}}}_{R,j}\right)\\ \; & =n\cdot \frac{1}{n}\sum_{j=1}^{n}H\left({\tilde{X}}_{H,j}|{\tilde{\hat{X}}}_{R,j}\right)\\ \; & =nH({\tilde{X}}_{H,J}|{\tilde{\hat{X}}}_{R,J},J)\\ \; & \leq nH({\tilde{X}}_{H,J}|{\tilde{\hat{X}}}_{R,J}). \end{array}$$

The second term of (141) can be expressed as follows:

(145) $$\begin{array}{cc} E[d\left({\tilde{X}}_{R}^{n},{\tilde{\hat{X}}}_{R}^{n}\right)]\; & =\sum_{x_{R}^{n},{\hat{x}}_{R}^{n}}P_{{\tilde{X}}_{R}^{n}{\tilde{\hat{X}}}_{R}^{n}}\left(x_{R}^{n},{\hat{x}}_{R}^{n}\right)\cdot \left\{\sum_{j=1}^{n}d\left(x_{R,j},{\hat{x}}_{R,j}\right)\right\}\\ \; & =\sum_{j=1}^{n}\sum_{x_{R},{\hat{x}}_{R}}P_{{\tilde{X}}_{R,j}{\tilde{\hat{X}}}_{R,j}}\left(x_{R},{\hat{x}}_{R}\right)d\left(x_{R},{\hat{x}}_{R}\right)\\ \; & \overset{\left(a\right)}{=}n\sum_{x_{R},{\hat{x}}_{R}}P_{{\tilde{X}}_{R,J}{\tilde{\hat{X}}}_{R,J}}\left(x_{R},{\hat{x}}_{R}\right)d\left(x_{R},{\hat{x}}_{R}\right)\\ \; & =nE[d\left({\tilde{X}}_{R,J},{\tilde{\hat{X}}}_{R,J}\right)], \end{array}$$

where

(a) follows from $\frac{1}{n}\sum_{j=1}^{n}P_{{\tilde{X}}_{R,j}{\tilde{\hat{X}}}_{R,j}}\left(x_{R},{\hat{x}}_{R}\right)=P_{{\tilde{X}}_{R,J}{\tilde{\hat{X}}}_{R,J}}\left(x_{R},{\hat{x}}_{R}\right)$.

Moreover, for the third term of (141), it holds that

(146) $$\begin{array}{cc} H({\tilde{X}}_{E^{c}}^{n}|{\tilde{X}}_{E}^{n},{\tilde{\hat{X}}}_{R}^{n})\; & =\sum_{j=1}^{n}H\left({\tilde{X}}_{E^{c},j}|{\tilde{X}}_{E^{c}}^{j-1},{\tilde{X}}_{E}^{n},{\tilde{\hat{X}}}_{R}^{n}\right)\\ \; & \leq \sum_{j=1}^{n}H\left({\tilde{X}}_{E^{c},j}|{\tilde{X}}_{E,j},{\tilde{\hat{X}}}_{R,j}\right)\\ \; & =n\cdot \frac{1}{n}\sum_{j=1}^{n}H\left({\tilde{X}}_{E^{c},j}|{\tilde{X}}_{E,j},{\tilde{\hat{X}}}_{R,j}\right)\\ \; & =nH({\tilde{X}}_{E^{c},J}|{\tilde{X}}_{E,J},{\tilde{\hat{X}}}_{R,J},J)\\ \; & \leq nH({\tilde{X}}_{E^{c},J}|{\tilde{X}}_{E,J},{\tilde{\hat{X}}}_{R,J}). \end{array}$$

From (144)–(146), we obtain

(147) $$\begin{array}{c} G_{2}\left(P_{{\tilde{X}}_{E^{c}}^{n}{\tilde{X}}_{E}^{n}{\tilde{\hat{X}}}_{R}^{n}}\right)\leq nG_{2}\left(P_{{\tilde{X}}_{E^{c},J}{\tilde{X}}_{E,J}{\tilde{\hat{X}}}_{R,J}}\right). \end{array}$$

Consequently, since (143) and (147) are satisfied for an arbitrary $P_{{\tilde{X}}_{E^{c}}^{n}{\tilde{X}}_{E}^{n}{\tilde{\hat{X}}}_{R}^{n}}$, the proof is completed.

### 5.5. Proof of Strong Converse Theorem

For any given ϵ>0, fix the rate pair $\left(D,L\right)\in L_{E}^{DL}\left(\epsilon |P_{X_{K}}\right)$ arbitrarily. Then, by definition, there exists a code $\left(f_{n},g_{n}\right)$ satisfying (79) and (80). For this code $\left(f_{n},g_{n}\right)$, a set D is defined as

$$\begin{array}{c} D≔\{\left(x_{E^{c}}^{n},x_{E}^{n}\right):\;\;d\left(x_{R}^{n},g_{n}\left(f_{n}\left(x_{E}^{n}\right)\right)\right)\leq nD\}. \end{array}$$

We derive a distribution $P_{{\tilde{X}}_{E^{c}}^{n}{\tilde{X}}_{E}^{n}}$ as

$$\begin{array}{c} P_{{\tilde{X}}_{E^{c}}^{n}{\tilde{X}}_{E}^{n}}\left(x_{E^{c}}^{n},x_{E}^{n}\right)≔\frac{P_{X_{E^{c}}X_{E}}^{n}\left(x_{E^{c}}^{n},x_{E}^{n}\right)1\;l\left[\left(x_{E^{c}}^{n},x_{E}^{n}\right)\in D\right]}{P_{X_{E^{c}X_{E}}}^{n}\left(D\right)}. \end{array}$$

It is obvious that the excess-distortion probability measured by $P_{{\tilde{X}}_{E^{c}}^{n}{\tilde{X}}_{E}^{n}}$ is 0; that is, ${\tilde{X}}_{R}^{n}$ and ${\tilde{\hat{X}}}_{R}^{n}=g_{n}\left(f_{n}\left({\tilde{X}}_{E}^{n}\right)\right)$ satisfy $E[d\left({\tilde{X}}_{R}^{n},{\tilde{\hat{X}}}_{R}^{n}\right)]\leq nD$. Thus, by imitating the proof approach of the standard weak converse theorem, it holds that

(148) $$\begin{array}{cc} \; & n\left(L+\mu D\right)\geq I\left({\tilde{X}}_{H}^{n};{\tilde{\hat{X}}}_{R}^{n}\right)+\mu E\left[d\left({\tilde{X}}_{R}^{n},{\tilde{\hat{X}}}_{R}^{n}\right)\right], \end{array}$$

(149) $$\begin{array}{cc} \; & D\left(P_{{\tilde{X}}_{E^{c}}^{n}{\tilde{X}}_{E}^{n}}\|P_{X_{E^{c}}X_{E}}^{n}\right)=\log\frac{1}{P_{X_{E^{c}}X_{E}}^{n}\left(D\right)}\leq \log\frac{1}{1-\epsilon }. \end{array}$$

From (148), the following equation is obtained:

$$\begin{array}{cc} n(L+\mu D)\; & \overset{\left(a\right)}{\geq }I\left({\tilde{X}}_{H}^{n};{\tilde{\hat{X}}}_{R}^{n}\right)+\mu E\left[d\left({\tilde{X}}_{R}^{n},{\tilde{\hat{X}}}_{R}^{n}\right)\right]\\ \; & \;+(\left(\alpha +1\right)D\left(P_{{\tilde{X}}_{E^{c}}^{n}{\tilde{X}}_{E}^{n}}\|P_{X_{E^{c}}X_{E}}^{n}\right)+\alpha I\left({\tilde{X}}_{E^{c}}^{n};{\tilde{\hat{X}}}_{R}^{n}|{\tilde{X}}_{E}^{n}\right))\\ \; & \;-\left(\alpha +1\right)\log\frac{1}{1-\epsilon }\\ \; & \overset{\left(b\right)}{\geq }T_{E}^{\mu ,\alpha }\left(P_{X_{K}}^{n}\right)-\left(\alpha +1\right)\log\frac{1}{1-\epsilon }, \end{array}$$

where

(a) follows from (149) and $I({\tilde{X}}_{E^{c}}^{n};{\tilde{\hat{X}}}_{R}^{n}|{\tilde{X}}_{E}^{n})=0$,
(b) is due to (134).

Since $T_{E}^{\mu ,\alpha }\left(P_{X_{K}}^{n}\right)\geq nT_{E}^{\mu ,\alpha }\left(P_{X_{K}}\right)$ from Lemma 7, we have

$$\begin{array}{cc} L+\mu D & \geq T_{E}^{\mu ,\alpha }\left(P_{X_{K}}\right)-\frac{\left(\alpha +1\right)}{n}\log\frac{1}{1-\epsilon }, \end{array}$$

and therefore

$$\begin{array}{cc} \underset{\alpha >0}{\sup}\left(L+\mu D\right) & \geq \underset{\alpha >0}{\sup}\left[T_{\epsilon }^{\mu ,\alpha }\left(P_{X_{K}}\right)-\frac{\left(\alpha +1\right)}{n}\log\frac{1}{1-\epsilon }\right]. \end{array}$$

Because $T_{E}^{\mu }\left(P_{X_{K}}\right)=\sup_{\alpha >0}T_{E}^{\mu ,\alpha }\left(P_{X_{K}}\right)$ from Proposition 1, it holds that for an arbitrary α>0,

$$\begin{array}{cc} L+\mu D & \geq T_{E}^{\mu }\left(P_{X_{K}}\right)-\frac{\left(\alpha +1\right)}{n}\log\frac{1}{1-\epsilon }. \end{array}$$

Hence, it holds that

(150) $$\begin{array}{cc} L+\mu D & \geq \lim_{n\to \infty }\left(T_{E}^{\mu }\left(P_{X_{K}}\right)-\frac{\left(\alpha +1\right)}{n}\log\frac{1}{1-\epsilon }\right)\\ \; & =T_{E}^{\mu }\left(P_{X_{K}}\right)\;\mathrm{for}\;\mathrm{every}\;\mu \geq 0. \end{array}$$

For the set of (D,L) satisfying (150), varying μ≥0 arbitrarily and taking the intersection, we have

(151) $$\begin{array}{c} \left(D,L\right)\in \underset{\mu \geq 0}{⋂}\left\{\left(D,L\right):\;L+\mu D\geq T_{E}^{\mu }\left(P_{X_{K}}\right)\right\}. \end{array}$$

From Theorem 3, the r.h.s. of (151) is equal to $L_{E}^{DL}\left(P_{X_{K}}\right)$. This proof is completed.

## 6. Discussion

### 6.1. Numerical Calculation of Coding Rate, Utility, and Privacy for Decoder

In this section, we show some numerical calculations of the achievable region $C_{E}^{RDL}\left(P_{X_{K}}\right)$ and $L_{E}^{RDL}\left(P_{X_{K}}\right)$ in Corollaries 1 and 2, respectively. In general, it is difficult to compute the achievable region $C_{E}^{RDL}\left(P_{X_{K}}\right)$ and $L_{E}^{RDL}\left(P_{X_{K}}\right)$. Nevertheless, to obtain some insight, let us consider the three tractable but essential cases. In these calculations, the number of public attributes is one (|R|=1) and the number of private attributes is two (|H|=2). We assume that each of the attributes is binary. Here, note again that the coding rate *R* acts like the rate-distortion function in rate-distortion theory (cf. (Section 10 in [27])). For fixed *D* and *L*, a smaller coding rate is better.

**Figure 4:**
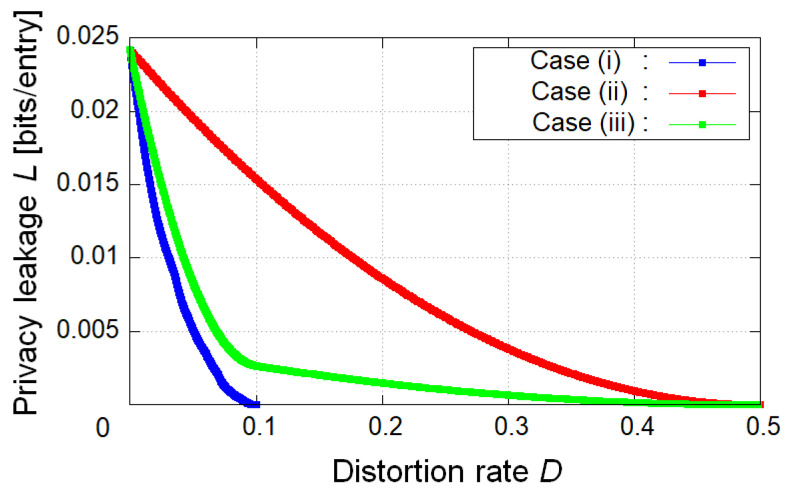
Utility–privacy trade-off region in cases (i), (ii), and (iii).

**Figure 5:**
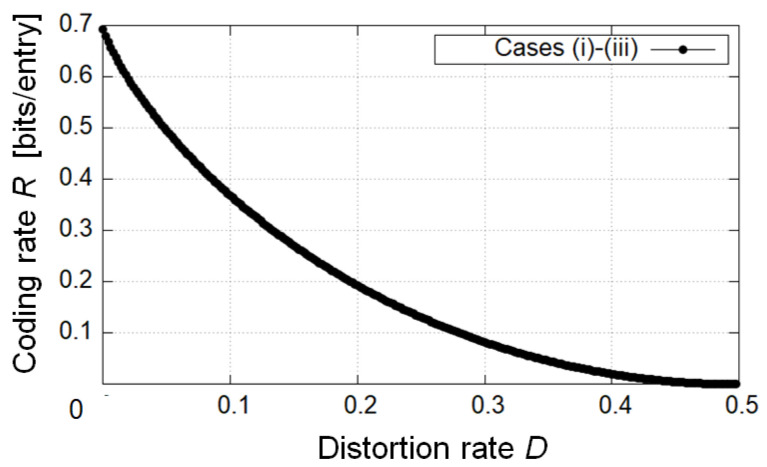
Utility–coding-rate trade-off region in cases (i), (ii), and (iii). The curves coincide in all cases.

In the first example, we calculated the *L*-*D* graph of theoretical limits in case (i) E=K, case (ii) E=R, and case (iii) R⊂E⊂K (Figure 4). As a result, the achievable privacy leakage *L* becomes small as *D* becomes large if we do not impose any restrictions on the value of *R*. For a given *D*, the privacy leakage for the decoder in case (i) E=K is the smallest, and the one in case (ii) E=R is the largest in all cases. The second example calculated the *R*-*D* graph of theoretical limits in cases (i), (ii), and (iii) (Figure 5). We can see that the minimum coding rates for a given *D* coincide in all cases if we do not impose any restrictions on the value of *L*. In the third example, we calculated the optimal privacy leakage *L* for fixed *D* and the corresponding coding rates *R* in cases (i), (ii), and (iii) (Table 1, Table 2 and Table 3). As a result, the optimal privacy leakage in cases (i) and (iii) is smaller than the one in case (ii), whereas for the optimal privacy leakage, the achievable coding rates in cases (i) and (iii) is larger than the one in case (ii).

Next, we discuss these results. In Figure 4, in comparison with each case, we can verify that for a given *D*, the more private information is encoded, the smaller the achievable minimum privacy leakage is. Figure 5 suggests that if the coding rate should be minimized, it suffices to encode only the public attributes. This result is evident from Corollaries 1 and 2 because the condition on the choice of test channel $P_{{\hat{X}}_{R}|X_{E}}$ in case (i) is weaker than the one in case (ii), and if an appropriate test channel is taken in case (i), it is also appropriate in case (ii). It is indicated that the achievable region in case (ii) is also the one in cases (i) and (iii). The opposite is not the case. From Table 1, Table 2 and Table 3, we can confirm the trade-off between the optimal privacy leakage *L* for a fixed *D* and the corresponding coding rate *R* in comparison with each case.

Summarizing the foregoing arguments, we have discussed the relationship between utility and privacy in Figure 4, the one between utility and coding rate in Figure 5, and the one between privacy and coding rate in Table 1, Table 2 and Table 3. From the discussion about Figure 5, some readers may suspect that case (i) is the best-encoded information because the achievable region in cases (ii) and (iii) is the one in case (i). This is true if we do not consider the leakage for the encoder. However, this is not true if we consider the leakage for the encoder, that is, the measurement of privacy for the encoder (see (12) or (76)). In the next section, we discuss this point in detail.

### 6.2. Significance of Limited Leakage for Encoder

In this section, we discuss the significance of evaluating the leakage for the encoder. Our goal of this discussion is to show that the best-encoded information may be case (iii) R⊂E⊂K if we take the limited leakage for the encoder into consideration.

The first issue is the amount of encoded information. Some readers may think that it is better that more encoded information is inputted into the encoder. However, there are pros and cons.

**Pros:**  The achievable regions $C_{E}^{RDL}\left(P_{X_{K}}\right)$ and $L_{E}^{RDL}\left(P_{X_{K}}\right)$ become larger.
**Cons:**  The leakage for the encoder increases.

From this point of view, we can come up with the idea that there exists the best-encoded information in case (iii) R⊂E⊂K if we impose some constraint on the leakage for the encoder. This idea is the key point of this paper.

**Figure 6:**
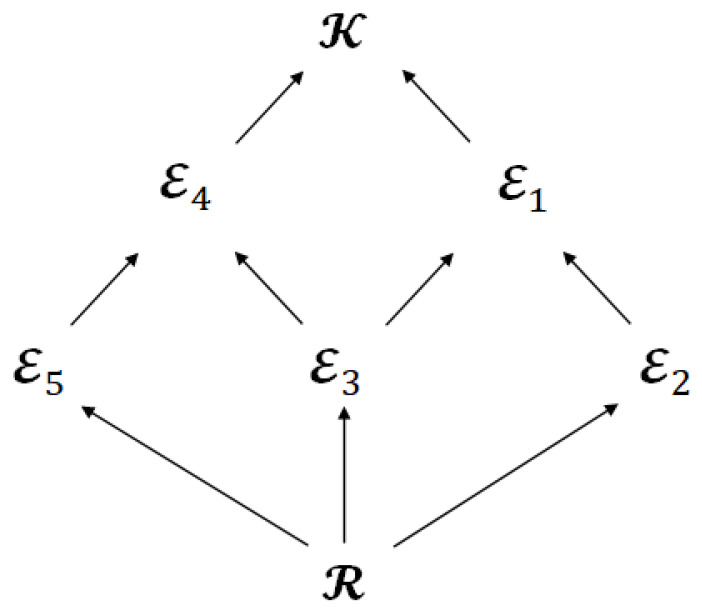
Hasse diagram that represents the inclusion relation for the index sets of attributes.

The second issue is the significance of the limited leakage for the encoder. Figure 6 shows the Hasse diagram, which represents the inclusion relation about the index sets of attributes. The Hasse diagram is often used to represent inclusion relations, for example, $R\subset E_{2}\subset E_{1}\subset K$.

We can also regard Figure 6 as the Hasse diagram that represents the inclusion relation for the achievable regions $C_{E}^{RDL}\left(P_{X_{K}}\right)$ and $L_{E}^{RDL}\left(P_{X_{K}}\right)$ because the index sets of attributes (R⊆E⊆K) corresponds to the encoded information ($X_{E}$) and the encoded information corresponds to the achievable region ($C_{E}^{RDL}\left(P_{X_{K}}\right)$ and $L_{E}^{RDL}\left(P_{X_{K}}\right)$). In addition, the diagram in Figure 6 has another property, which is that the superordinate sets have a larger amount of privacy leakage for the encoder than the subordinate sets since the index sets of attributes correspond to the privacy leakage for the encoder.

Let us consider a practical application. We assume that the data aggregator, that is, the encoder, tries to gather encoded information from some application user and hopes to develop the utility of the application while limiting the amount of leakage for $X_{H}^{n}$ by E≥0, that is, $e_{n}\leq E$. More precisely, for a given *E*, we want to find which subsets of K are sufficient to characterize

$$\begin{array}{c} C^{RDL}\left(P_{X_{K}}|E\right)≔\underset{R\subseteq E\subseteq K}{⋃}\left\{\left(R,D,L\right):\;\left(R,D,L,E\right)\in C_{E}\left(P_{X_{K}}\right)\right\},\\ L^{RDL}\left(P_{X_{K}}|E\right)≔\underset{R\subseteq E\subseteq K}{⋃}\left\{\left(R,D,L\right):\;\left(R,D,L,E\right)\in L_{E}\left(P_{X_{K}}\right)\right\}, \end{array}$$

where $C_{E}\left(P_{X_{K}}\right)$ and $L_{E}\left(P_{X_{K}}\right)$ are defined in Definitions 2 and 11, respectively. The process is as follows.

Step 1: Check the user’s requirements and impose the restriction on the privacy leakage for the encoder.

**Figure 7:**
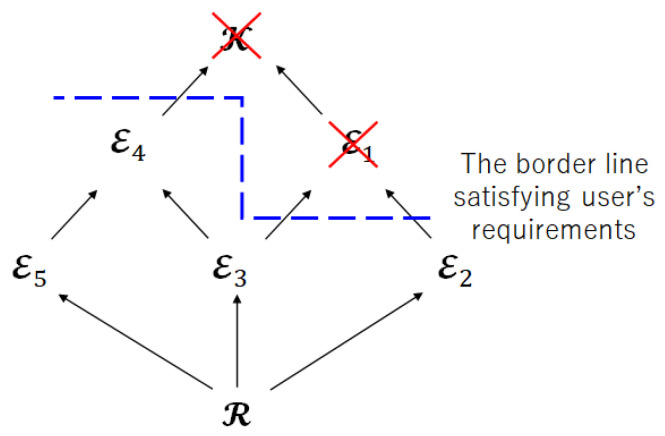
Hasse diagram for Step 1.

Figure 7 shows the Hasse diagram for Step 1. The blue dotted line means the border line satisfies the restriction of the privacy leakage for the encoder. Therefore, the index sets $E_{1}$ and K are not suitable as the index sets of encoded information.

Step 2: Check the inclusion relation between index sets.

**Figure 8:**
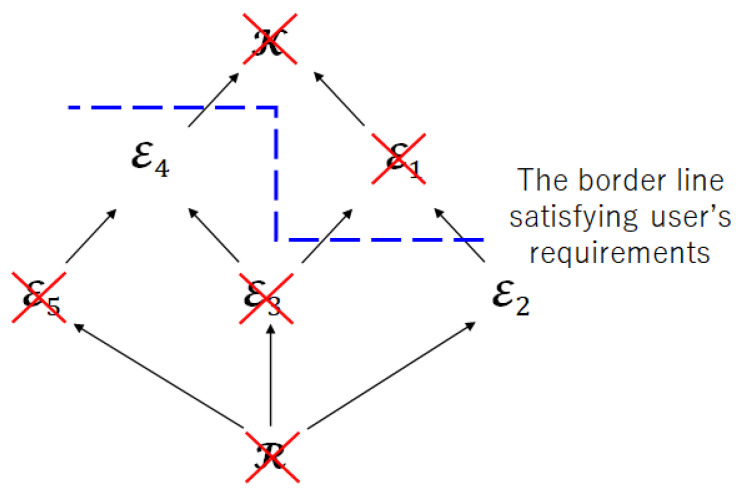
Hasse diagram for Step 2.

Figure 8 shows the Hasse diagram for Step 2. From Figure 6, we can find that

$$\begin{array}{c} R\subset E_{2},\;\;R\subset E_{3},\;\;R\subset E_{5},\;\;E_{3}\subset E_{4},\;\;E_{5}\subset E_{4}. \end{array}$$

Therefore, the index sets R, $E_{3}$, and $E_{5}$ are not suitable as the index sets of encoded information.

**Figure 9:**
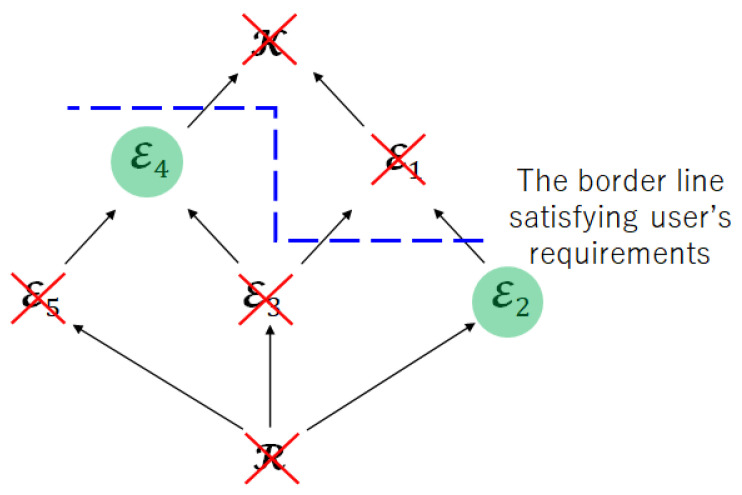
Hasse diagram obtained after Step 2.

Figure 9 shows the Hasse diagram obtained after Step 2. From Figure 9, the remaining index sets are $E_{2}$ and $E_{4}$. Therefore, if we impose restriction on privacy leakage for the encoder, the index sets $E_{2}$ or $E_{4}$ form the Pareto area in this multi-objective optimization problem. In other words, there exists a system that satisfies the user’s requirements *E* of the maximum amount of leakage to the encoder, and the achievable regions are given by $C^{RDL}\left(P_{X_{K}}|E\right)=C_{E_{2}}^{RDL}\left(P_{X_{K}}\right)\cup C_{E_{4}}^{RDL}\left(P_{X_{K}}\right)$ and $L^{RDL}\left(P_{X_{K}}|E\right)=L_{E_{2}}^{RDL}\left(P_{X_{K}}\right)\cup L_{E_{4}}^{RDL}\left(P_{X_{K}}\right)$.

From the discussion above, we mention that the best-encoded information is case (iii) R⊂E⊂K if we take the limited leakage for the encoder into account. This concept is one of the most important novelties in this paper.

If *E* satisfies some condition, then $C^{RDL}\left(P_{X_{K}}|E\right)$ can be characterized by the expressions given by Yamamoto [1] (cf. Remark 3). More specifically, the region $C^{RDL}\left(P_{X_{K}}|E\right)$ can be given by

$$\begin{array}{cc} C^{RDL}\left(P_{X_{K}}|E\right) & =S_{K}^{RDL}\left(P_{X_{K}}\right) \end{array}$$

if $E\geq H(X_{K})$ and

$$\begin{array}{cc} C^{RDL}\left(P_{X_{K}}|E\right) & =S_{R}^{RDL}\left(P_{X_{K}}\right) \end{array}$$

if $H\left(X_{R}\right)\leq E<H\left(X_{E}\right)$ for any R⊂E with R≠E, where the regions $S_{K}^{RDL}\left(P_{X_{K}}\right)$ and $S_{R}^{RDL}\left(P_{X_{K}}\right)$ are given in [1] (cf. Remark 3).

### 6.3. Discussion on Measures for Privacy Leakage

This paper adopts the mutual information as the measure of privacy leakage as in (12), (13), (76), and (77). However, some less likely data can be leaked even though the database satisfies the theoretical limit of privacy leakage. For example, let (X,Y) be a pair of correlated random variables whose I(X;Y) is very small. However, there may exist a pair of $\left(x_{1},y_{1}\right)$ such that $Y=y_{1}$ can imply $X=x_{1}$ with high probability. To put it differently, the receiver can tell the value of *X* if it observes $Y=y_{1}$. The theoretical limit evaluated with mutual information cannot prevent such a scenario. To circumvent this scenario, we suggest the other measurement adopted in related studies. A promising candidate to avoid this problem is to employ Rényi information of higher orders [30], maximal leakage [15], and maximal α-leakage [16,17,18,21].

## 7. Conclusions

In this paper, we strengthened the results in [3] mainly by establishing three coding theorems in a privacy-constrained source coding problem. In Section 3 and Section 4, two theorems are made about the first-order rate analysis in which utility is measured by the expected distortion or the excess-distortion probability for case (iii), R⊂E⊂K. The novelty is the introduction of the measure of privacy for the encoder along with the use of the excess-distortion probability. The obtained characterization reduces to the one given in [3] derived based on the expected distortion when the leakage for the encoder is not limited, and the result shows that employing an excess-distortion probability does not change the achievable region from the one with an expected distortion. In Section 5, we establish the strong converse theorem for utility–privacy trade-offs. Although the described result is for the projected plane of utility and privacy for the decoder for simplicity, we can also incorporate the measure of privacy for the encoder. Finally, we discuss the significance of the encoded information considering limited leakage for the encoder. The argument suggests that the best-encoded information can be case (iii) R⊂E⊂K if some constraint is imposed on the privacy leakage for the encoder.

As future work, the second-order rate analysis for utility–privacy trade-offs is an interesting research topic [4,5,6]. Moreover, the strong converse theorem and the second-order rate analysis for the four-dimensional region of coding rate, utility, privacy for the decoder, and privacy for the encoder are more challenging tasks. It is also worth analyzing the achievable region with the other privacy measures such as Rényi information [30], maximal leakage [15], and maximal α-leakage [16,17,18,21]. This paper analyzed the theoretical limits of coding, but understanding how to achieve the theoretical limits remains open. The construction of good codes is also an important subject. Extensions of this paper’s scenario to coding with side information [2,25] are also of interest. 

## Figures and Tables

**Table 1:** Minimum *L* and its corresponding *R* for D=0.0500.

Cases	Leakage *L*	Coding Rate *R*
case (ii)	0.019512	0.494629
case (iii)	0.008298	0.527700
case (i)	0.005107	0.539478

**Table 2:** Minimum *L* and its corresponding *R* for D=0.100.

Cases	Leakage *L*	Coding Rate *R*
case (ii)	0.015378	0.368062
case (iii)	0.002656	0.418826
case (i)	0.000000	0.429490

**Table 3:** Minimum *L* and its corresponding *R* for D=0.1500.

Cases	Leakage *L*	Coding Rate *R*
case (ii)	0.011748	0.270436
case (iii)	0.002032	0.294424
case (i)	0.000000	0.382211

## Data Availability

Not applicable.

## Appendix A. Proof of the Markov Chain $X_{E^{c}}$–$X_{E}$–${\hat{X}}_{R}$ in Converse Part of Theorem 1

Let $p_{i}\left(x_{E,i},x_{E^{c},i},{\hat{x}}_{R,i}\right)$ be the conditional distribution given Q=i,

(A1) $$\begin{array}{cc} p_{i}\left(x_{E,i},x_{E^{c},i},{\hat{x}}_{R,i}\right)\; & =\sum_{\begin{array}{c} x_{E,k}:\\ k\neq i \end{array}}\sum_{\begin{array}{c} x_{E^{c},k}:\\ k\neq i \end{array}}\sum_{\begin{array}{c} {\hat{x}}_{R,k}:\\ k\neq i \end{array}}p\left(x_{E}^{n},x_{E^{c}}^{n},{\hat{x}}_{R}^{n}\right)\\ \; & =\sum_{\begin{array}{c} x_{E,k}:\\ k\neq i \end{array}}\sum_{\begin{array}{c} x_{E^{c},k}:\\ k\neq i \end{array}}p\left(x_{E}^{n},x_{E^{c}}^{n},{\hat{x}}_{R,i}\right)\\ \; & \overset{\left(a\right)}{=}\sum_{\begin{array}{c} x_{E,k}:\\ k\neq i \end{array}}\sum_{\begin{array}{c} x_{E^{c},k}:\\ k\neq i \end{array}}p_{i}\left(x_{E}^{n},{\hat{x}}_{R,i}\right)p\left(x_{E^{c}}^{n}|x_{E}^{n}\right)\\ \; & =\sum_{\begin{array}{c} x_{E,k}:\\ k\neq i \end{array}}p_{i}\left(x_{E}^{n},{\hat{x}}_{R,i}\right)\sum_{\begin{array}{c} x_{E^{c},k}:\\ k\neq i \end{array}}p\left(x_{E^{c}}^{n}|x_{E}^{n}\right)\\ \; & \overset{\left(b\right)}{=}\sum_{\begin{array}{c} x_{E,k}:\\ k\neq i \end{array}}p_{i}\left(x_{E}^{n},{\hat{x}}_{R,i}\right)\cdot \\ \; & \;\sum_{\begin{array}{c} x_{E^{c},k}:\\ k\neq i \end{array}}\left(\prod_{l=1}^{n}p\left(x_{E^{c},l}|x_{E,l}\right)\right)\\ \; & =p_{i}\left(x_{E,i},{\hat{x}}_{R,i}\right)p\left(x_{E^{c},i}|x_{E,i}\right)\\ \; & =p\left(x_{E,i}\right)p\left(x_{E^{c},i}|x_{E,i}\right)p_{i}\left({\hat{x}}_{R,i}|x_{E,i}\right)\\ \; & =p\left(x_{E,i},x_{E^{c},i}\right)p_{i}\left({\hat{x}}_{R,i}|x_{E,i}\right), \end{array}$$

where

(a) is due to the Markov chain $X_{E^{c}}^{n}$–$X_{E}^{n}$–${\hat{X}}_{R,i}$ and
(b) follows from the stationary memoryless source.

Therefore, we can obtain the Markov chain $X_{E^{c},i}$–$X_{E,i}$–${\hat{X}}_{R,i}$. For the marginal distribution, we can show that

(A2) $$\begin{array}{cc} p(x_{E},x_{E^{c}},{\hat{x}}_{R})\; & \overset{\left(c\right)}{=}\frac{1}{n}\sum_{i=1}^{n}p_{i}\left(x_{E},x_{E^{c}},{\hat{x}}_{R}\right)\\ \; & \overset{\left(d\right)}{=}\frac{1}{n}\sum_{i=1}^{n}p_{i}\left(x_{E},x_{E^{c}}\right)p_{i}\left({\hat{x}}_{R}|x_{E}\right)\\ \; & \overset{\left(e\right)}{=}p\left(x_{E},x_{E^{c}}\right)\cdot \frac{1}{n}\sum_{i=1}^{n}p_{i}\left({\hat{x}}_{R}|x_{E}\right)\\ \; & \overset{\left(f\right)}{=}p\left(x_{E},x_{E^{c}}\right)p\left({\hat{x}}_{R}|x_{E}\right), \end{array}$$

where

(c) follows because
  (A3) $$\begin{array}{cc} p(x_{E},x_{E^{c}},{\hat{x}}_{R})\; & =\sum_{i=1}^{n}\mathrm{Pr}\left\{Q=i\right\}p_{i}\left(x_{E},x_{E^{c}},{\hat{x}}_{R}\right), \end{array}$$
(d) is due to the Markov chain $X_{E^{c},i}$–$X_{E,i}$–${\hat{X}}_{R,i}$,
(e) follows from the stationary memoryless source, and
(f) follows because
  (A4) $$\begin{array}{cc} p({\hat{x}}_{R}|x_{E}) & =\sum_{i=1}^{n}\mathrm{Pr}\left\{Q=i\right\}p_{i}\left({\hat{x}}_{R}|x_{E}\right). \end{array}$$

Therefore, we can obtain the Markov chain $X_{E^{c}}$–$X_{E}$–${\hat{X}}_{R}$. We complete the proof.

## Appendix B. Proof of Equation (56)

From $\tilde{A}\left(j\right)\subseteq B\left(j\right)$ for $j=1,2,\ldots ,M_{n}-1$,

(A5) $$\begin{array}{c} \mathrm{Pr}\left\{X_{K}^{n}\in B\left(j\right)\right\}=\mathrm{Pr}\left\{X_{K}^{n}\in \tilde{A}\left(j\right)\right\}+\mathrm{Pr}\left\{X_{K}^{n}\in B\left(j\right)\setminus \tilde{A}\left(j\right)\right\}. \end{array}$$

If $x_{K}^{n}\in B\left(j\right)\setminus \tilde{A}\left(j\right)$, then $x_{E}^{n}\in T_{\delta }^{n}\left(X_{E}|{\hat{x}}_{R}^{n}\left(j\right)\right)$ and $\left(x_{E}^{n},x_{E^{c}}^{n}\right)\notin T_{2\delta }^{n}\left(X_{K}|{\hat{x}}_{R}^{n}\left(j\right)\right)$, and thus we have $x_{E^{c}}^{n}\notin T_{\delta }^{n}\left(X_{E^{c}}|x_{E}^{n},{\hat{x}}_{R}^{n}\left(j\right)\right)$ from Lemma 5. Then,

(A6) $$\begin{array}{cc} x_{K}^{n}\in B\left(j\right)\setminus \tilde{A}\left(j\right)⟹\; & x_{E}^{n}\in T_{\delta }^{n}\left(X_{E}|{\hat{x}}_{R}^{n}\left(j\right)\right),\\ \; & x_{E^{c}}^{n}\notin T_{\delta }^{n}\left(X_{E^{c}}|x_{E}^{n},{\hat{x}}_{R}^{n}\left(j\right)\right) \end{array}$$

We can prove that

(A7) $$\begin{array}{cc} \; & \mathrm{Pr}\{X_{K}^{n}\in B\left(j\right)\setminus \tilde{A}\left(j\right)\}\\ \; & \leq \mathrm{Pr}\{X_{E}^{n}\in T_{\delta }^{n}\left(X_{E}|{\hat{x}}_{R}^{n}\left(j\right)\right),X_{E^{c}}^{n}\notin T_{\delta }^{n}\left(X_{E^{c}}|X_{E}^{n},{\hat{x}}_{R}^{n}\left(j\right)\right)\}\\ \; & =\sum_{x_{E}^{n}\in T_{\delta }^{n}\left(X_{E}|{\hat{x}}_{R}^{n}\left(j\right)\right)}\mathrm{Pr}\left\{X_{E}^{n}=x_{E}^{n}\right\}\cdot \\ \; & \;\mathrm{Pr}\{X_{E^{c}}^{n}\notin T_{\delta }^{n}\left(X_{E^{c}}|x_{E}^{n},{\hat{x}}_{R}^{n}\left(j\right)\right)|X_{E}^{n}=x_{E}^{n}\}\\ \; & \overset{\left(a\right)}{=}\sum_{x_{E}^{n}\in T_{\delta }^{n}\left(X_{E}|{\hat{x}}_{R}^{n}\left(j\right)\right)}\mathrm{Pr}\left\{X_{E}^{n}=x_{E}^{n}\right\}\cdot \\ \; & \;\mathrm{Pr}\{X_{E^{c}}^{n}\notin T_{\delta }^{n}\left(X_{E^{c}}|x_{E}^{n},{\hat{x}}_{R}^{n}\left(j\right)\right)|X_{E}^{n}=x_{E}^{n},{\hat{X}}_{R}^{n}={\hat{x}}_{R}^{n}\left(j\right)\}\\ \; & \overset{\left(b\right)}{\leq }\sum_{x_{E}^{n}\in T_{\delta }^{n}\left(X_{E}|{\hat{x}}_{R}^{n}\left(j\right)\right)}\mathrm{Pr}\left\{X_{E}^{n}=x_{E}^{n}\right\}\cdot 2|X_{E^{c}}\left|\cdot \right|X_{E}\left|\cdot \right|{\hat{X}}_{R}|e^{-2\delta^{2}n}\\ \; & \leq 2|X_{K}\left|\cdot \right|{\hat{X}}_{R}|e^{-2\delta^{2}n}, \end{array}$$

where

(a) is due to the Markov chain $X_{E^{c}}^{n}$–$X_{E}^{n}$–${\hat{X}}_{R}^{n}$ and
(b) follows from Lemma 6.

From Equations (A5) and (A7), we can obtain

(A8) $$\begin{array}{c} |\mathrm{Pr}\left\{X_{K}^{n}\in B\left(j\right)\right\}-\mathrm{Pr}\left\{X_{K}^{n}\in \tilde{A}\left(j\right)\right\}\left|\leq 2\right|X_{K}\left|\cdot \right|{\hat{X}}_{R}|e^{-2\delta^{2}n}. \end{array}$$

We complete the proof of (56).

## Appendix C. Proof of Existence of Code Satisfying Equations (57)–(62)

We first set $M_{n}≔2^{nR}$ and $r_{n}≔\frac{1}{n}\log M_{n}$. Then, we obviously have (57).

From the union upper bound,

(A9) $$\begin{array}{cc} \; & \mathrm{Pr}\left\{X_{E}^{n}\notin \overset{M_{n}-1}{\underset{j=1}{⋃}}A\left(j\right)\right\}\\ \; & \leq \mathrm{Pr}\{X_{E}^{n}\notin T_{\delta }^{n}\left(X_{E}\right)\}\\ \; & \;+\mathrm{Pr}\{X_{E}^{n}\in T_{\delta }^{n}\left(X_{E}\right),X_{E}^{n}\notin T_{\delta }^{n}\left(X_{E}|{\hat{x}}_{R}^{n}\left(j\right)\right)\\ \; & \;\;\mathrm{for}\;\mathrm{all}\;j=1,2,\ldots ,M_{n}-1\}. \end{array}$$

From Lemma 6, the first term in (A9) is bounded as

(A10) $$\begin{array}{c} \mathrm{Pr}\left\{X_{E}^{n}\notin T_{\delta }^{n}\left(X_{E}\right)\right\}\leq 2\left|X_{E}\right|e^{-2\delta^{2}n}. \end{array}$$

We consider the expectation of the second term in (A9) by random coding. Hereafter, we denote the random variable corresponding to the reproduced sequence ${\hat{x}}_{R}^{n}\left(j\right)$ as ${\hat{X}}_{R}^{n}\left(j\right)$. For notational simplicity, we use the abbreviation

(A11) $$\begin{array}{cc} \; & \mathrm{Pr}\{X_{E}^{n}\notin T_{\delta }^{n}\left(X_{E}|{\hat{X}}_{R}^{n}\left(j\right)\right)\\ \; & \;\mathrm{for}\;\mathrm{all}\;j=1,2,\ldots ,M_{n}-1\left|X_{E}^{n}=x_{E}^{n}\right\}\\ \; & =\mathrm{Pr}\{x_{E}^{n}\notin T_{\delta }^{n}\left(X_{E}|{\hat{X}}_{R}^{n}\left(j\right)\right)\\ \; & \;\mathrm{for}\;\mathrm{all}\;j=1,2,\ldots ,M_{n}-1\}, \end{array}$$

and then

(A12) $$\begin{array}{cc} \; & E[\mathrm{Pr}\{X_{E}^{n}\in T_{\delta }^{n}\left(X_{E}\right),X_{E}^{n}\notin T_{\delta }^{n}\left(X_{E}|{\hat{X}}_{R}^{n}\left(j\right)\right)\\ \; & \;\mathrm{for}\;\mathrm{all}\;j=1,2,\ldots ,M_{n}-1\}]\\ \; & =\sum_{x_{E}^{n}\in T_{\delta }^{n}\left(X_{E}\right)}p\left(x_{E}^{n}\right)E[\mathrm{Pr}\{X_{E}^{n}\notin T_{\delta }^{n}\left(X_{E}|{\hat{X}}_{R}^{n}\left(j\right)\right)\\ \; & \;\mathrm{for}\;\mathrm{all}\;j=1,2,\ldots ,M_{n}-1\left|X_{E}^{n}=x_{E}^{n}\right\}]\\ \; & \overset{\left(a\right)}{=}\sum_{x_{E}^{n}\in T_{\delta }^{n}\left(X_{E}\right)}p\left(x_{E}^{n}\right)E[\mathrm{Pr}\{x_{E}^{n}\notin T_{\delta }^{n}\left(X_{E}|{\hat{X}}_{R}^{n}\left(j\right)\right)\\ \; & \;\mathrm{for}\;\mathrm{all}\;j=1,2,\ldots ,M_{n}-1\}]\\ \; & =\sum_{x_{E}^{n}\in T_{\delta }^{n}\left(X_{E}\right)}p\left(x_{E}^{n}\right)\left(\prod_{j=1}^{M_{n}-1}E\left[\mathrm{Pr}\{x_{E}^{n}\notin T_{\delta }^{n}\left(X_{E}|{\hat{X}}_{R}^{n}\left(j\right)\right)\}\right]\right)\\ \; & \overset{\left(b\right)}{=}\sum_{x_{E}^{n}\in T_{\delta }^{n}\left(X_{E}\right)}p\left(x_{E}^{n}\right){\left(E\left[\mathrm{Pr}\{x_{E}^{n}\notin T_{\delta }^{n}\left(X_{E}|{\hat{X}}_{R}^{n}\left(1\right)\right)\}\right]\right)}^{M_{n}-1}\\ \; & \overset{\left(c\right)}{\leq }\exp\left\{-2^{n(R-I\left(X_{E};{\hat{X}}_{R}\right)-\frac{1}{n}-\tau )}\right\}\\ \; & \overset{\left(d\right)}{\leq }\exp\left\{-2^{2\delta^{2}n}\right\}, \end{array}$$

where

(a) is owing to (A11),
(b) is due to the symmetry about indexes of random coding,
(c) follows from the same way as in (Section 3.6.3 in [31]), and
(d) because δ is fixed to satisfy (49).

From (A10) and (A12), we obtain

(A13) $$\begin{array}{c} E\left[\mathrm{Pr}\left\{X_{E}^{n}\notin \overset{M_{n}-1}{\underset{j=1}{⋃}}A\left(j\right)\right\}\right]\leq \left(2\right|X_{E}\left|+1\right)e^{-2\delta^{2}n}. \end{array}$$

Therefore, there exists at least one codebook satisfying (60) in the ensembles obtained by random coding.

Hereafter, codebook C is fixed to satisfy (60). That is, codebook C satisfies

(A14) $$\begin{array}{c} \mathrm{Pr}\left\{X_{E}^{n}\notin \overset{M_{n}-1}{\underset{j=1}{⋃}}A\left(j\right)\right\}\leq \left(2\right|X_{E}\left|+1\right)e^{-2\delta^{2}n}. \end{array}$$

We evaluate the distortion function for each *j*.

(i) $j=1,2,\ldots ,M_{n}-1$:
  (A15) $$\begin{array}{cc} \; & d(x_{R}^{n},{\hat{x}}_{R}^{n}\left(j\right))\\ \; & =\frac{1}{n}\sum_{a\in X_{R}}\sum_{b\in {\hat{X}}_{R}}N\left(a,b|x_{R}^{n},{\hat{x}}_{R}^{n}\left(j\right)\right)d\left(a,b\right)\\ \; & \overset{\left(e\right)}{\leq }\sum_{a\in X_{R}}\sum_{b\in {\hat{X}}_{R}}P_{X_{R},{\hat{X}}_{R}}\left(a,b\right)d\left(a,b\right)\\ \; & \;+\left(\delta +\delta_{1}\right)|X_{R}\left|\cdot \right|{\hat{X}}_{R}|D_{\max}\\ \; & =E\left[d\left(X_{R},{\hat{X}}_{R}\right)\right]+\left(\delta +\delta_{1}\right)|X_{R}\left|\cdot \right|{\hat{X}}_{R}|D_{\max}, \end{array}$$
  where
  (e) because from Lemma 4, if $x_{E}^{n}\in T_{\delta }^{n}\left(X_{E}|{\hat{x}}_{R}^{n}\left(j\right)\right)$, then $x_{R}^{n}\in T_{\delta_{1}}^{n}\left(X_{R}|{\hat{x}}_{R}^{n}\left(j\right)\right)$ and from Lemma 3, if ${\hat{x}}_{R}^{n}\left(j\right)\in T_{\delta }^{n}\left({\hat{X}}_{R}\right)$ and $x_{R}^{n}\in T_{\delta_{1}}^{n}\left(X_{R}|{\hat{x}}_{R}^{n}\left(j\right)\right)$, then $\left(x_{R}^{n},{\hat{x}}_{R}^{n}\left(j\right)\right)\in T_{\delta +\delta_{1}}^{n}\left(X_{R},{\hat{X}}_{R}\right)$.
(ii) $j=M_{n}$:
  (A16) $$\begin{array}{cc} d(x_{R}^{n},{\hat{x}}_{R}^{n}\left(M_{n}\right))\; & =\frac{1}{n}\sum_{i=1}^{n}d\left(x_{R,i},{\hat{x}}_{R,i}\right)\\ \; & \overset{\left(f\right)}{\leq }D_{\max}, \end{array}$$
  where
  (f) is due to the definition of $D_{\max}≔\max_{a\in X_{R},b\in {\hat{X}}_{R}}d\left(a,b\right)$.

We consider $\mathrm{Pr}\{J_{n}=M_{n}\}$. From (A14),

(A17) $$\begin{array}{cc} \mathrm{Pr}\{J_{n}=M_{n}\}\; & =\mathrm{Pr}\{X_{E}^{n}\in A\left(M_{n}\right)\}\\ \; & =\mathrm{Pr}\left\{X_{E}^{n}\notin \overset{M_{n}-1}{\underset{j=1}{⋃}}A\left(j\right)\right\}\\ \; & \leq \left(2\right|X_{E}\left|+1\right)e^{-2\delta^{2}n}. \end{array}$$

Therefore, we can confirm

(A18) $$\begin{array}{c} \lim_{n\to \infty }\mathrm{Pr}\left\{J_{n}=M_{n}\right\}=0. \end{array}$$

From (i) and (ii), we can evaluate utility $u_{n}$ as below.

(A19) $$\begin{array}{cc} u_{n} & ≔E\left[d(X_{R}^{n},{\hat{X}}_{R}^{n})\right]\\ \; & \leq \sum_{j=1}^{M_{n}-1}\mathrm{Pr}\left\{J_{n}=j\right\}\cdot (E\left[d\left(X_{R},{\hat{X}}_{R}\right)\right]\\ \; & \;+\left(\delta +\delta_{1}\right)|X_{R}\left|\cdot \right|{\hat{X}}_{R}|D_{\max})+\mathrm{Pr}\left\{J_{n}=M_{n}\right\}\cdot D_{\max}\\ \; & \overset{\left(g\right)}{\leq }E\left[d\left(X_{R},{\hat{X}}_{R}\right)\right]+\left(\delta +\delta_{1}\right)|X_{R}\left|\cdot \right|{\hat{X}}_{R}|D_{\max}+\tau \end{array}$$

for all sufficiently large *n*, where

(g) follows from (A18).

Thus, we obtain (58).

We can evaluate the privacy leakage against the encoder as shown below.

(A20) $$\begin{array}{cc} e_{n}\; & ≔\frac{1}{n}I\left(X_{H}^{n};X_{E}^{n}\right)\\ \; & \overset{\left(h\right)}{=}\frac{1}{n}\sum_{i=1}^{n}I\left(X_{H,j};X_{E}^{n}|X_{H}^{j-1}\right)\\ \; & \overset{\left(i\right)}{=}\frac{1}{n}\sum_{i=1}^{n}I\left(X_{H,j};X_{E,j}\right)\\ \; & \overset{\left(j\right)}{=}I\left(X_{H};X_{E}\right), \end{array}$$

where

(h) is due to chain rule for mutual information and
(i), (j) follows because $i.i.d.\;P_{X_{K}^{n}}$.

Thus, we have (59).

Next, we show that the probability that random vector $X_{K}^{n}$ is not included in the set ${⋃}_{j=1}^{M_{n}-1}\tilde{A}\left(j\right)$ is sufficiently small. First, notice that

(A21) $$\begin{array}{cc} x_{K}^{n}\notin \overset{M_{n}-1}{\underset{j=1}{⋃}}\tilde{A}\left(j\right)⟹\; & x_{E}^{n}\notin \overset{M_{n}-1}{\underset{j=1}{⋃}}A\left(j\right)\\ \; & \;\mathrm{or}\\ \; & x_{E}^{n}\in A\left(j_{0}\right),\\ \; & \left(x_{E}^{n},x_{E^{c}}^{n}\right)\notin T_{2\delta }^{n}\left(X_{K}|{\hat{x}}_{R}^{n}\left(j_{0}\right)\right)\\ \; & \mathrm{for}\;j_{0}=f_{n}\left(x_{E}^{n}\right), \end{array}$$

where $j_{0}$ is the index such that $f_{n}\left(x_{E}^{n}\right)=j_{0}$ for $1\leq j_{0}\leq M_{n}-1$. Therefore, by the union upper bound,

(A22) $$\begin{array}{cc} \; & \mathrm{Pr}\left\{X_{K}^{n}\notin \overset{M_{n}-1}{\underset{j=1}{⋃}}\tilde{A}\left(j\right)\right\}\\ \; & \leq \mathrm{Pr}\left\{X_{E}^{n}\notin \overset{M_{n}-1}{\underset{j=1}{⋃}}A\left(j\right)\right\}\\ \; & \;+\mathrm{Pr}\{X_{E}^{n}\in A\left(j_{0}\right),\left(X_{E}^{n},X_{E^{c}}^{n}\right)\notin T_{2\delta }^{n}\left(X_{K}|{\hat{x}}_{R}^{n}\left(j_{0}\right)\right)\\ \; & \;\mathrm{for}\;j_{0}=f_{n}\left(X_{E}^{n}\right)\}. \end{array}$$

We evaluate each term in (A22).

(i) The first term:
  (A23) $$\begin{array}{cc} \mathrm{Pr}\left\{X_{E}^{n}\notin \overset{M_{n}-1}{\underset{j=1}{⋃}}A\left(j\right)\right\}\; & \overset{\left(k\right)}{\leq }\left(2\right|X_{E}\left|+1\right)e^{-2\delta^{2}n}, \end{array}$$
  where
  (k) is because of (A14).
(ii) The second term:
  If the event in the second term occurs, $x_{E}^{n}\in T_{\delta }^{n}\left(X_{E}|{\hat{x}}_{R}^{n}\left(j_{0}\right)\right)$ and $\left(x_{E}^{n},x_{E^{c}}^{n}\right)\notin T_{2\delta }^{n}\left(X_{K}|{\hat{x}}_{R}^{n}\left(j_{0}\right)\right)$. Therefore, from Lemma 5, $x_{E^{c}}^{n}\notin T_{\delta }^{n}\left(X_{E^{c}}|x_{E}^{n},{\hat{x}}_{R}^{n}\left(j_{0}\right)\right)$ holds. Hence,
  (A24) $$\begin{array}{cc} \; & \mathrm{Pr}\{X_{E}^{n}\in A\left(j_{0}\right),\left(X_{E}^{n},X_{E^{c}}^{n}\right)\notin T_{2\delta }^{n}\left(X_{K}|{\hat{x}}_{R}^{n}\left(j_{0}\right)\right)\\ \; & \;\mathrm{for}\;j_{0}=f_{n}\left(X_{E}^{n}\right)\}\\ \; & \leq \mathrm{Pr}\{X_{E}^{n}\in A\left(j_{0}\right),X_{E^{c}}^{n}\notin T_{\delta }^{n}\left(X_{E^{c}}|X_{E}^{n},{\hat{x}}_{R}^{n}\left(j_{0}\right)\right)\}\\ \; & \leq \sum_{j=1}^{M_{n}-1}\sum_{x_{E}^{n}\in A\left(j\right)}\mathrm{Pr}\left\{X_{E}^{n}=x_{E}^{n}\right\}\cdot \\ \; & \;\mathrm{Pr}\{X_{E^{c}}^{n}\notin T_{\delta }^{n}\left(X_{E^{c}}|x_{E}^{n},{\hat{x}}_{R}^{n}\left(j\right)\right)|X_{E}^{n}=x_{E}^{n}\}\\ \; & \overset{\left(l\right)}{=}\sum_{j=1}^{M_{n}-1}\sum_{x_{E}^{n}\in A\left(j\right)}\mathrm{Pr}\left\{X_{E}^{n}=x_{E}^{n}\right\}\cdot \\ \; & \;\mathrm{Pr}\{X_{E^{c}}^{n}\notin T_{\delta }^{n}\left(X_{E^{c}}|x_{E}^{n},{\hat{x}}_{R}^{n}\left(j\right)\right)|X_{E}^{n}=x_{E}^{n},{\hat{X}}_{R}^{n}={\hat{x}}_{R}^{n}\left(j\right)\}\\ \; & \overset{\left(m\right)}{\leq }\sum_{j=1}^{M_{n}-1}\sum_{x_{E}^{n}\in A\left(j\right)}\mathrm{Pr}\left\{X_{E}^{n}=x_{E}^{n}\right\}\cdot 2|X_{E^{c}}\left|\cdot \right|X_{E}\left|\cdot \right|{\hat{X}}_{R}|e^{-2\delta^{2}n}\\ \; & \overset{\left(n\right)}{\leq }2|X_{K}\left|\cdot \right|{\hat{X}}_{R}|e^{-2\delta^{2}n}, \end{array}$$
  where
  (l) is due to the Markov chain $X_{E^{c}}^{n}-X_{E}^{n}-{\hat{X}}_{R}^{n}$,
  (m) follows since $x_{E}^{n}\in T_{\delta }^{n}\left(X_{E}|{\hat{x}}_{R}^{n}\left(j_{0}\right)\right)$ and Lemma 6, and
  (n) follows because A(j) are disjoint for each *j*.

From (A22)–(A24),

(A25) $$\begin{array}{c} \mathrm{Pr}\left\{X_{K}^{n}\notin \overset{M_{n}-1}{\underset{j=1}{⋃}}\tilde{A}\left(j\right)\right\}\leq 4|X_{K}\left|\cdot \right|{\hat{X}}_{R}|e^{-2\delta^{2}n}. \end{array}$$

Therefore, for sufficiently large *n*,

(A26) $$\begin{array}{c} \mathrm{Pr}\left\{X_{K}^{n}\notin \overset{M_{n}-1}{\underset{j=1}{⋃}}\tilde{A}\left(j\right)\right\}\leq \tau , \end{array}$$

and we obtain (61).

From Lemma 1, for sufficiently large *n* to stochastic matrix $W\;:\;{\hat{X}}_{R}\to X_{K}$ and ${\hat{x}}_{R}^{n}\left(j\right)\in T_{\delta }^{n}\left({\hat{X}}_{R}\right)$ we can show that

(A27) $$\begin{array}{cc} \; & \left|\frac{1}{n}\log\left|T_{\delta_{2}}^{n}\left(X_{K}|{\hat{x}}_{R}^{n}\left(j\right)\right)\right|-H\left(X_{K}|{\hat{X}}_{R}\right)\right|\leq \tau ,\\ \; & \delta_{2}≔\frac{\delta }{|X_{E^{c}}|}. \end{array}$$

We can also show from (A27) that

(A28) $$\begin{array}{c} 2^{n\{H\left(X_{K}|{\hat{X}}_{R}\right)-\tau \}}\leq \left|T_{\delta_{2}}^{n}\left(X_{K}|{\hat{x}}_{R}^{n}\left(j\right)\right)\right|\leq 2^{n\{H\left(X_{K}|{\hat{X}}_{R}\right)+\tau \}}. \end{array}$$

From the definition of $\tilde{A}\left(j\right)$ and $T_{\delta_{2}}^{n}\left(X_{K}|{\hat{x}}_{R}^{n}\left(j\right)\right)$ and Lemma 3, for $j=1,2,\ldots ,M_{n}-1$, we have

(A29) $$\begin{array}{cc} x_{K}^{n}\in \tilde{A}\left(j\right) & ⟺\;\left\{\begin{array}{c} x_{E}^{n}\in T_{\delta }^{n}\left(X_{E}|{\hat{x}}_{R}^{n}\left(j\right)\right)\\ x_{K}^{n}\in T_{2\delta }^{n}\left(X_{K}|{\hat{x}}_{R}^{n}\left(j\right)\right) \end{array}\right. \end{array}$$

(A30) $$\begin{array}{cc} x_{K}^{n}\in T_{\delta_{2}}^{n}\left(X_{K}|{\hat{x}}_{R}^{n}\left(j\right)\right) & ⟹\left\{\begin{array}{c} x_{E}^{n}\in T_{\delta }^{n}\left(X_{E}|{\hat{x}}_{R}^{n}\left(j\right)\right)\\ x_{K}^{n}\in T_{2\delta }^{n}\left(X_{K}|{\hat{x}}_{R}^{n}\left(j\right)\right) \end{array}\right. \end{array}$$

This means

(A31) $$\begin{array}{cc} T_{\delta_{2}}^{n}\left(X_{K}|{\hat{x}}_{R}^{n}\left(j\right)\right) & \subseteq \tilde{A}\left(j\right)\\ ⟹|T_{\delta_{2}}^{n}\left(X_{K}|{\hat{x}}_{R}^{n}\left(j\right)\right)| & \leq |\tilde{A}\left(j\right)|. \end{array}$$

Therefore, from (A28) and (A31),

(A32) $$\begin{array}{c} |\tilde{A}\left(j\right)|\geq 2^{n\{H\left(X_{K}|{\hat{X}}_{R}\right)-\tau \}}, \end{array}$$

and we obtain (62).

## Appendix D. Derivation of Inequality in Equation (63)

We derive the inequality in (63). To write notation concisely, for every $x_{H}^{n}\in X_{H}^{n}$ and each $j=1,2,\ldots ,M_{n}$, we define $P_{n}\left(j\right)$, $Q_{n}\left(j\right)$, ${\tilde{P}}_{n}\left(x_{H}^{n},j\right)$, and ${\tilde{Q}}_{n}\left(x_{H}^{n},j\right)$ as follows:

(A33) $$\begin{array}{cc} P_{n}(j)≔ & \mathrm{Pr}\{X_{K}^{n}\in B\left(j\right)\}, \end{array}$$

(A34) $$\begin{array}{cc} Q_{n}(j)≔ & \mathrm{Pr}\{X_{K}^{n}\in \tilde{A}\left(j\right)\}, \end{array}$$

(A35) $$\begin{array}{cc} {\tilde{P}}_{n}(x_{H}^{n},j)≔ & \mathrm{Pr}\{X_{H}^{n}=x_{H}^{n},X_{K}^{n}\in B\left(j\right)\}, \end{array}$$

(A36) $$\begin{array}{cc} {\tilde{Q}}_{n}(x_{H}^{n},j)≔ & \mathrm{Pr}\{X_{H}^{n}=x_{H}^{n},X_{K}^{n}\in \tilde{A}\left(j\right)\}. \end{array}$$

Then, using the notation in [5], we can write each entropy as

(A37) $$\begin{array}{cc} H(X_{K}^{n}\in B\left(J_{n}\right)) & =H(P_{n}), \end{array}$$

(A38) $$\begin{array}{cc} H(X_{K}^{n}\in \tilde{A}\left(J_{n}\right)) & =H(Q_{n}), \end{array}$$

(A39) $$\begin{array}{cc} H(X_{H}^{n},X_{K}^{n}\in B\left(J_{n}\right)) & =H({\tilde{P}}_{n}), \end{array}$$

(A40) $$\begin{array}{cc} H(X_{H}^{n},X_{K}^{n}\in \tilde{A}\left(J_{n}\right)) & =H({\tilde{Q}}_{n}). \end{array}$$

The variational distance between distributions $P_{n}$ and $Q_{n}$ is

(A41) $$\begin{array}{cc} d_{v}\left(P_{n},Q_{n}\right)\; & =\sum_{j=1}^{M_{n}}\left|P_{n}\left(j\right)-Q_{n}\left(j\right)\right|\\ \; & =\sum_{j=1}^{M_{n}-1}\left|P_{n}\left(j\right)-Q_{n}\left(j\right)\right|\\ \; & \;+|P_{n}\left(M_{n}\right)-Q_{n}\left(M_{n}\right)|. \end{array}$$

We evaluate each term in (A41).

(i) The first term:
  (A42) $$\begin{array}{cc} \; & \sum_{j=1}^{M_{n}-1}\left|P_{n}\left(j\right)-Q_{n}\left(j\right)\right|\\ \; & =\sum_{j=1}^{M_{n}-1}\mathrm{Pr}\left\{X_{K}^{n}\in B\left(j\right)\setminus \tilde{A}\left(j\right)\right\}\\ \; & \overset{\left(a\right)}{=}\mathrm{Pr}\left\{X_{K}^{n}\in \overset{M_{n}-1}{\underset{j=1}{⋃}}B\left(j\right)\setminus \tilde{A}\left(j\right)\right\}\\ \; & =\mathrm{Pr}\left\{X_{K}^{n}\in \overset{M_{n}-1}{\underset{j=1}{⋃}}B\left(j\right)\right\}-\mathrm{Pr}\left\{X_{K}^{n}\in \overset{M_{n}-1}{\underset{j=1}{⋃}}\tilde{A}\left(j\right)\right\}\\ \; & =\left(1-\mathrm{Pr}\left\{X_{K}^{n}\in \overset{M_{n}-1}{\underset{j=1}{⋃}}\tilde{A}\left(j\right)\right\}\right)\\ \; & \;-\left(1-\mathrm{Pr}\left\{X_{K}^{n}\in \overset{M_{n}-1}{\underset{j=1}{⋃}}B\left(j\right)\right\}\right)\\ \; & =\mathrm{Pr}\left\{X_{K}^{n}\notin \overset{M_{n}-1}{\underset{j=1}{⋃}}\tilde{A}\left(j\right)\right\}-\mathrm{Pr}\left\{X_{K}^{n}\notin \overset{M_{n}-1}{\underset{j=1}{⋃}}B\left(j\right)\right\}\\ \; & \leq \mathrm{Pr}\left\{X_{K}^{n}\notin \overset{M_{n}-1}{\underset{j=1}{⋃}}\tilde{A}\left(j\right)\right\}\\ \; & \overset{\left(b\right)}{\leq }\tau , \end{array}$$
  where
  (a) follows because $B\left(j\right)\setminus \tilde{A}\left(j\right)$ is disjoint for each $j=1,2,\ldots ,M_{n}-1$,
  (b) is owing to (61).
(ii) The second term:
  (A43) $$\begin{array}{cc} |P_{n}\left(M_{n}\right)-Q_{n}\left(M_{n}\right)|\; & \overset{\left(c\right)}{=}Q_{n}\left(M_{n}\right)-P_{n}\left(M_{n}\right)\\ \; & \leq Q_{n}\left(M_{n}\right)\\ \; & =\mathrm{Pr}\left\{X_{K}^{n}\notin \overset{M_{n}-1}{\underset{j=1}{⋃}}\tilde{A}\left(j\right)\right\}\\ \; & \overset{\left(d\right)}{\leq }\tau , \end{array}$$
  where
  (c) follows because $B\left(M_{n}\right)\subseteq \tilde{A}\left(M_{n}\right)$ and
  (d) follows from (61).

From (A42) and (A43), the variational distance between $P_{n}$ and $Q_{n}$ is bounded from above as

(A44) $$\begin{array}{cc} d_{v}\left(P_{n},Q_{n}\right)\; & \leq \tau +\tau \\ \; & =2\tau . \end{array}$$

Next, the variational distance between distributions ${\tilde{P}}_{n}$ and ${\tilde{Q}}_{n}$ is

(A45) $$\begin{array}{cc} d_{v}\left({\tilde{P}}_{n},{\tilde{Q}}_{n}\right)\; & =\sum_{j=1}^{M_{n}}\sum_{x_{H}^{n}\in X_{H}^{n}}\left|{\tilde{P}}_{n}\left(x_{H}^{n},j\right)-{\tilde{Q}}_{n}\left(x_{H}^{n},j\right)\right|\\ \; & =\sum_{j=1}^{M_{n}-1}\sum_{x_{H}^{n}\in X_{H}^{n}}\left|{\tilde{P}}_{n}\left(x_{H}^{n},j\right)-{\tilde{Q}}_{n}\left(x_{H}^{n},j\right)\right|\\ \; & \;+\sum_{x_{H}^{n}\in X_{H}^{n}}\left|{\tilde{P}}_{n}\left(x_{H}^{n},M_{n}\right)-{\tilde{Q}}_{n}\left(x_{H}^{n},M_{n}\right)\right|. \end{array}$$

We evaluate each term in (A45).

(i) The first term:
  (A46) $$\begin{array}{cc} \; & \sum_{j=1}^{M_{n}-1}\sum_{x_{H}^{n}\in X_{H}^{n}}\left|{\tilde{P}}_{n}\left(x_{H}^{n},j\right)-{\tilde{Q}}_{n}\left(x_{H}^{n},j\right)\right|\\ \; & =\sum_{j=1}^{M_{n}-1}\sum_{x_{H}^{n}\in X_{H}^{n}}\mathrm{Pr}\left\{X_{H}^{n}=x_{H}^{n},X_{K}^{n}\in B\left(j\right)\setminus \tilde{A}\left(j\right)\right\}\\ \; & \overset{\left(e\right)}{=}\sum_{x_{H}^{n}\in X_{H}^{n}}\mathrm{Pr}\left\{X_{H}^{n}=x_{H}^{n},X_{K}^{n}\in \overset{M_{n}-1}{\underset{j=1}{⋃}}B\left(j\right)\setminus \tilde{A}\left(j\right)\right\}\\ \; & =\mathrm{Pr}\left\{X_{K}^{n}\in \overset{M_{n}-1}{\underset{j=1}{⋃}}B\left(j\right)\setminus \tilde{A}\left(j\right)\right\}\\ \; & =\mathrm{Pr}\left\{X_{K}^{n}\in \overset{M_{n}-1}{\underset{j=1}{⋃}}B\left(j\right)\right\}-\mathrm{Pr}\left\{X_{K}^{n}\in \overset{M_{n}-1}{\underset{j=1}{⋃}}\tilde{A}\left(j\right)\right\}\\ \; & =\left(1-\mathrm{Pr}\left\{X_{K}^{n}\in \overset{M_{n}-1}{\underset{j=1}{⋃}}\tilde{A}\left(j\right)\right\}\right)\\ \; & \;-\left(1-\mathrm{Pr}\left\{X_{K}^{n}\in \overset{M_{n}-1}{\underset{j=1}{⋃}}B\left(j\right)\right\}\right)\\ \; & =\mathrm{Pr}\left\{X_{K}^{n}\notin \overset{M_{n}-1}{\underset{j=1}{⋃}}\tilde{A}\left(j\right)\right\}-\mathrm{Pr}\left\{X_{K}^{n}\notin \overset{M_{n}-1}{\underset{j=1}{⋃}}B\left(j\right)\right\}\\ \; & \leq \mathrm{Pr}\left\{X_{K}^{n}\notin \overset{M_{n}-1}{\underset{j=1}{⋃}}\tilde{A}\left(j\right)\right\}\\ \; & \overset{\left(f\right)}{\leq }\tau , \end{array}$$
  where
  (e) follows since $B\left(j\right)\setminus \tilde{A}\left(j\right)$ is disjoint for each $j=1,2,\ldots ,M_{n}-1$,
  (f) is due to (61).
(ii) The second term:
  (A47) $$\begin{array}{cc} \; & \sum_{x_{H}^{n}\in X_{H}^{n}}\left|{\tilde{P}}_{n}\left(x_{H}^{n},M_{n}\right)-{\tilde{Q}}_{n}\left(x_{H}^{n},M_{n}\right)\right|\\ \; & \overset{\left(g\right)}{=}\sum_{x_{H}^{n}\in X_{H}^{n}}\left({\tilde{Q}}_{n}\left(x_{H}^{n},M_{n}\right)-{\tilde{P}}_{n}\left(x_{H}^{n},M_{n}\right)\right)\\ \; & \leq \sum_{x_{H}^{n}\in X_{H}^{n}}{\tilde{Q}}_{n}\left(x_{H}^{n},M_{n}\right)\\ \; & =Q_{n}\left(M_{n}\right)\\ \; & =\mathrm{Pr}\left\{X_{K}^{n}\notin \overset{M_{n}-1}{\underset{j=1}{⋃}}\tilde{A}\left(j\right)\right\}\\ \; & \overset{\left(h\right)}{\leq }\tau , \end{array}$$
  where
  (g) follows because $B\left(M_{n}\right)\subseteq \tilde{A}\left(M_{n}\right)$ and
  (h) is due to (61).

From (A46) and (A47), the variational distance between ${\tilde{P}}_{n}$ and ${\tilde{Q}}_{n}$ is bounded from above as

(A48) $$\begin{array}{cc} d_{v}\left({\tilde{P}}_{n},{\tilde{Q}}_{n}\right)\; & \leq \tau +\tau \\ \; & =2\tau . \end{array}$$

As a result, from Lemma 2 and the relation of each entropy,

(A49) $$\begin{array}{cc} \; & |H\left(X_{K}^{n}\in B\left(J_{n}\right)\right)-H\left(X_{K}^{n}\in \tilde{A}\left(J_{n}\right)\right)|\leq -2\tau \log\frac{2\tau }{M_{n}},\\ \; & |H\left(X_{H}^{n},X_{K}^{n}\in B\left(J_{n}\right)\right)-H\left(X_{H}^{n},X_{K}^{n}\in \tilde{A}\left(J_{n}\right)\right)| \end{array}$$

(A50) $$\begin{array}{cc} \; & \leq -2\tau \log\frac{2\tau }{|X_{H}{|}^{n}\cdot M_{n}}. \end{array}$$

From (A49), (A50), and the chain rule of entropy,

(A51) $$\begin{array}{cc} \; & \left|H(X_{H}^{n}|X_{K}^{n}\in B\left(J_{n}\right))-H\left(X_{H}^{n}|X_{K}^{n}\in \tilde{A}\left(J_{n}\right)\right)\right|\\ \; & =|\{H\left(X_{H}^{n},X_{K}^{n}\in B\left(J_{n}\right)\right)-H\left(X_{K}^{n}\in B\left(J_{n}\right)\right)\}\\ \; & \;-\{H\left(X_{H}^{n},X_{K}^{n}\in \tilde{A}\left(J_{n}\right)\right)-H\left(X_{K}^{n}\in \tilde{A}\left(J_{n}\right)\right)\}|\\ \; & =|\{H\left(X_{H}^{n},X_{K}^{n}\in B\left(J_{n}\right))-H\left(X_{H}^{n},X_{K}^{n}\in \tilde{A}\left(J_{n}\right)\right)\right\}\\ \; & \;+\{H\left(X_{K}^{n}\in \tilde{A}\left(J_{n}\right)\right)-H\left(X_{K}^{n}\in B\left(J_{n}\right)\right)\}|\\ \; & \overset{\left(i\right)}{\leq }|H(X_{H}^{n},X_{K}^{n}\in B\left(J_{n}\right))-H\left(X_{H}^{n},X_{K}^{n}\in \tilde{A}\left(J_{n}\right)\right)|\\ \; & \;+|H\left(X_{K}^{n}\in \tilde{A}\left(J_{n}\right)\right)-H\left(X_{K}^{n}\in B\left(J_{n}\right)\right)|\\ \; & \leq -2\tau \log\frac{2\tau }{M_{n}}-2\tau \log\frac{2\tau }{|X_{H}{|}^{n}\cdot M_{n}}\\ \; & =-4\tau \log\frac{2\tau }{|X_{H}{|}^{n}\cdot M_{n}}\\ \; & =4\tau \log\frac{|X_{H}{|}^{n}\cdot M_{n}}{2\tau }, \end{array}$$

where

(i) is because of the triangle inequality.

Therefore, we obtain

(A52) $$\begin{array}{cc} \frac{1}{n}H\left(X_{H}^{n}|J_{n}\right)\; & =\frac{1}{n}H\left(X_{H}^{n}|X_{K}^{n}\in B\left(J_{n}\right)\right)\\ \; & \geq \frac{1}{n}H\left(X_{H}^{n}|X_{K}^{n}\in \tilde{A}\left(J_{n}\right)\right)-\frac{4\tau }{n}\log\frac{|X_{H}{|}^{n}\cdot M_{n}}{2\tau }\\ \; & \overset{\left(j\right)}{>}\frac{1}{n}H\left(X_{H}^{n}|X_{K}^{n}\in \tilde{A}\left(J_{n}\right)\right)-\frac{4\tau }{n}\log\frac{|X_{H}{|}^{n}\cdot 2^{nR}}{{\left(2\tau \right)}^{n}}\\ \; & =\frac{1}{n}H\left(X_{H}^{n}|X_{K}^{n}\in \tilde{A}\left(J_{n}\right)\right)-4\tau \log\frac{|X_{H}|\cdot 2^{R}}{2\tau }, \end{array}$$

where

(j) follows from the definition that $M_{n}=2^{nR}$ and 2τ<1.

We complete the derivation of (63).

## Appendix E. Proof of Equation (65)

First of all, we shall show

(A53) $$\begin{array}{c} x_{K}^{n}\in \tilde{A}\left(j\right)⟹x_{R}^{n}\in T_{\delta_{3}}^{n}\left(X_{R}|x_{H}^{n},{\hat{x}}_{R}^{n}\left(j\right)\right),\\ \delta_{3}≔\left(\right|X_{H}\left|+1\right)\cdot 2\delta . \end{array}$$

By the definition of $\tilde{A}\left(j\right)$,

(A54) $$\begin{array}{c} \tilde{A}\left(j\right)\subseteq T_{2\delta }^{n}\left(X_{K}|{\hat{x}}_{R}^{n}\left(j\right)\right)\;\;\;\mathrm{for}\;j=1,2,\ldots ,M_{n}-1. \end{array}$$

Thus, from Lemma 4, any $x_{R}^{n}$ such that $\left(x_{R}^{n},x_{H}^{n}\right)\in \tilde{A}\left(j\right)$ satisfies

(A55) $$\begin{array}{c} x_{R}^{n}\in T_{\delta_{3}}^{n}\left(X_{R}|x_{H}^{n},{\hat{x}}_{R}^{n}\left(j\right)\right). \end{array}$$

That is, given $x_{H}^{n}\in X_{H}^{n}$ and ${\hat{x}}_{R}^{n}\left(j\right)\in {\hat{X}}_{R}^{n}$, $x_{R}^{n}\in X_{R}^{n}$ and $x_{K}^{n}=\left(x_{R}^{n},x_{H}^{n}\right)\in \tilde{A}\left(j\right)$ are conditional strongly typical sequences. Then, we obtain (A53), and

(A56) $$\begin{array}{cc} \; & \sum_{x_{R}^{n}\;:\;\left(x_{R}^{n},x_{H}^{n}\right)\in \tilde{A}\left(j\right)}\mathrm{Pr}\left\{X_{R}^{n}=x_{R}^{n}|X_{H}^{n}=x_{H}^{n}\right\}\mathrm{Pr}\left\{X_{H}^{n}=x_{H}^{n}\right\}\\ \leq & \sum_{x_{R}^{n}\in T_{\delta_{3}}^{n}\left(X_{R}|x_{H}^{n},{\hat{x}}_{R}^{n}\left(j\right)\right)}\mathrm{Pr}\left\{X_{R}^{n}=x_{R}^{n}|X_{H}^{n}=x_{H}^{n}\right\}\mathrm{Pr}\left\{X_{H}^{n}=x_{H}^{n}\right\}. \end{array}$$

Therefore, we obtain (65).

## Appendix F. Proof of the Existence of Code Satisfying Equations (111)–(116)

We first set $M_{n}≔2^{nR}$ and $r_{n}≔\frac{1}{n}\log M_{n}$. Then, we obviously have (111).

From the union upper bound,

(A57) $$\begin{array}{cc} \; & \mathrm{Pr}\left\{X_{E}^{n}\notin \overset{M_{n}-1}{\underset{j=1}{⋃}}A\left(j\right)\right\}\\ \; & \leq \mathrm{Pr}\{X_{E}^{n}\notin T_{\delta }^{n}\left(X_{E}\right)\}\\ \; & \;+\mathrm{Pr}\{X_{E}^{n}\in T_{\delta }^{n}\left(X_{E}\right),X_{E}^{n}\notin T_{\delta }^{n}\left(X_{E}|{\hat{x}}_{R}^{n}\left(j\right)\right)\\ \; & \;\;\mathrm{for}\;\mathrm{all}\;j=1,2,\ldots ,M_{n}-1\}. \end{array}$$

From Lemma 6, the first term in (A57) is bounded as

(A58) $$\begin{array}{c} \mathrm{Pr}\left\{X_{E}^{n}\notin T_{\delta }^{n}\left(X_{E}\right)\right\}\leq 2\left|X_{E}\right|e^{-2\delta^{2}n}. \end{array}$$

We consider the expectation of the second term in (A57) by random coding. Hereafter, we denote the random variable corresponding to the reproduced sequence ${\hat{x}}_{R}^{n}\left(j\right)$ as ${\hat{X}}_{R}^{n}\left(j\right)$. For notational simplicity, we use the abbreviation

(A59) $$\begin{array}{cc} \; & \mathrm{Pr}\{X_{E}^{n}\notin T_{\delta }^{n}\left(X_{E}|{\hat{X}}_{R}^{n}\left(j\right)\right)\\ \; & \;\mathrm{for}\;\mathrm{all}\;j=1,2,\ldots ,M_{n}-1\left|X_{E}^{n}=x_{E}^{n}\right\}\\ \; & =\mathrm{Pr}\{x_{E}^{n}\notin T_{\delta }^{n}\left(X_{E}|{\hat{X}}_{R}^{n}\left(j\right)\right)\\ \; & \;\mathrm{for}\;\mathrm{all}\;j=1,2,\ldots ,M_{n}-1\}, \end{array}$$

and then

(A60) $$\begin{array}{cc} \; & E[\mathrm{Pr}\{X_{E}^{n}\in T_{\delta }^{n}\left(X_{E}\right),X_{E}^{n}\notin T_{\delta }^{n}\left(X_{E}|{\hat{X}}_{R}^{n}\left(j\right)\right)\\ \; & \;\mathrm{for}\;\mathrm{all}\;j=1,2,\ldots ,M_{n}-1\}]\\ \; & =\sum_{x_{E}^{n}\in T_{\delta }^{n}\left(X_{E}\right)}p\left(x_{E}^{n}\right)E[\mathrm{Pr}\{X_{E}^{n}\notin T_{\delta }^{n}\left(X_{E}|{\hat{X}}_{R}^{n}\left(j\right)\right)\\ \; & \;\mathrm{for}\;\mathrm{all}\;j=1,2,\ldots ,M_{n}-1\left|X_{E}^{n}=x_{E}^{n}\right\}]\\ \; & \overset{\left(a\right)}{=}\sum_{x_{E}^{n}\in T_{\delta }^{n}\left(X_{E}\right)}p\left(x_{E}^{n}\right)E[\mathrm{Pr}\{x_{E}^{n}\notin T_{\delta }^{n}\left(X_{E}|{\hat{X}}_{R}^{n}\left(j\right)\right)\\ \; & \;\mathrm{for}\;\mathrm{all}\;j=1,2,\ldots ,M_{n}-1\}]\\ \; & =\sum_{x_{E}^{n}\in T_{\delta }^{n}\left(X_{E}\right)}p\left(x_{E}^{n}\right)\left(\prod_{j=1}^{M_{n}-1}E\left[\mathrm{Pr}\{x_{E}^{n}\notin T_{\delta }^{n}\left(X_{E}|{\hat{X}}_{R}^{n}\left(j\right)\right)\}\right]\right)\\ \; & \overset{\left(b\right)}{=}\sum_{x_{E}^{n}\in T_{\delta }^{n}\left(X_{E}\right)}p\left(x_{E}^{n}\right){\left(E\left[\mathrm{Pr}\{x_{E}^{n}\notin T_{\delta }^{n}\left(X_{E}|{\hat{X}}_{R}^{n}\left(1\right)\right)\}\right]\right)}^{M_{n}-1}\\ \; & \overset{\left(c\right)}{\leq }\exp\left\{-2^{n(R-I\left(X_{E};{\hat{X}}_{R}\right)-\frac{1}{n}-\tau )}\right\}\\ \; & \overset{\left(d\right)}{\leq }\exp\left\{-2^{2\delta^{2}n}\right\}, \end{array}$$

where

(a) is owing to (A59),
(b) is due to the symmetry about indexes of random coding,
(c) follows from the same way as in ([31], Section 3.6.3), and
(d) because δ is fixed to satisfy (103).

From (A58) and (A60), we obtain

(A61) $$\begin{array}{c} E\left[\mathrm{Pr}\left\{X_{E}^{n}\notin \overset{M_{n}-1}{\underset{j=1}{⋃}}A\left(j\right)\right\}\right]\leq \left(2\right|X_{E}\left|+1\right)e^{-2\delta^{2}n}. \end{array}$$

Therefore, there exists at least one codebook satisfying (112) in the ensembles obtained using random coding.

Hereafter, codebook C is fixed to satisfy (112). That is, codebook C satisfies

(A62) $$\begin{array}{c} \mathrm{Pr}\left\{X_{E}^{n}\notin \overset{M_{n}-1}{\underset{j=1}{⋃}}A\left(j\right)\right\}\leq \left(2\right|X_{E}\left|+1\right)e^{-2\delta^{2}n}. \end{array}$$

For a fixed codebook C, we divide the sequences $x_{E}^{n}\in X_{E}^{n}$ into three categories.

- Strongly typical sequences $x_{E}^{n}\in T_{\delta }^{n}\left(X_{E}\right)$ such that there exists a codeword ${\hat{X}}_{R}^{n}\left(j_{o}\right)$ for some $j_{o}=1,2,\ldots ,M_{n}-1$ that is conditionally strongly typical with $x_{E}^{n}.$ In this case, from Lemma 3, $\left(x_{E},{\hat{x}}_{R}^{n}\left(j_{o}\right)\right)\in T_{2\delta }^{n}\left(X_{E},{\hat{X}}_{R}\left(j_{o}\right)\right)$. Since the codeword is jointly strongly typical with $x_{E}^{n}$, the continuity of the distortion as a function of the joint distribution ensures that they are also typical distortions (see [2], Chapters 10.5 and 10.6). Hence, the distortion between these $x_{E}^{n}$ and their codewords is bounded by $D+\delta^{\prime }$ where $\delta^{\prime }$ goes to 0 as n→∞. In the first-order analysis, that is, n→∞, we can regard $D+\delta^{\prime }$ as *D*.
- Strongly typical sequences $x_{E}^{n}\in T_{\delta }^{n}\left(X_{E}\right)$ such that $f_{n}\left(x_{E}^{n}\right)=M_{n}$.
- Non-strongly typical sequences $x_{E}^{n}\notin T_{\delta }^{n}\left(X_{E}\right)$.

The sequences in the second and third categories are encoded as $f_{n}\left(x_{E}^{n}\right)=M_{n}.$ The sequences of third categories are the sequences that can be bounded by such the distortion $d_{\max}$ as in excess of *D*. Then, the excess-distortion probability is evaluated as

(A63) $$\begin{array}{cc} \mathrm{Pr}\left\{\frac{1}{n}d\left(X_{R}^{n},{\hat{X}}_{R}^{n}\right)>D\right\}\; & <\mathrm{Pr}\left\{X_{E}^{n}\in A\left(M_{n}\right)\right\} \end{array}$$

(A64) $$\begin{array}{cc} \; & =\mathrm{Pr}\left\{X_{E}^{n}\notin \overset{M_{n}-1}{\underset{j=1}{⋃}}A\left(j\right)\right\} \end{array}$$

(A65) $$\begin{array}{cc} \; & \leq \left(2\right|X_{E}\left|+1\right)e^{-2\delta^{2}n}. \end{array}$$

Hence, for an appropriate choice of ϵ and *n*, we can ensure the excess-distortion probability of all badly represented sequences are as small as we want. We obtain (113).

We can evaluate privacy leakage against the encoder as below.

(A66) $$\begin{array}{cc} e_{n}\; & ≔\frac{1}{n}I\left(X_{H}^{n};X_{E}^{n}\right)\\ \; & \overset{\left(e\right)}{=}\frac{1}{n}\sum_{i=1}^{n}I\left(X_{H,j};X_{E}^{n}|X_{H}^{j-1}\right)\\ \; & \overset{\left(f\right)}{=}\frac{1}{n}\sum_{i=1}^{n}I\left(X_{H,j};X_{E,j}\right)\\ \; & \overset{\left(g\right)}{=}I\left(X_{H};X_{E}\right), \end{array}$$

where

(e) is due to chain rule for mutual information and
(f), (g) follows because $i.i.d.\;P_{X_{K}^{n}}$.

Thus, we have (114).

Next, we show that the probability that random vector $X_{K}^{n}$ is not included in the set ${⋃}_{j=1}^{M_{n}-1}\tilde{A}\left(j\right)$ and is sufficiently small. First, notice that

(A67) $$\begin{array}{cc} x_{K}^{n}\notin \overset{M_{n}-1}{\underset{j=1}{⋃}}\tilde{A}\left(j\right)⟹\; & x_{E}^{n}\notin \overset{M_{n}-1}{\underset{j=1}{⋃}}A\left(j\right)\\ \; & \;\mathrm{or}\\ \; & x_{E}^{n}\in A\left(j_{0}\right),\\ \; & \left(x_{E}^{n},x_{E^{c}}^{n}\right)\notin T_{2\delta }^{n}\left(X_{K}|{\hat{x}}_{R}^{n}\left(j_{0}\right)\right)\\ \; & \mathrm{for}\;j_{0}=f_{n}\left(x_{E}^{n}\right), \end{array}$$

where $j_{0}$ is the index such that $f_{n}\left(x_{E}^{n}\right)=j_{0}$ for $1\leq j_{0}\leq M_{n}-1$. Therefore, by the union’s upper bound,

(A68) $$\begin{array}{cc} \; & \mathrm{Pr}\left\{X_{K}^{n}\notin \overset{M_{n}-1}{\underset{j=1}{⋃}}\tilde{A}\left(j\right)\right\}\\ \; & \leq \mathrm{Pr}\left\{X_{E}^{n}\notin \overset{M_{n}-1}{\underset{j=1}{⋃}}A\left(j\right)\right\}\\ \; & \;+\mathrm{Pr}\{X_{E}^{n}\in A\left(j_{0}\right),\left(X_{E}^{n},X_{E^{c}}^{n}\right)\notin T_{2\delta }^{n}\left(X_{K}|{\hat{x}}_{R}^{n}\left(j_{0}\right)\right)\\ \; & \;\mathrm{for}\;j_{0}=f_{n}\left(X_{E}^{n}\right)\}. \end{array}$$

We evaluate each term in (A68).

(i) The first term:
  (A69) $$\begin{array}{cc} \mathrm{Pr}\left\{X_{E}^{n}\notin \overset{M_{n}-1}{\underset{j=1}{⋃}}A\left(j\right)\right\}\; & \overset{\left(h\right)}{\leq }\left(2\right|X_{E}\left|+1\right)e^{-2\delta^{2}n}, \end{array}$$
  where
  (h) is because of (A62).
(ii) The second term:
  If the event in the second term occurs, $x_{E}^{n}\in T_{\delta }^{n}\left(X_{E}|{\hat{x}}_{R}^{n}\left(j_{0}\right)\right)$ and $\left(x_{E}^{n},x_{E^{c}}^{n}\right)\notin T_{2\delta }^{n}\left(X_{K}|{\hat{x}}_{R}^{n}\left(j_{0}\right)\right)$. Therefore, from Lemma 5, $x_{E^{c}}^{n}\notin T_{\delta }^{n}\left(X_{E^{c}}|x_{E}^{n},{\hat{x}}_{R}^{n}\left(j_{0}\right)\right)$ holds. Hence,
  (A70) $$\begin{array}{cc} \; & \mathrm{Pr}\{X_{E}^{n}\in A\left(j_{0}\right),\left(X_{E}^{n},X_{E^{c}}^{n}\right)\notin T_{2\delta }^{n}\left(X_{K}|{\hat{x}}_{R}^{n}\left(j_{0}\right)\right)\\ \; & \;\mathrm{for}\;j_{0}=f_{n}\left(X_{E}^{n}\right)\}\\ \; & \leq \mathrm{Pr}\{X_{E}^{n}\in A\left(j_{0}\right),X_{E^{c}}^{n}\notin T_{\delta }^{n}\left(X_{E^{c}}|X_{E}^{n},{\hat{x}}_{R}^{n}\left(j_{0}\right)\right)\}\\ \; & \leq \sum_{j=1}^{M_{n}-1}\sum_{x_{E}^{n}\in A\left(j\right)}\mathrm{Pr}\left\{X_{E}^{n}=x_{E}^{n}\right\}\cdot \\ \; & \;\mathrm{Pr}\{X_{E^{c}}^{n}\notin T_{\delta }^{n}\left(X_{E^{c}}|x_{E}^{n},{\hat{x}}_{R}^{n}\left(j\right)\right)|X_{E}^{n}=x_{E}^{n}\}\\ \; & \overset{\left(i\right)}{=}\sum_{j=1}^{M_{n}-1}\sum_{x_{E}^{n}\in A\left(j\right)}\mathrm{Pr}\left\{X_{E}^{n}=x_{E}^{n}\right\}\cdot \\ \; & \;\mathrm{Pr}\{X_{E^{c}}^{n}\notin T_{\delta }^{n}\left(X_{E^{c}}|x_{E}^{n},{\hat{x}}_{R}^{n}\left(j\right)\right)|X_{E}^{n}=x_{E}^{n},{\hat{X}}_{R}^{n}={\hat{x}}_{R}^{n}\left(j\right)\}\\ \; & \overset{\left(j\right)}{\leq }\sum_{j=1}^{M_{n}-1}\sum_{x_{E}^{n}\in A\left(j\right)}\mathrm{Pr}\left\{X_{E}^{n}=x_{E}^{n}\right\}\cdot 2|X_{E^{c}}\left|\cdot \right|X_{E}\left|\cdot \right|{\hat{X}}_{R}|e^{-2\delta^{2}n}\\ \; & \overset{\left(k\right)}{\leq }2|X_{K}\left|\cdot \right|{\hat{X}}_{R}|e^{-2\delta^{2}n}, \end{array}$$
  where
  (i) is due to the Markov chain $X_{E^{c}}^{n}-X_{E}^{n}-{\hat{X}}_{R}^{n}$,
  (j) follows since $x_{E}^{n}\in T_{\delta }^{n}\left(X_{E}|{\hat{x}}_{R}^{n}\left(j_{0}\right)\right)$ and Lemma 6,
  (k) follows because A(j) is disjoint for each *j*.

From (A68)–(A70),

(A71) $$\begin{array}{c} \mathrm{Pr}\left\{X_{K}^{n}\notin \overset{M_{n}-1}{\underset{j=1}{⋃}}\tilde{A}\left(j\right)\right\}\leq 4|X_{K}\left|\cdot \right|{\hat{X}}_{R}|e^{-2\delta^{2}n}. \end{array}$$

Therefore, for sufficiently large *n*,

(A72) $$\begin{array}{c} \mathrm{Pr}\left\{X_{K}^{n}\notin \overset{M_{n}-1}{\underset{j=1}{⋃}}\tilde{A}\left(j\right)\right\}\leq \tau , \end{array}$$

and we obtain (115).

From Lemma 1, for sufficiently large *n* to stochastic matrix $W\;:\;{\hat{X}}_{R}\to X_{K}$ and ${\hat{x}}_{R}^{n}\left(j\right)\in T_{\delta }^{n}\left({\hat{X}}_{R}\right)$ we can show that

(A73) $$\begin{array}{cc} \; & \left|\frac{1}{n}\log\left|T_{\delta_{2}}^{n}\left(X_{K}|{\hat{x}}_{R}^{n}\left(j\right)\right)\right|-H\left(X_{K}|{\hat{X}}_{R}\right)\right|\leq \tau ,\\ \; & \delta_{2}≔\frac{\delta }{|X_{E^{c}}|}. \end{array}$$

We can also show from (A73) that

(A74) $$\begin{array}{c} 2^{n\{H\left(X_{K}|{\hat{X}}_{R}\right)-\tau \}}\leq \left|T_{\delta_{2}}^{n}\left(X_{K}|{\hat{x}}_{R}^{n}\left(j\right)\right)\right|\leq 2^{n\{H\left(X_{K}|{\hat{X}}_{R}\right)+\tau \}}. \end{array}$$

From the definition of $\tilde{A}\left(j\right)$ and $T_{\delta_{2}}^{n}\left(X_{K}|{\hat{x}}_{R}^{n}\left(j\right)\right)$ and Lemma 4, for $j=1,2,\ldots ,M_{n}-1$, we have

(A75) $$\begin{array}{cc} x_{K}^{n}\in \tilde{A}\left(j\right) & ⟺\;\left\{\begin{array}{c} x_{E}^{n}\in T_{\delta }^{n}\left(X_{E}|{\hat{x}}_{R}^{n}\left(j\right)\right)\\ x_{K}^{n}\in T_{2\delta }^{n}\left(X_{K}|{\hat{x}}_{R}^{n}\left(j\right)\right) \end{array}\right. \end{array}$$

(A76) $$\begin{array}{cc} x_{K}^{n}\in T_{\delta_{2}}^{n}\left(X_{K}|{\hat{x}}_{R}^{n}\left(j\right)\right) & ⟹\left\{\begin{array}{c} x_{E}^{n}\in T_{\delta }^{n}\left(X_{E}|{\hat{x}}_{R}^{n}\left(j\right)\right)\\ x_{K}^{n}\in T_{2\delta }^{n}\left(X_{K}|{\hat{x}}_{R}^{n}\left(j\right)\right) \end{array}\right. \end{array}$$

This means

(A77) $$\begin{array}{cc} T_{\delta_{2}}^{n}\left(X_{K}|{\hat{x}}_{R}^{n}\left(j\right)\right) & \subseteq \tilde{A}\left(j\right)\\ ⟹|T_{\delta_{2}}^{n}\left(X_{K}|{\hat{x}}_{R}^{n}\left(j\right)\right)| & \leq |\tilde{A}\left(j\right)|. \end{array}$$

Therefore, from (A74) and (A77),

(A78) $$\begin{array}{c} |\tilde{A}\left(j\right)|\geq 2^{n\{H\left(X_{K}|{\hat{X}}_{R}\right)-\tau \}}, \end{array}$$

and we obtain (116).
